# (*E*)-2-Benzylidenecyclanones: Part XX—Reaction of Cyclic Chalcone Analogs with Cellular Thiols: Unexpected Increased Reactivity of 4-Chromanone- Compared to 1-Tetralone Analogs in Thia-Michael Reactions †

**DOI:** 10.3390/molecules29235493

**Published:** 2024-11-21

**Authors:** Gábor Bognár, Fatemeh Kenari, Zoltán Pintér, Igor D. Borges, Ademir J. Camargo, Heibbe C. B. Oliveira, Flávio Olimpio Sanches-Neto, Valter H. Carvalho-Silva, Hamilton B. Napolitano, Pál Perjési

**Affiliations:** 1 Institute of Pharmaceutical Chemistry, University of Pécs, H-7624 Pécs, Hungary; bognar.gabor@pte.hu (G.B.); kenari.fatemeh@gmail.com (F.K.); pinter.zoltan@pte.hu (Z.P.); 2 Grupo de Química Teórica e Estrutural de Anápolis, Universidade Estadual de Goiás, Anápolis 75132-903, GO, Brazil; i.dalarmelino@gmail.com (I.D.B.); ajc@eug.br (A.J.C.); hamilton@ueg.br (H.B.N.); 3 Laboratório de Estrutura Eletrônica e Dinâmica Molecular, Universidade Federal de Goiás, Goiânia 74690-900, GO, Brazil; heibbe@ufg.br (H.C.B.O.); flavio_olimpio@outlook.com (F.O.S.-N.); 4 Instituto Federal de Educação, Ciência e Tecnologia de Goiás, Valparaíso de Goiás 72876-601, GO, Brazil; 5 Instituto de Química, Universidade de Brasília, Caixa Postal 4478, Brasília 70904-970, Brazil; 6 Laboratory for Modeling of Physical and Chemical Transformations, Research and Graduate Center, Goiás State University, Anápolis 75132-903, GO, Brazil; fatioleg@gmail.com

**Keywords:** chalcone, homoisoflavones, benzylidenechromanones, anticancer activity, glutathione, N-acetylcysteine, thia-Michael addition, molecular electrostatic, DFT calculations

## Abstract

In vitro relative cytotoxicity (IC_50_ (**IIb**)/IC_50_ (**IIIb**) of (*E*)-3-(4′-methylbenzylidene)-4-chromanone (**IIIb**) towards human Molt 4/C8 and CEM T-lymphocytes showed a >50-fold increase in comparison to those of the respective tetralone derivative (**IIb**). On the other hand, such an increase was not observed in the analogous 4-OCH_3_ (**IIc** and **IIIc**) derivatives. In order to study whether thiol reactivity—as a possible basis of the mechanism of action—correlates with the observed cytotoxicities, the kinetics of the non-enzyme catalyzed reactions with reduced glutathione (GSH) and N-acetylcysteine (NAC) of **IIIb** and **IIIc** were investigated. The reactivity of the compounds and the stereochemical outcome of the reactions were evaluated using high-pressure liquid chromatography-mass spectrometry (HPLC-MS). Molecular modeling calculations were performed to rationalize the unexpectedly higher thiol reactivity of the chromanones (**III**) compared to the carbocyclic analog tetralones (**II**). The results indicate the possible role of spontaneous thiol reactivity of compounds **III** in their recorded biological effects.

## 1. Introduction

Chalcones (I) are natural compounds, most often found in higher plants, especially in members of the *Leguminosae*, *Asteraceae*, and *Moraceae* families. These plants are often used in traditional medicine, so the anti-inflammatory, cytotoxic, antimicrobial, antifungal, antidiabetic, and chemopreventive effects of chalcones have been extensively studied in several laboratories [1]. Recognizing the multifaceted biological effects of natural chalcones, which can also be used pharmacologically, the synthesis and biological effects of a large number of synthetic chalcones have been investigated and are still under study. These results are summarized in several review papers [2,3,4,5].

**Figure 1:**
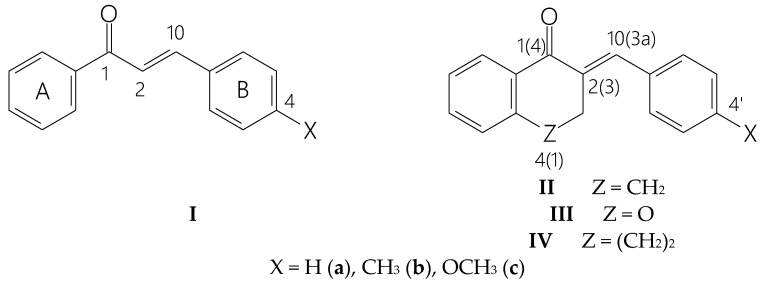
Structure of 4-X-chalcones (**I**), (*E*)-2-(4′-X-phenylmethylene)-1-tetralones (**II**), benzosuberones (**IV**), and (*E*)-3-(4′-X-phenylmethylene)-4-chromanones (**III**). Numbering of the general cyclic structures (**II**–**IV**) refers to series **II** and **IV**. Numbering in brackets refers to the compounds of **III**. The non-conventional numbering of **I** is only for comparison of the spectroscopic and quantum mechanical characteristics with the respective atoms of **II**–**IV**.

In previous studies, we reported on the synthesis, stereochemistry, and in vitro cancer cell cytotoxicity of chalcones (**I**) and cyclic chalcone analogs (**II**, **III**, **IV**) against human Molt 4/8 and CEM T-lymphocytes, as well as murine L1210 cells [6,7,8]. Among these, (*E*)-2-(X-benzylidene)-1-benzosuberones (**IV**) and (*E*)-3-(X-benzylidene)-4-chromanones (**III**) were the most prosperous class of compounds (see Figure 1 below).

Comparison of the cancer cell half-maximal inhibitory concentration (IC_50_) values of the investigated chromanone (**III**) derivatives with those of the respective carbocyclic analogs (**II**) with the same aryl substituent showed the 4-CH_3_ (**IIIb**) derivative’s outstanding potency in comparison with the respective **IIb** (the ratio of the IC_50_(**IIb**)/IC_50_(**IIIb** values is >50.0) [Table 1]. On the other hand, such an increase was not observed in the analogous 4-OCH_3_ (**IIc** and **IIIc**) derivatives [8].

The anticancer potential of chalcones is correlated with their ability to act on various molecular targets, such as ABCG2, tubulin, activated nuclear B cell growth (NF-κB), vascular endothelial growth factor (VEGF), tyrosine kinase receptor (EGFR), mesenchymal-epithelial transition factor (MET), 5-α reductase, ACP-reductase, histone deacetylase, p53, CDC25B (protein tyrosine phosphatase), retinoic acid receptors, estrogenic topoisomerase receptors, and MDM2 [5]. Several of the biological effects (e.g., NF-κB pathway inhibition (anti-inflammatory effect) [9,10], activation of the Nrf2 pathway (antitumor/cytoprotective effect) [10,11,12], inhibition of protein kinases (antitumor effect) [13,14]) of chalcones have been considered (at least in part) to be a consequence of their Michael-type reactivity toward the cysteine residues of proteins. Based on structure–activity relationship data, other chalcones with a molecular mechanism based on thiol reactivity have been reported to induce the tumor suppressor p53 [15], inhibit vascular endothelial growth factor (VEGF) [16], and inhibit cathepsin-K [17].

The reactivity of covalent inhibitors with an optimized structure is maximal with the nucleophilic amino acid residues selected on the target protein and minimal with nontargeted nucleophilic residues. Chalcones, being soft electrophiles, have an expressed preference towards the soft nucleophilic cysteine thiol residues. A common approach to achieving maximal reaction rates with the target residues is an in vitro benchmarking strategy using protein substitutes (e.g., GSH) to assess the intrinsic chemical reactivity with thiols via LC-MS/MS or NMR techniques [18]. The structural features of chalcones that affect their thiol reactivity were investigated by Amslinger and colleagues [19]. On the other hand, the three-dimensional shape of the molecules has also been reported as a determining factor in their biological activities [20]. These latter observations underline the importance of noncovalent interactions of the compounds—the requirement of the covalent interaction as well—with the target cellular macromolecules.

**Figure 2:**
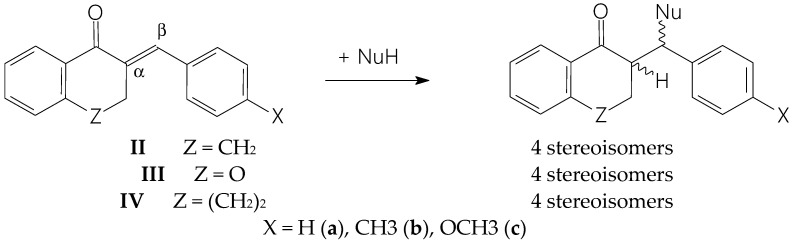
A simplified reaction scheme of the addition of GSH and NAC (NuH) to the cyclic chalcone analogs (**II**–**IV**).

The initial aim of the present study was to investigate whether the thiol reactivity of the cyclic chalcone analogs **IIIb** and **IIb** correlates with the observed differences in the cancer cell cytotoxicity of the compounds. The possible difference in the thiol reactivity of the two compounds can be considered an experimental result, which may indicate different mechanisms of action. Similar to our previous studies [21,22], reduced glutathione (GSH) and N-acetylcysteine (NAC) were used as thiol reagents. The soft nucleophilic thiols preferably react with the β-carbon atom of the enone moiety, generating a chiral carbon atom [22]. In the reaction of the cyclic chalcone analogs (**II**–**IV**), the α-carbon atom (C2) also becomes a chiral center. Due to the inherent chirality of GSH (2S,5R) and NAC (R), the reaction of the cyclic chalcone analogs can result in the formation of four diastereoisomeric adducts [1] (Figure 2).

The reactions were conducted under three different pH conditions (pH 8.0, 6.3, and 3.2) to investigate how the ratio of the protonated to unprotonated forms of the thiol function affects the reactivity of the chalcones and the stereochemical outcome of the reactions. To quantitatively characterize the progress of the reactions and formation of the thiol adducts, a composition of the chalcone–thiol incubation mixtures was analyzed at the 15, 45, 75, 105, 135, 165, 195, 225, 255, 285, and 315-min time points using HPLC-UV.

For a better understanding of the kinetic profiles of the reactions, molecular modeling calculations were performed. These analyses used methanethiol (**CH_3_SH**) and its deprotonated form (**CH_3_S^−^**) as model thiols. Computational studies modeling the bioconjugation of cysteine residues using methanethiol are frequently applied in the literature. Recently, for example, Vilarrasa et al. performed a comprehensive computational study of the addition of methanethiol to 40+ Michael acceptors as the model for bioconjugation of cysteine [23]. The mechanistic aspects of thiol additions to Michael acceptors obtained via computational methods were reviewed by Krenske et al. [24]. Recent results indicate that computational tools using simple models are already advancing the rational design of thiol acceptors in drug discovery [25].

## 2. Results

### 2.1. Reactions Under Slightly Basic (pH 8.0) Conditions

Initially, the reactions of the three chromanones (**IIIa**–**c**) were investigated under basic (pH 8.0) conditions. Considering the p*K*a values of GSH (p*K*_a_ 8.83) and NAC (p*K*_a_ 9.52) [26], 12.8% of the GSH and 2.9% of the NAC molecules are in thiolate form under these conditions. These conditions were selected to mimic the glutathione-S-transferase (GST)-catalyzed reaction, in which ionization of the GSH-thiol function increases due to the interaction of the tripeptide with the G-site of the enzyme [27].

Furthermore, cysteine is the most frequently targeted amino acid residue of proteins [28]. The p*K*a of a cysteine residue in a protein can be shifted significantly from its intrinsic p*K*a value (p*K*a about 8.6) [29]. Catalytic cysteines have p*K*a’s that are as low as 2.88 [30,31], but the p*K*a’s of noncatalytic cysteines generally range from 7.4 to 9.1 [32]. Thus, the reactivity of the investigated 3-arylidene-4-chromanones (**IIIa**–**c**) under our different conditions also gives information on their covalent modification power towards thiols with three different p*K*a values.

**Figure 3:**
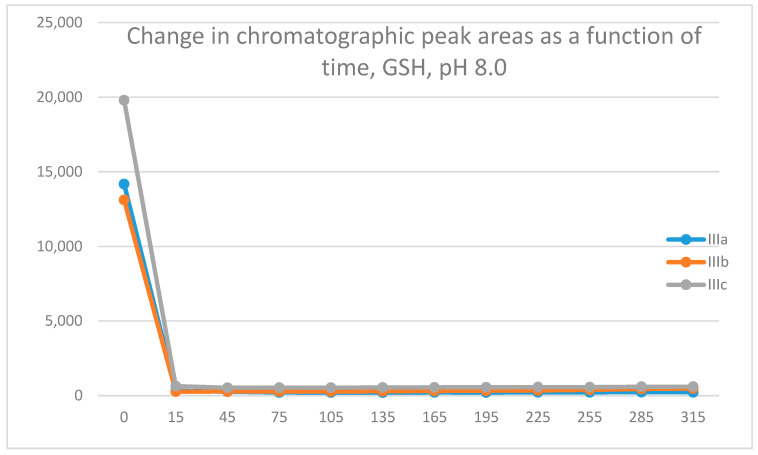
Change in the HPLC-UV chromatographic peak area of 3-arylidene-4-chromanones **IIIa**–**c** as a function of time (min) in the chalcone/GSH incubations at pH 8.0. Each data point represents the average of two independent measurements.

**Figure 4:**
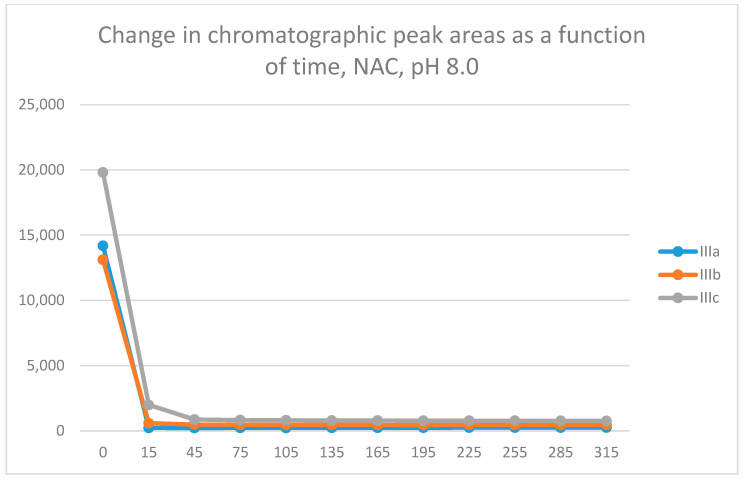
Change in the HPLC-UV chromatographic peak area of 3-arylidene-4-chromanones **IIIa**, **IIIb**, and **IIIc** as a function of time (min) in the chalcone/NAC incubations at pH 8.0. Each data point represents the average of two independent measurements.

Under such conditions, GSH (Figure 3) and NAC (Figure 4) showed unexpectedly very high intrinsic reactivity with the investigated 3-arylidene-4-chromanones (Table 2 and Table 3).

In all incubations, the peak area of the starting 3-arylidene-4-chromanones (**IIIa**–**c**) almost completely disappeared at the 45-min timepoint. A comparison of the 15-min and 45-min peak areas of the NAC-incubates showed that **IIIc** was less reactive than **IIIa** and **IIIb**. Similar differences were obtained in the comparison of the thiol reactivities of the respective tetralone derivatives **IIc** and **IIb** [21].

In a previous study, the formation of (Z)-isomers of chalcones **II** and **IV** was observed as a result of retro-Michael reaction of the formed thiol conjugates [19]. To investigate such a possibility in the present reactions, light-initiated (*E*)/(*Z*) isomerization of **IIIa**–**c** were performed [33]. The structure of the initial (*E*)- and the formed (*Z*)-isomers were identified via HPLC-MS (Figures S1–S9). HPLC-MS investigations indicated no sign of the formation of (Z)-isomers in any of the studied incubations (the isomers did not separate under the HPLC-UV conditions).

As a result of the reactions, two new chiral centers were formed in the products. Under our chromatographic conditions, however, the appearance of four separated peaks of the possibly forming four diastereomeric adducts could not be observed. In the case of the GSH-incubations, two chromatographic peaks with similar retention times were detected (Table 2) (Figures S10–S12). The structure of the adducts was supported using HPLC-MS (Figures S13–S30). The 315-min ratio of the GSH-1/GSH-2 peak areas of **IIIa**, **IIIb**, and **IIIc** was 0.83, 0.50, and 0.71, respectively. The ratio of the two peaks did not change as a function of time (Figures S31 and S32).

Analysis of the NAC-incubations showed only one chromatographic peak in the **IIIa** and **IIIb** incubates. Two chromatographic peaks could only be observed for **IIIc** (Figures S33–S35). The structure of the formed NAC-adducts was supported using HPLC-MS (Figures S36–S44). The 315-min ratio of the **IIIc** NAC-1/NAC-2 (0.81) peaks is similar to those of the GSH-1/GSH-2 (0.71) (Table 2 and Table 3). The ratio of the **IIIc** NAC-2/NAC-1 peak areas slightly increased over the investigated period (Figures S45 and S46).

### 2.2. Reactions Under Slightly Acidic (pH 6.3) Conditions

Reactions under slightly acidic conditions mimic the cellular milieu of the cancer cells [34]. Under such conditions, about 0.3% of the GSH molecules and 0.06% of the NAC molecules exist in the more reactive thiolate form. In accordance with our expectations, the progress of the reactions under such conditions was slower than that observed at pH 8.0.

**Figure 5:**
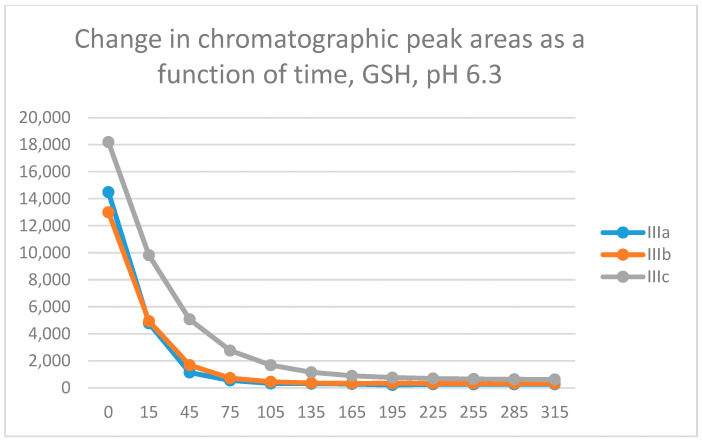
Change in the HPLC-UV chromatographic peak area of 3-arylidene-4-chromanones **IIIa**–**c** as a function of time (min) in the chalcone/GSH incubations at pH 6.3. Each data point represents the average of two independent measurements.

**Figure 6:**
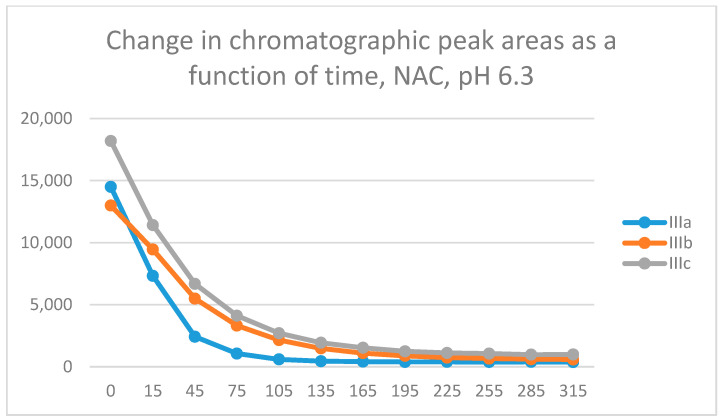
Change in the HPLC-UV chromatographic peak area of 3-arylidene-4-chromanones **IIIa**–**c** as a function of time (min) in the chalcone/NAC incubations at pH 6.3. Each data point represents the average of two independent measurements.

In the GSH incubations, the reaction of compounds **IIIa** and **IIIb** at the 105-min and **IIIc** at the 255-min timepoint reached the equilibrium state. Reactivity of the methoxy-substituted derivative (**IIIc**) was lower than that of **IIIa** and **IIIb** (Figure 5). The difference in reactivity of **IIIa**–**c** became more pronounced in the NAC-incubates. The reactivity of the substituted derivatives (**IIIb** and **IIIc**) was lower than that of the unsubstituted **IIIa** (Figure 6).

The HPLC chromatographic profile of the formed GSH- and NAC-conjugates (Figures S47–S50) was similar to those at pH 8.0 (Figures S31, S32, S45 and S46). The 315-min ratio of the GSH-1/GSH-2 peak areas of **IIIa**, **IIIb**, and **IIIc** was 0.97, 0.78, and 0.97, respectively (Table 2). The 315-min ratio of the **IIIc** NAC-1/NAC-2 peaks (0.36), however, was very different from that of the GSH-1/GSH-2 peaks (0.97) (Table 2 and Table 3).

### 2.3. Reactions Under Acidic (pH 3.2) Conditions

Under strongly acidic conditions, the thiol function of GSH and NAC is fully protonated. According to the reduced reactivity of the protonated thiol nucleophiles, the initial area of the HPLC peaks of **IIIa**–**c** was decreased by much smaller extents (Table 2 and Table 3). The progression curves of the three derivatives ran parallel in both thiol incubations. Similar to the previous results, the methoxy derivative (**IIIc**) showed the lowest reactivity under such conditions (Figures S51 and S52).

Like the above results, two chalcone-GSH and one chalcone-NAC peak could be detected in the incubates. The peak areas of the GSH-1 and GSH-2 conjugates showed a linear increase with different slopes for the three derivatives (Figures S53 and S54). The 315-min ratio of the GSH-1/GSH-2 peak areas of **IIIa**, **IIIb**, and **IIIc** was 1.3, 1.1 and 1.2, respectively. HPLC analysis of the NAC incubates showed only one adduct peak in the case of each compound (Table 3) (Figure S55).

### 2.4. Molecular Modeling Analysis

**Figure 7:**
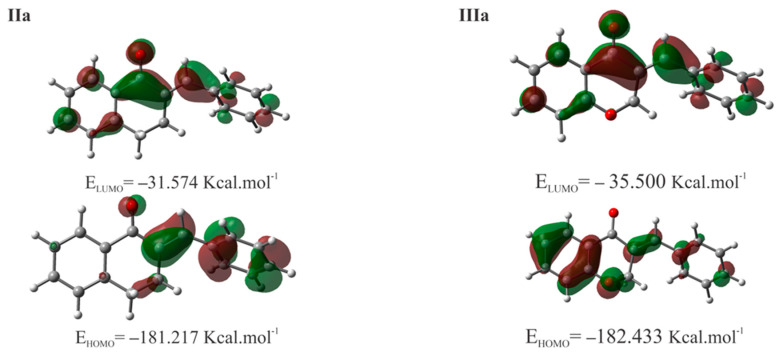
HOMO and LUMO plots of **IIa [21]** and **IIIa** calculated at the M06-2X/6-311++G(d,p) level of theory.

Table 4 shows the calculated values for the molecular properties of **Ia**, **IIa**, **IIIa**, **IVa**, methanethiol (**CH_3_SH**), and deprotonated methanethiol (**CH_3_S^−^**). The difference between the HOMO energy of **CH_3_SH** and **CH_3_S^−^** was spectacular, indicating the much higher nucleophilicity of the deprotonated thiol. The LUMO energy of the analogous carbocyclic chalcones (**Ia**, **IIa**, and **IVa**) increased in parallel with the ring size (Table 4). The HOMO and LUMO plots of compounds **IIa** and **IIIa** are shown in Figure 7. The values and coefficients of the LUMO orbital were similar in the two compounds.

According to the analysis of the condensed Fukui functions (Table 5), the orientation of the nucleophilic attack varies in response to different values of Δ*f*(r). Specifically, if Δ*f*(r) > 0, the site will likely undergo a nucleophilic attack. Conversely, if Δ*f*(r) < 0, the site is favored for an electrophilic attack.

**Figure 8:**
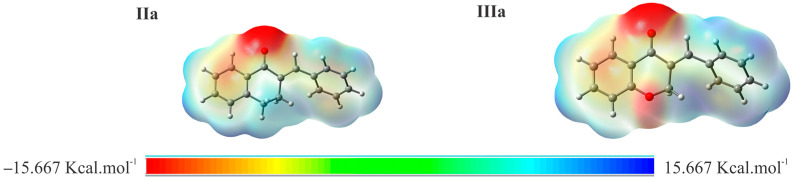
MEP surface at ρ(r) = 4.0 × 10^−4^ electrons/Bohr3 contour of the total SCF electronic density for molecules **IIa** [21] and **IIIa** at the M06-2X/6-311++G(d,p) level of theory.

To visualize the relative charge distribution on the molecular surface, molecular electrostatic potential (MEP) mapping was carried out (Figure 8). The red spots on the MEP surface represent the electron-rich sites on the surface of the molecules that are susceptible to electrophilic attack. In contrast, the blue spots represent the electron-depleted regions that are sites susceptible to nucleophilic attack. For **CH_3_S**^−^, the MEP is reddish due to the unit negative charge resulting from deprotonation.

**Figure 9:**
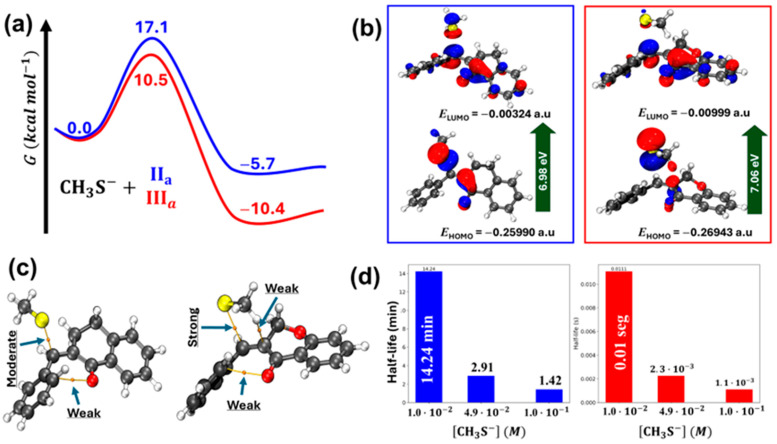
Results on the methanethiolate (**CH_3_S^−^**) addition to **IIa** and **IIIa** showing structural, energetic, and kinetic analyses of the transition states (TSs). (**a**) TS for the reaction of **CH_3_S^−^** with **IIa** and **IIIa**. Arrows indicate the strength of interactions. (**b**) Frontier orbitals (HOMO and LUMO) of TSs of **IIa** and **IIIa**. (**c**) Gibbs free energy profile for the reactions of **IIa** and **IIIa**. (**d**) Kinetic analysis showing the half-life of the reactions of **IIa** (blue bars) and **IIIa** (red bars) with different methanethiolate concentrations.

Furthermore, a series of electronic structure calculations using the density functional theory (DFT) method were performed to obtain the transition states (TSs) for the reaction of methanethiolate (**CH_3_S^−^**) with the two chalcones analogs, **IIa** and **IIIa**. For the transition state involving **IIIa**, the formation of three hydrogen bonds was observed, as identified by the critical points obtained via the quantum theory of atoms in molecules (QTAIM) methodology. The C–S bond formed in the transition state of **IIIa** exhibits a strong character; in contrast, **IIa** has a moderate one. This difference suggests a higher stability for the transition state of **IIIa**. Analyzing the distortion of the six-membered rings, it was found that in **IIIa**, the oxygen atom shifts to an envelope, while in **IIa**, **it** has a skew boat conformation (Figure 9, panel a).

Evaluating the frontier orbitals (HOMO and LUMO) of the transition states, it was found that the energy gap is approximately equal for both compounds (Figure 9, panel b), indicating that energetics is not a predominant factor in differentiating the reactivity between the compounds. On the contrary, the Gibbs free energy profile showed characteristic differences. For **IIIa**, the energy barrier is 10.5 kcal·mol^−1^, while for **IIa**, it is 17.1 kcal·mol^−1^. Thermodynamically, the product formed from **IIIa** is more stable than that formed from **IIa** with a Gibbs free energy of −10.4 kcal·mol^−1^ and 5.7 kcal·mol^−1^, respectively (Figure 9, panel c).

Using pseudo-first-order approximation, the half-lives of the two reactions were calculated. For an excess concentration of 4.9 × 10^−2^ M of methanethiolate, the estimated half-life for the reaction for **IIa** is approximately 2.91 min, while for **IIIa**, it is 2.3 × 10^−3^ s (Figure 9, panel d).

## 3. Discussion

**Figure 10:**
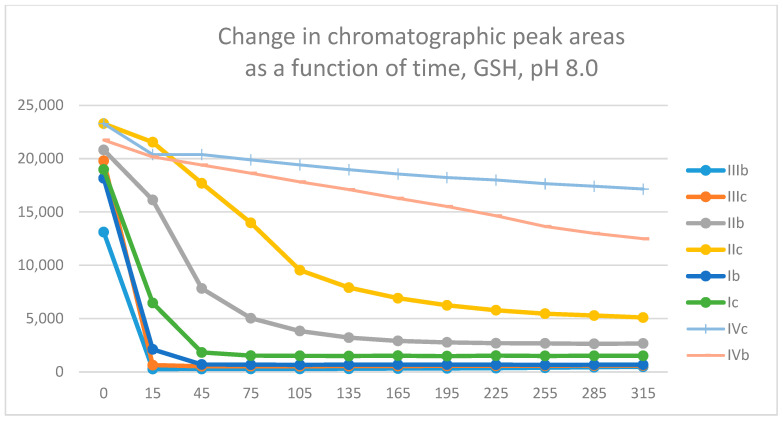
Comparison of the change in HPLC-UV chromatographic peak areas of selected chalcones (**Ib**, **Ic**) and cyclic chalcone analogs (**IIb**, **IIc**; **IIb**, **IIc**; and **IVb**, **IVc**) as a function of time (min) in the chalcone/GSH incubations at pH 8.0. Each data point represents the average of two independent measurements.

In our earlier studies, the thiol reactivity of some chalcones (**I**) [22] and the carbocyclic chalcone analogs **II** [21] and **IV** [35] were investigated. It was found that the carbocyclic chalcone analogs (**II** and **IV**) had lower thiol reactivity than the respective open-chain (**I**) ones. Figure 10 shows the progression curves of the reactions of the investigated methyl(**b**)- and methoxy(**c**)-substituted chalcones/cyclic chalcone analogs (**I**–**IV**) with GSH under basic (pH 8.0) conditions. Such a comparison shows that the seven-membered analogs (**IVc** and **IVb**) have much lower thiol reactivity than the respective open-chain (**I**) and chromanone (**III**) derivatives. Furthermore, comparing the GSH-reactivity of **IIb**,**c** with **IIIb**,**c** showed thta the chromanone analogs (**III**) have much higher reactivity than the respective carbocyclic tetralones (**II**).

Similar observations could be made by comparing the progression curves of the reactions between the above chalcones and NAC under pH 8.0 conditions. As in the case of GSH, the seven-membered cyclic analogs (**IVb** and **IVc**) showed the lowest reactivity. The reactivity of the open-chain chalcones (**Ib** and **Ic**) was comparable with that of the 3-arylidene-4-chromanones **IIIb** and **IIIc** (Figure S56). The higher thiol p*K*a value of NAC resulted in a lower reactivity and conversion rate of the carbocyclic analogs **IIb**,**c** and **IVb**,**c** compared to those of the compounds observed in their reactions with GSH (Figure 10 and Figure S56).

**Figure 11:**
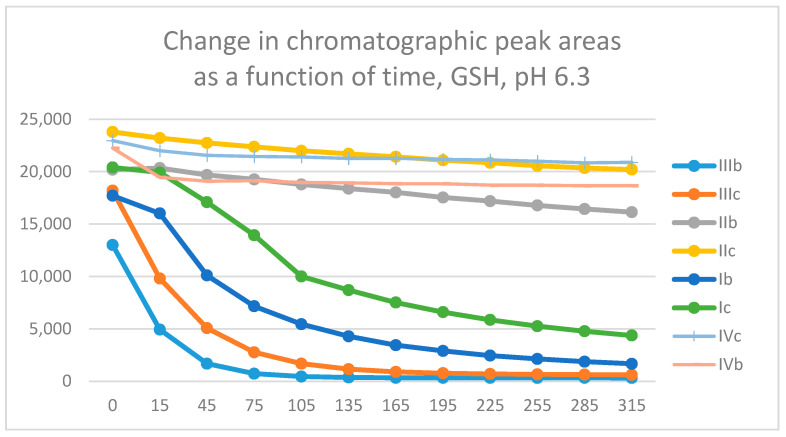
Comparison of the change in HPLC-UV chromatographic peak areas of selected chalcones (**Ib**, **Ic**) and cyclic chalcone analogs (**IIb**, **IIc**; **IIb**, **IIc**; and **IVb**, **IVc**) as a function of time (min) in the chalcone/GSH incubations at pH 6.3. Each data point represents the average of two independent measurements.

The differences observed in the reactivity of compounds **II** and **III** are more distinct in the GSH- and NAC-incubations carried out under the slightly acidic conditions (pH 6.3). Under such conditions, the chromanone derivatives (**IIIb**,**c**) showed the highest reactivity, followed by the somewhat lower one of the open-chain chalcones (**Ib**,**c**) with both thiols (Figure 11 and Figure S57). The reactivity of the two carbocyclic chalcone analogs (**II** and **IV**) was substantially lower and displayed similarity. The methyl-substituted (**b**) derivatives showed higher reactivity than the methoxy-substituted (**c**) ones in each series. The lower reactivity of the methoxy derivatives (**c**) can be explained by the more effective resonance stabilization effect of the methoxy substituent, which makes the retro-Michael reaction of the thiol-adduct intermediate more preferred [21].

Similar observations were made while investigating the GSH-reactivity of the four series (**I**–**IV**) under pH 3.2 conditions. Under such acidic conditions, the reactivity of the open-chain (**I**) and the carbocyclic (**II** and **IV**) analogs is much lower than that of the chromanones **IIIb** and **IIIc** (Figures S58 and S59).

In the thia-Michael reactions, the HOMO electron pairs of the nucleophile (**CH_3_SH** or **CH_3_S^−^**) react with the empty LUMO of the electrophile. The closer in energy these two orbitals are, the greater the energy stabilization that is obtained during the reactions [36]. In addition to the **IIa** and **IIIa**, the previously published molecular modeling parameters of the open-chain **Ia** and the seven-membered analog **IV**a have also been incorporated into Table 4. According to the calculated data, the observed thiol reactivities of the open-chain **Ia** and the carbocyclic **IIa** and **IVa** derivatives (Figure 10 and Figure 11) showed good parallelism with the increased LUMO energies of the three series. However, the LUMO energies of the parent compounds of the two more reactive series—the open chain **Ia** and the chromanone **IIIa** derivatives—are lower than that of the less reactive **IIa** and **IVa** (Table 4). Accordingly, the difference in the *E*_LUMO_ values of **IIa** and **IIIa** (−31.57 kcal/mol and −35.50 kcal/mol, respectively) can (at least partly) justify the much higher reactivity of **IIIa**.

According to the experimental results and the condensed Fukui functions of **Ia** [35], **IVa** [35], **IIa**, and **IIIa** (Table 4), the preferred site of the nucleophilic attack is the β-carbon atom (C10 and C3a in **IIIa**–**c**). The ground state relative electrophilicity of the β-carbon atoms can be reflected by their ^13^C NMR chemical shifts [37]. Table 6 summarizes the ^13^C NMR chemical shifts of the carbon atoms of the enone moiety of **Ia**, **IIa**–**c**, **IIIa**–**c**, and **IVa**. As shown, the electron densities (^13^C NMR shifts) of the C10 atoms are relatively close to each other in all the cyclic chalcone analogs (**II**, **III**, and **IV**). The only exception is the open-chain **Ia**. The reduced electron density of the beta(C10)-carbon atom, the high polarity of the C = C bond, and the conformational flexibility of the reaction intermediate can be considered to be the basis for the higher reactivity of compounds **I** compared to the C2-substituted, conformationally less flexible carbocyclic **II** and **IV** [21,35].

A comparison of the ^13^C NMR data of **IIa**–**c** with that of the respective **IIIa**–**c** [38,40] shows that the C=C bonds are more polar in the chromanone (**III**) derivatives. The increase in polarity results from the difference between the chemical shift of the C2(C3) atoms (Table 6). The observed changes indicate a significant resonance interaction between the substituents and the C2 carbon atoms. This was also the case with similar investigations of the analogous open-chain chalcones (**I**) and the (*E*)-2-(4-X-benzylidene)-1-benzosuberones (**IV**) [37].

Since the above parameters did not provide enough convincing data to explain the increased reactivity of the 4-chromanone derivatives (**III**) compared to the carbocyclic analogs (**II**), molecular modeling calculations were performed to compare the stereochemistry, relative energy, and molecular orbital characteristics of the transition states of **IIa** and **IIIa** with the deprotonated form (**CH_3_S^−^**) of the model thiol, **CH_3_SH**.

The electronic structure calculations revealed a clear difference in the stability of the transition states between chalcones **IIa** and **IIIa**. The presence of the oxygen atom in **IIIa** facilitates the formation of a hydrogen–pi interaction between the electron-deficient methyl hydrogen and the enone fragment’s pi system, resulting in a stabilized transition state. Specific parameters qualifying this type of interaction have been extensively discussed in a previous publication [41]. Analysis of the ring distortion reinforces this observation, where the envelope conformation of **IIIa** is more favored than the skew boat conformation of **IIa**.

The similarity in the energy gap of the frontier (HOMO and LUMO) orbitals between the TSs of the two compounds (**IIa** and **IIIa**) also suggests that other factors, such as intermolecular interactions and geometric stability, are more decisive in the observed reactivities. The significant difference in Gibbs free energy barriers between **IIa** and **IIIa** indicates an easier and more efficient reaction for **IIIa.**

Additionally, the kinetic analysis reveals drastically different half-lives, with **IIIa** reacting much faster than **IIa**. These results are consistent with the lower energy barrier and higher stability of the final product in the case of **IIIa**. In conclusion, the incorporation of the oxygen into the carbocyclic chalcone analogs significantly improves the stability and reactivity of the transition state and the final product. The kinetic results obtained here agree with the experimental results on the thia-Michael addition to α,β-unsaturated ketones used as fluorescent probes for imaging in single cells [42].

In the HPLC-UV chromatograms of the GSH incubations of **IIIa**–**c**, two separated GSH-chalcone peaks can be seen under all investigated conditions (Table 2). The ratio of the GSH-1/GSH-2 peaks (A_GSH-1_/A_GSH-2_) is close to unity in all pH 3.2 and pH 6.3 incubations. The ratio of the two peaks did not change over the 315-min incubation times. However, when the incubations were performed under basic conditions (pH 8.0), the incubates showed different excesses of the least polar (GSH-2) diastereomers. The 315-min A_GSH-1_/A_GSH-2_ ratio of the **IIIa**, **IIIb**, and **IIIc** was 0.83, 0.50 and 0.71, respectively (Table 2). The ratios did not change over the time of incubation. It is worth mentioning that the similar reaction of the carbocyclic **IIc** resulted in the formation of about twofold (2.2) excess of the more polar (GSH-1) diastereomers. The respective A_GSH-1_/A_GSH-2_ ratios at the pH 6.3 and 3.2 incubations were 2.3 and 3.3, respectively (Table 2).

A similar comparison of the respective NAC-incubations of **IIc** and **IIIc** showed both compounds to form only one diastereomeric peak in the pH 3.2 incubations. Under the basic (pH 8.0) conditions, the A_NAC-1_/A_NAC-2_ ratio of **IIc** and **IIIc** was 1.58 and 0.81, respectively (Table 3). Although the stereochemistry (and the chromatographic polarity) of the adducts separated as two chromatographic peaks are not known, the different diastereomeric ratios support the above-described difference in the structure of the transition state in the two (**II** and **III**) series.

## 4. Materials and Methods

### 4.1. Chemicals and Reagents

L-glutathione and N-acetyl L-cysteine were obtained from Sigma Aldrich (Budapest, Hungary). Methanol HPLC was obtained from Honeywell (Honeywell, Hungary). Trifluoroacetic acid was obtained through VWR (Budapest, Hungary). Formic acid was obtained at Fischer Chemicals. Compounds **IIIa**–**c** were synthesized as previously described [9]. For example, the synthesis of **IIIc** is described in the Supplementary Materials. Their stereochemistry was verified via ^1^H, ^13^C NMR, and X-ray studies [29]. The purity of the samples was checked using TLC and the melting point measurement. Authentic (*E*)-**IIIa**–**c** were illuminated by scattered laboratory light to obtain the mixture of respective (*E*) and (Z)-isomers [33,43]. The structure of the (*E*)- and (*Z*)-**IIIa**–**c** isomers was verified using HPLC-MS (Figures S1–S8).

### 4.2. Preparation of Solutions

Solutions of reduced glutathione and N-acetylcysteine with different pH values (pH 3.2, 6.3, and 8.0) were prepared as follows. The GSH and NAC solutions were prepared in distilled water to a total volume of 1.5 cm^3^ with a concentration of 2.0 × 10^−1^ mol.L^−1^ (0.3 mmol GSH). The pH values were set using 1M NaOH solution. Chalcone solutions were prepared freshly before incubation to a 4.6 volume using HPLC-grade methanol (4.6 cm^3^ of 6.5 × 10^−3^ mol.L^−1^, 0.03 mmol chalcone).

The NAC and GSH solutions were mixed with the chalcone solution to a final volume of 6.1 cm^3^. The solution was then kept in the dark during preparation and analysis in a temperature-controlled (37 °C) water bath for 315 min. To monitor the progress of the reactions, samples were taken at time points 15, 45, 75, 105, 135, 165, 195, 225, 255, 285, and 315 min and analyzed via HPLC-UV. Each piece of experimental data is the average of two parallel measurements. Other than the 15-min and 45-min data points, the deviation from the average was less than 5%. The deviation of the 15-min and 45-min data points of the pH 8.0 and pH 6.3 incubations varied between 10% and 20%.

### 4.3. RP-HPLC-UV Measurements

The measurements were performed using an Agilent 1100 HPLC system with a UV-Vis detector (Agilent Technologies, Waldbronn, Germany) operating at 260 nm. The components were separated using a Zobrax Eclipse XBD-C8 (150 mm × 4.6 mm, particle size 5 µm) column (Agilent Technologies, Waldbronn, Germany). The injection volume was 10 µL. Data were recorded and evaluated using Agilent Chem Station (B.03.01). The gradient elution was performed at the flow rate of 1.2 mL/min; the mobile phase consisted of (A) water and 0.1% trifluoroacetic acid and (B) methanol and 0.1% trifluoroacetic acid. The gradient profile was as follows: an isocratic period of 8 min of 40% mobile phase B, followed by a linear increase to 60% for 4 min, a second linear gradient to 90% for 3 min, and a 5-min isocratic period of 90%. The column was then equilibrated to its initial conditions with a 2-min linear gradient to 40%, followed by a 3-min isocratic period.

### 4.4. HPLC-MS Measurements

The measurements were performed using an HPLC Ultimate 3000 coupled with a mass spectrometer Q Exactive Focus (Dionex, Sunnyvale, CA, USA). The HPLC separation was performed on an Accucore RP-MS column (150 mm × 2.1 mm, particle size 2.6 µm) using an Accucore C18 defender guard precolumn (150 mm × 2.1 mm, particle size 2.6 µm). The injection volume was 5 µL; the flow rate was 0.4 mL/min. Data analyses and evaluations were performed using Thermo Scientific TranceFinder version 4.1.191.0.

A binary gradient of eluents comprised the mobile phases: (A) water and 0.1% formic acid, and (B) methanol and 0.1% formic acid. The gradient elution was as follows: 1 min of isocratic elution of 10% eluent B, followed by a linear increase to 95% until 14 min B, followed by an isocratic period of 3 min at 95% B. Eluent B then was decreased to 10% in 0.1 min, and the column was reequilibrated at 10% eluent B for 2.9 min. The sampler temperature was at room temperature, and the column oven was at 40 °C. A diode array detector (DAD) was also performed at a wavelength of 260 nm, along with the MS analysis.

The Q Exactive Focus mass spectrometer was operated with an Orbitrap mass analyzer and HESI (Heated Electrospray Ionization). The mass spectrometer parameters were constant during the measurement and were set to sheath gas (nitrogen gas) at 30 A.U. and auxiliary gas (nitrogen gas) at 10 A.U. The probe heater was set to 300 °C, and the capillary temperature was set to 350 °C. The spray voltage (+) was 3500 V, and the S lens R.F. level was 50%. Mass spectrometry specifications followed the ionization method: HESI±, having 35,000 resolution at 200 *m*/*z* and a scan range of 100–1300 amu.

### 4.5. Molecular Modeling

Theoretical calculations were performed using DFT, as implemented in the G16 software package [44]. Molecular structures were optimized using the M06-2 × 2 hybrid exchange and correlation functional with long-range correction, combined with the 6-311++G(d,p) basis set [45]. Frontier molecular orbital energies were calculated via DFT methods [46]. Molecular electrostatic potential maps were constructed to provide a visual representation of the electrostatic potential on the surface of a molecule, which can reveal regions of high and low electron density [47]. The electrostatic potential V(r) [48] at point r is defined as

(1) $$V\left(r\right)=\sum_{\alpha }\frac{Z_{A}}{\left|r_{\alpha }-r_{A}\right|}-\int \frac{\rho (r)}{r_{\alpha }-r}dr$$

where Z*a* is the charge of nuclei *a* at point **r***a* and $\rho \left(r\right)$ is the charge density at point **r**.

The local electrophilicity indices of the molecules were determined by applying the Fukui function [49,50], which can be used to predict the molecular reactive sites. The Fukui function is mathematically expressed as

(2) $$f\left(r\right)={\left[\frac{\partial \rho \left(r\right)}{\partial N}\right]}_{v},$$

where N is the number of electrons in the system, and the constant term v in the partial derivative is external potential.

The electronic structure properties of the reactants (**IIa**, **IIIa**, and **CH_3_S^−^**), products, and transition states were calculated at the M06HF/6-31G+(d) level using solvation model density (SMD). The SMD model has been widely used to simulate the aqueous environment in elucidating the mechanisms of organic reactions and is computationally less demanding than other continuum models [51,52,53].

The stationary points were characterized by analytic harmonic frequency calculations. The absence or presence of one imaginary frequency characterizes the optimized structures as local minima or transition states, respectively. The zero-point vibrational energy contributions were considered when calculating the energy barrier.

The topological analysis [54,55] was performed in terms of electron density (ρ), the Laplacian of electron density (∇2ρ), Lagrangian kinetic energy density [G(r)], potential energy density [V(r)] and energy density [E(r)] at the critical points (CPs) to efficiently describe H-bonding and its concept without borders. The criterion for evaluating the strength of the hydrogen bonds by topological analysis is described in [41] and the references therein. The condensed Fukui indices were calculated using the atomic Hirshfeld charges obtained from the population analysis. The Multiwfn package program was used to study the topological and Fukui functions [56].

The reaction rate constant was calculated using formulations based on the thermodynamical representation of the Transition State Theory [57]:

(3) $$k(T)=\frac{k_{B}T}{h}e^{-\frac{∆G^{‡}}{RT}}$$

where h is the Planck’s constant, $k_{B}$ is the Boltzmann constant, R is the universal gas constant, T is the absolute temperature, and $∆G^{‡}$ is the activation of Gibbs free energy.

From the data of the reaction rate constant of chalcone analogs (**IIa** and **IIIa**) with the methanethiol anion obtained via (1), it is possible to calculate the half-life time using pseudo-first-order approximation [52] to the excess of methanethiol:

$$t_{1/2}=ln2/(k\times \left[CH_{3}S^{-}\right])$$

where $\left[CH_{3}S^{-}\right]$ is the concentration of methanethiolate anion in the aqueous media.

The half-life of the reaction was studied at 298 K, and $\left[CH_{3}S^{-}\right]$ was in the range of 10^−2^–10^−1^ mol L^−1^, including the experimental concentration used in this work, 4.9 × 10^−2^ mol L^−1^.

## 5. Conclusions

This study revealed that the more cancer cell cytotoxic 3-(4-X-benzylidene)-4-chromanones (**IIIb** and **IIIc**) display an unexpectedly higher thiol reactivity than their carbocyclic counterparts (**IIb** and **IIc**). This increased thiol reactivity was attributed to the LUMO energies and the different topography of the molecules. Using methanethiolate anion (**CH_3_S^−^**) as a model thiol reagent, molecular modeling results indicated that the transition state of **IIIa** is hydrogen-bond stabilized, and that the adduct of **IIIa** is thermodynamically more stable than that of **IIa** (Figure 9, panel c). Furthermore, the comparison of the initial reactivities and the LUMO energies of the open chain chalcone (**Ia**) and its cyclic analogs (**II**–**IVa**), with **Ia** and **IIIa** having the more negative LUMO energies, showed higher reactivities [Table 4]. Although the interaction of the methanethiolate hydrogen and the enone carbon–carbon double bond (Figure 9, panel a) is not proof of such interaction with the respective methylene hydrogens of GSH and NAC, it cannot be excluded.

Although no (Z)-isomers could be detected in the incubation mixtures, the occurrence of retro-Michael reactions cannot be excluded. Taking into consideration the determining factors of the reverse process [58], the formation of the resonance-stabilized methoxy-substituted derivatives can be considered a determining factor for the higher reactivity of the methyl-substituted analogs in each series under each investigated condition (Figure 10, Figure 11, Figures S51 and S52).

Since the cancer cell cytotoxicity of **IIIb** is much higher than that of the respective **IIb** (IC_50_(**IIb**)/IC_50_ (**IIIb** > 50.0), it is reasonable to suppose that the observed difference in cytotoxicity of the two compounds is, at least in part, related to the much higher thiol reactivity of **IIIb**. On the other hand, such an increase was not observed in the analogous 4-OCH_3_ (**IIc** and **IIIc**) derivatives. The difference in the thiol reactivity of these latter compounds is not reflected in their IC_50_ values. The respective IC_50_ values of the two 4-OCH_3_-derivatives (**IIc** and **IIIc**) fall into the same order of magnitude (Table 1). This observation is in accordance with earlier results demonstrating the importance of the methoxy substitution of the chalcones’ ring B in developing non-covalent interactions with different proteins [16].

Based on the thiol reactivity–cancer cell cytotoxicity relationships observed so far, it seems to be a rational extension of our present and earlier [21,22,35] studies to investigate similar relationships among the respective five-membered analogs of **IIa**–**c** and sulfur analogs of **IIIa**–**c**. Such studies would also provide further data on the structure–thiol reactivity relationships of cyclic chalcone analogs. Furthermore, the results would provide additional data to firmly tune the thiol reactivity of new cyclic chalcone derivatives with targeted covalent/noncovalent modification-based biological effects.

## Figures and Tables

**Table 1:** IC_50_ (µM) data of selected (*E*)-2-(4′-X-benzylidene)-1-tetralones (**II**) [6] and (*E*)-3-(4′-benzylidene)-4-chromanones (**III**) [8].

Compound	Molt 4/8	CEM-T	L12010
**IIb**	>500	460	161
**IIc**	9.41	8.84	44
**IIIb**	8.21	8.30	71.4
**IIIc**	13.8	25.5	67.3

**Table 2:** Retention times (t_R_) ^1^ and integrated peak areas (A) of the investigated 3-arylidene-4-chromanones (**IIIa**, **IIIb**, and **IIIc**) and their GSH adducts ^2^.

pH ^3^	Compound	t_R_ (*E*)-Isomer	Area Ratio ^4^ A_315_/A_0_	t_R_ (*Z*)-Isomer	t_R_^2^ GSH-1	Area GSH-1	t_R_^2^ GSH-2	Area GSH-2
3.2	**IIIa**	16.6	0.36	N.D. ^5^	12.9	5346	13.2	4021
3.2	**IIIb**	17.1	0.42	N.D. ^5^	14.6	3748	14.8	3472
3.2	**IIIc**	16.6	0.44	N.D. ^5^	12.9	2136	13.4	1712
6.3	**IIIa**	16.6	0.02	N.D. ^5^	12.3	12,073	12.7	12,421
6.3	**IIIb**	17.1	0.03	N.D. ^5^	14.5	8028	14.7	10,248
6.3	**IIIc**	16.6	0.03	N.D. ^5^	13.0	9064	13.5	9315
8.0	**IIIa**	16.6	0.02	N.D. ^5^	12.3	10,330	12.7	12,509
8.0	**IIIb**	17.0	0.03	N.D. ^5^	14.4	5820	14.5	11,685
8.0	**IIIc**	16.6	0.03	N.D. ^5^	13.2	7615	13.6	10,692

^1^ Retention times in minutes; ^2^ Data refers to the average of two independent HPLC-UV measurements at the 315 min time point; ^3^ pH value of the aqueous thiol solution; ^4^ Ratios of peak areas measured at 0 and 315 min; ^5^ Not detectable.

**Table 3:** Retention times (t_R_) ^1^ and integrated peak areas (A) of the investigated 3-arylidene-4-chromanones (**IIIa**, **IIIb**, and **IIIc**) and their NAC adducts ^2^.

pH ^3^	Compound	t_R_ (*E*)-Isomer	Area Ratio ^4^ A_315_/A_0_	t_R_^2^ (*Z*)-Isomer	t_R_ NAC-1	Area ^2^ NAC-1	t_R_ NAC-2	Area ^2^ NAC-2
3.2	**IIIa**	16.7	0.262	N.D. ^5^	15.1	9483	N.D.	N.D.
3.2	**IIIb**	17.2	0.267	N.D. ^5^	15.9	6333	N.D.	N.D.
3.2	**IIIc**	16.7	0.315	N.D. ^5^	15.2	8088	N.D.	N.D.
6.3	**IIIa**	16.7	0.025	N.D. ^5^	15.2	10,347	N.D.	N.D.
6.3	**IIIb**	17.1	0.044	N.D. ^5^	15.9	18,353	N.D.	N.D.
6.3	**IIIc**	16.7	0.054	N.D. ^5^	15.0	3987	15.2	11,003
8.0	**IIIa**	16.6	0.017	N.D. ^5^	15.0	20,271	N.D.	N.D.
8.0	**IIIb**	17.1	0.035	N.D. ^5^	15.9	19,590	N.D.	N.D.
8.0	**IIIc**	16.7	0.038	N.D. ^5^	15.1	8099	15.2	10,006

^1^ Retention times in minutes; ^2^ Data refers to the average of two independent HPLC-UV measurements at the 315 min time point; ^3^ pH value of the aqueous thiol solution; ^4^ Ratios of peak areas measured at 0 and 315 min; ^5^ Not detectable.

**Table 4:** Reactivity indices were obtained for **Ia** [35], **IIa** [21], **IIIa**, **IVa**, **CH_3_SH** [35], and **CH_3_S^−^** [35] at the M06-2X/6-311++G(d,p) level of theory.

Descriptor	Ia kcal·mol^−1^	IIa kcal·mol^−1^	IIIa kcal·mol^−1^	IVa [35] kcal·mol^−1^	CH_3_SH kcal·mol^−1^	CH_3_S^−^ kcal·mol^−1^
E_HOMO_	−183.24	−181.22	−182.43	−180.38	−183.24	−173.45
E_LUMO_	−35.98	−31.57	−35.50	−28.44	−2.98	77.80
ΔE_HOMO-LUMO_	147.26	149.65	153.99	151.94	180.26	251.27
Chemical Potential (μ)	−109.61	−104.83	−105.44	−104.405	−93.11	47.73
Chemical Hardness (η)	147.26	149.65	153.99	151.94	−180.26	−251.27
Electrophilicity Index (ω)	40.79	35.96	36.10	35.87	−24.05	4.53

**Table 5:** Condensed Fukui function for **IIa** and **IIIa.** (For numbering, see Figure 1).

		IIa				IIIa	
Atom	*f*−	*f*+	Δ*f*	Atom	*f*−	*f*+	Δ*f*
C1	0.011	0.098	0.087	C4	0.004	0.093	0.089
O(=C)	0.053	0.114	0.060	O(=C)	0.039	0.111	0.072
C7	0.029	0.060	0.032	C3	0.013	0.038	0.025
C10	0.062	0.082	0.020	C3a	0.043	0.086	0.043

**Table 6:** ^13^C NMR chemical shifts (ppm; in CDCl_3_) of the carbon atoms of the enone moiety of **Ia**, **IIa**–**c**, **IIIa**–**c**, and **IVa**.

Compound [Reference]	δC1 *(C4 **)	δC2 *(C3 **)	δC10 *(C3a **)	Δ(δ(C10 */C3a **)-δ(C2 */C3 **))
**Ia** [37]	190.5	122.1	144.8	22.78
**IIa** [37]	187.9	135.4	136.6	1.2
**IIb** [37]	187.9	134.7	136.8	2.1
**IIc** [37]	187.8	133.5	136.6	3.1
**IIIa** [38]	182.4	131.1	136.1	5.0
**IIIa** [39]	181.3	131.6	136.9	5.3
**IIIb** [40]	182.2	130.1	137.5	7.4
**IIIc** [40]	182.1	128.9	137.2	8.3
**IVa** [37]	198.0	137.8	138.0	0.2

* Numbering refers to compounds **I**, **II**, and **IV**. ** Numbering refers to compounds **III**. (See Figure 1).

## Data Availability

Data are contained within the article and Supplementary Materials.

## Supplementary Materials

The following supporting information can be downloaded at https://www.mdpi.com/article/10.3390/molecules29235493/s1, Synthesis of 3-(4-methoxybenzylidene)-2,3-dihydro-1-benzopyran-4-one (**IIIc**). **Figure S1.** High resolution, positive mode HPLC-MS chromatogram of **IIIa** illuminated by scattered laboratory light. (t_r_ 12.52 min: (*Z*)-**IIIa**; t_r_ 12.82 min: IIIa ((*E*)-**IIIa**)). (Extracted ion chromatogram of *m*/*z* 237.0914 [(**IIIa**) + H]^+^). **Figure S2**. High resolution, positive mode HESI MS spectrum of (*Z*)-**IIIa**. (*m*/*z* 237.0914 [(**IIIa**) + H]^+^ and *m*/*z* 259.0733 [(**IIIa**) + Na]^+^ t_r_ 12.52 min). **Figure S3**. High resolution, positive mode HESI MS spectrum of (*E*)-**IIIa**. (*m*/*z* 237.0915 [(**IIIa**) + H]^+^ t_r_ 12.82 min). **Figure S4**. High resolution, positive mode HPLC-MS chromatogram of **IIIb** illuminated by scattered laboratory light. (t_r_ 13.29 min: (*Z*)-**IIIb**; t_r_ 13.52 min: **IIIb** ((*E*)-**IIIb**)). (Extracted ion chromatogram of *m*/*z* 251.1072 [(**IIIb**) + H]^+^). **Figure S5**. High resolution, positive mode HESI MS spectrum of (*Z*)-**IIIb**. (*m*/*z* 251.1070 [(**IIIb**) + H]^+^ and *m*/*z* 273.0889 [(**IIIb**) + Na]^+^ t_r_ 13.28 min). **Figure S6**. High resolution, positive mode HESI MS spectrum of (*E*)-**IIIb**. (*m*/*z* 251.1072 [(**IIIb**) + H]^+^ t_r_ 13.52 min). **Figure S7**. High resolution, positive mode HPLC-MS chromatogram of **IIIc** illuminated by scattered laboratory light. (t_r_ 12.72 min: (*Z*)-**IIIc**; t_r_ 12.84 min: **IIIc** ((*E*)-**IIIc**)). (Extracted ion chromatogram of *m*/*z* 267.1021 [(***IIIc***) + H]^+^). **Figure S8**. High resolution, positive mode HESI MS spectrum of (*Z*)-**IIIc**. (*m*/*z* 267.1021 [(**IIIc**) + H]^+^ and *m*/*z* 289.0840 [(**IIIc**) + Na]^+^ t_r_ 12.71 min). **Figure S9**. High resolution, positive mode HESI MS spectrum of (*E*)-**IIIc**. (*m*/*z* 267.1021 [(**IIIc**) + H]^+^ t_r_ 12.84 min). **Figure S10**. HPLC-UV chromatogram of the **IIIa**/GSH incubate (pH 6.3; 15 min sample). (**IIIa**: t_r_16.62 min, **IIIa-GSH-1** conjugate: t_r_ 12.86 min, **IIIa-GSH-2** conjugate: t_r_ 13.17 min.) **Figure S11**. HPLC-UV chromatogram of the **IIIb**/GSH incubate (pH 6.3; 15 min sample). (**IIIb**: t_r_ 17.09 min, **IIIb-GSH-1** conjugate: t_r_ 14.58 min, **IIIb-GSH-2** conjugate: t_r_ 14.77 min.). **Figure S12**. HPLC-UV chromatogram of the **IIIc**/GSH incubate (pH 6.3; 15 min sample). (**IIIc**: t_r_16.65 min, **IIIc-GSH-1** conjugate: t_r_ 13.11 min, **IIIc-GSH-2** conjugate: t_r_ 13.51 min.). **Figure S13**. High resolution, positive mode HPLC-MS chromatograms of the **IIIa/GSH** incubate (pH 8.0 sample). **Figure S14**. High resolution, positive mode HESI MS spectrum of the **IIIa** (t_r_ 12.83 min) in the sample of pH 8.0 incubate, (237.0916 [(**IIIa**) + H]^+^). **Figure S15**. High resolution, positive mode HESI MS spectrum of the **IIIa-GSH**-**1** conjugate (t_r_ 9.34 min) formed in the sample of the pH 8.0 incubate, (*m*/*z* 544.1762 [(**IIIa*-*GSH**) + H]^+^). **Figure S16**. High resolution, negative mode HESI MS spectrum of the **IIIa-GSH**-**1** conjugate (t_r_ 9.34 min) formed in the sample of the pH 8.0 incubate, (*m*/*z* 542.1617 [(**IIIa*-*GSH**) − H]^−^), *m*/*z* 306.0771 [**GSH** − H]^−^) and *m*/*z* 1085.3296 [(**IIIa**-**GSH**)_2_ − H]^−^). **Figure S17**. High resolution, positive mode HESI MS spectrum of the **IIIa-GSH**-**2** conjugate (t_r_ 9.47 min) formed in the sample of the pH 8.0 incubate, (*m*/*z* 544.1761 [(**IIIa*-*GSH**) + H]^+^). **Figure S18**. High resolution, negative mode HESI MS spectrum of the **IIIa-GSH**-**2** conjugate (t_r_ 9.48 min) formed in the sample of the pH 8.0 incubate, (*m*/*z* 542.1617 [(**IIIa*-*GSH**) − H]^−^, *m*/*z* 306.0772 [**GSH** − H]^−^ and *m*/*z* 1085.3297 [(**IIIa-GSH**)_2_ − H]^−^). **Figure S19**. High resolution, positive mode HPLC-MS chromatograms of the **IIIb/GSH** incubate (pH 8.0 sample). **Figure S20**. High resolution, positive mode HESI MS spectrum of the **IIIb** (t_r_ 13.52 min) in the sample of the pH 8.0 incubate, (*m*/*z* 251.1072 [(**IIIb**) + H]^+^ and *m*/*z* 273.0891 [(**IIIb**) + Na]^+^). **Figure S21**. High resolution, positive mode HESI MS spectrum of the **IIIb-GSH**-**1** conjugate (t_r_ 10.23 min) formed in the sample of the pH 8.0 incubate, (*m*/*z* 558.1918 [(**IIIb**-**GSH**) + H]^+^). **Figure S22**. High resolution, negative mode HESI MS spectrum of the **IIIb-GSH**-**1** conjugate (t_r_ 10.23 min) formed in the sample of the pH 8.0 incubate, (*m*/*z* 556.1772 [(**IIIb-GSH**) − H]^−^, *m*/*z* 306.0771 [**GSH** − H]^−^ and *m*/*z* 1113.3611 [(**IIIb-GSH**)_2_ − H]^−^). **Figure S23**. High resolution, positive mode HESI MS spectrum of the **IIIb-GSH**-**2** conjugate (t_r_ 10.37 min) formed in the sample of the pH 8.0 incubate, (*m*/*z* 558.1920 [(**IIIb-GSH**) + H]^+^). **Figure S24**. High resolution, negative mode HESI MS spectrum of the **IIIb-GSH**-**2** conjugate (t_r_ 10.38 min) formed in the sample of the pH 8.0 incubate, (*m*/*z* 556.1772 [(**IIIb*-*GSH**) − H]^−^, *m*/*z* 306.0771 [**GSH**-H]^−^ and *m*/*z* 1113.3612 [(**IIIb-GSH**)_2_ − H]^−^). **Figure S25.** High resolution, positive mode HPLC-MS chromatograms of the **IIIc-GSH** incubate (pH 8.0 sample). **Figure S26**. High resolution, positive mode HESI MS spectrum of the **IIIc** (t_r_ 12.85 min) in the sample of the pH 8.0 incubate, (*m*/*z* 267.1021 [(**IIIc**) + H]^+^ and *m*/*z* 289.0840 [(**IIIc**) + Na]^+^). **Figure S27**. High resolution, positive mode HESI MS spectrum of the **IIIc-GSH**-**1** conjugate (t_r_ 9.43 min) formed in the sample of the pH 8.0 incubate, (*m*/*z* 574.1871 [(**IIIc-GSH**) + H]^+^). **Figure S28**. High resolution, negative mode HESI MS spectrum of the **IIIc-GSH 1** conjugate (t_r_ 9.44 min) formed in the sample of the pH 8.0 incubate, (*m*/*z* 572.1724 [(**IIIc-GSH**) − H]^−^, *m*/*z* 306.0772 [**GSH** − H]^−^ and *m*/*z* 1145.3516 [(**IIIc-GSH**)_2_ − H]^−^). **Figure S29**. High resolution, positive mode HESI MS spectrum of the **IIIc-GSH**-**2** conjugate (t_r_ 9.61 min) formed in the sample of the pH 8.0 incubate, (*m*/*z* 574.1871 [(**IIIc-GSH**) + H]^+^). **Figure S30**. High resolution, negative mode HESI MS spectrum of the **IIIc-GSH**-**2** conjugate (t_r_ 9.62 min) formed in the sample of the pH 8.0 incubate, (*m*/*z* 572.1724 [(**IIIc*-*GSH**) − H]^−^, *m*/*z* 306.0772 [**GSH** − H]^−^ and *m*/*z* 1145.3513 [(**IIIc-GSH**)_2_ − H]^−^). **Figure S31**. Change in the HPLC-UV chromatographic peak area of adduct-1 of **IIIa**, **IIIb**, and **IIIc** as a function of time (min) in the chalcone/GSH incubations at pH 8.0. **Figure S32**. Change in the HPLC-UV chromatographic peak area of adduct-2 of **IIIa**, **IIIb**, and **IIIc** as a function of time (min) in the chalcone/GSH incubations at pH 8.0. **Figure S33**. HPLC-UV chromatogram of the **IIIa**/NAC incubate (pH 6.3; 15 min sample). (**IIIa**: t_r_16.69 min, **IIIa-NAC-1** conjugate: t_r_ 15.18 min.) **Figure S34.** HPLC-UV chromatogram of the **IIIb**/NAC incubate (pH 6.3; 15 min sample). (**IIIb**: t_r_17.03 min, **IIIb-NAC-1** conjugate: t_r_ 15.83 min.) **Figure S35**. HPLC-UV chromatogram of the **IIIc**/NAC incubate (pH 6.3; 15 min sample). (**IIIc**: t_r_16.67 min, **IIIc-NAC-1** conjugate: t_r_ 14.98 min, **IIIc-NAC-2** conjugate: t_r_ 15.14 min.) **Figure S36**. High resolution, HPLC-MS chromatograms of **IIIa**/**NAC** incubate (pH 8.0 sample). **Figure S37**. High resolution, negative mode HESI MS spectrum of the **IIIa-NAC-1** conjugate (t_r_ 10.82 min) formed in the sample of the pH 8.0 incubate. (*m*/*z* 398.1072 [(**IIIa-NAC**) − H]^−^, *m*/*z* 797.2227 [(**IIIa-NAC**)_2_ − H]^−^, *m*/*z* 819.2043 [(**IIIa-NAC**)_2_ + Na − 2H]^−^). **Figure S38**. High resolution, negative mode HESI MS spectrum of the **IIIa-NAC-2** conjugate (t_r_ 10.89 min) formed in the sample of the pH 8.0 incubate. (*m*/*z* 398.1073 [(**IIIa-NAC**) − H]^−^, *m*/*z* 797.2227 [(**IIIa-NAC**)_2_ − H]^−^, *m*/*z* 819.2043 [(**IIIa-NAC**)_2_ + Na − 2H]^−^). **Figure S39**. High resolution, HPLC-MS chromatograms of **IIIb**/**NAC** incubate (pH 8.0 sample). **Figure S40**. High resolution, negative mode HESI MS spectrum of the **IIIb-NAC-1** conjugate (t_r_ 11.58 min) formed in the sample of the pH 8.0 incubate. (*m*/*z* 412.1232 [(**IIIb-NAC**) − H]^−^, *m*/*z* 825.2541 [(**IIIb-NAC**)_2_ − H]^−^, *m*/*z* 847.2354 [(**IIIb-NAC**)_2_ + Na − 2H]^−^). **Figure S41**. High resolution, negative mode HESI MS spectrum of the **IIIb-NAC-2** conjugate (t_r_ 11.70 min) formed in the sample of the pH 8.0 incubate. (*m*/*z* 412.1232 [(**IIIb-NAC**) − H]^−^, *m*/*z* 825.2541 [(**IIIb-NAC**)_2_ − H]^−^, *m*/*z* 847.2354 [(**IIIb-NAC**)_2_ + Na − 2H]^−^). **Figure S42**. High resolution, HPLC-MS chromatograms of **IIIc**/**NAC** incubate (pH 8.0 sample). **Figure S43**. High resolution, negative mode HESI MS spectrum of the **IIIc-NAC-1** conjugate (t_r_ 10.84 min) formed in the sample of the pH 8.0 incubate. (*m*/*z* 428.1179 [(**IIIc-NAC**) − H]^−^, *m*/*z* 857.2437 [(**IIIc-NAC**)_2_ − H]^−^, *m*/*z* 879.2251 [(**IIIc-NAC**)_2_ + Na − 2H]^−^). **Figure S44**. High resolution, negative mode HESI MS spectrum of the **IIIc-NAC-2** conjugate (t_r_ 10.95 min) formed in the sample of the pH 8.0 incubate. (*m*/*z* 428.1179 [(**IIIc-NAC**) − H]^−^, *m*/*z* 857.2437 [(**IIIc-NAC**)_2_ − H]^−^, *m*/*z* 879.2251 [(**IIIc-NAC**)_2_ + Na − 2H]^−^). **Figure S45**. Change in the HPLC-UVchromatographic peak area of adduct-1 of **IIIa**, **IIIb**, and **IIIc** as a function of time (min) in the chalcone/NAC incubations at pH 8.0. **Figure S46**. Change in the HPLC-UV chromatographic peak area of adduct-2 of **IIIc** as a function of time (min) in the chalcone/NAC incubations at pH 8.0. Each data point represents the average of two independent measurements. **Figure S47**. Change in the HPLC-UV chromatographic peak area of adduct-1 of **IIIa**, **IIIb**, and **IIIc** as a function of time (min) in the chalcone/GSH incubations at pH 6.3. Each data point represents the average of two independent measurements. **Figure S48**. Change in the HPLC-UV chromatographic peak area of adduct-2 of **IIIa**, **IIIb**, and **IIIc** as a function of time (min) in the chalcone/GSH incubations at pH 6.3. Each data point represents the average of two independent measurements. **Figure S49**. Change in the HPLC-UV chromatographic peak area of adduct-1 of **IIIa**, **IIIb**, and **IIIc** as a function of time (min) in the chalcone/NAC incubations at pH 6.3. Each data point represents the average of two independent measurements. **Figure S50**. Change in the HPLC-UV chromatographic peak area of adduct 1 of **IIIa**, **IIIb**, and **IIIc** as a function of time (min) in the chalcone/NAC incubations at pH 6.3. Each data point represents the average of two independent measurements. **Figure S51.** Change in the HPLC-UV chromatographic peak area of chalcones **IIIa**–**c** as a function of time (min) in the chalcone/GSH incubations at pH 3.2. Each data point represents the average of two independent measurements. **Figure S52.** Change in the HPLC-UV chromatographic peak area of chalcones **IIIa**–**c** as a function of time (min) in the chalcone/NAC incubations at pH 3.2. Each data point represents the average of two independent measurements. **Figure S53**. Change in the HPLC-UV chromatographic peak area of adduct-1 of **IIIa**, **IIIb**, and **IIIc** as a function of time (min) in the chalcone/GSH incubations at pH 3.2. Each data point represents the average of two independent measurements. **Figure S54**. Change in the HPLC-UV chromatographic peak area of adduct-2 of **IIIa**, **IIIb**, and **IIIc** as a function of time (min) in the chalcone/GSH incubations at pH 3.2. Each data point represents the average of two independent measurements. **Figure S55**. Change in the HPLC-UV chromatographic peak area of adduct-1 of **IIIa**, **IIIb**, and **IIIc** as a function of time (min) in the chalcone/NAC incubations at pH 3.2. Each data point represents the average of two independent measurements. **Figure S56**. Comparison of change of the HPLC-UV chromatographic peak areas of selected chalcones (**Ib**, **Ic**) and cyclic chalcone analogs (**IIb**, **IIc**; **IIb**, **IIc**; and **IVb**, **IVc**) as a function of time (min) in the chalcone/NAC incubations, pH 8.0. Each data point represents the average of two independent measurements. **Figure S57**. Comparison of change of the HPLC-UV chromatographic peak areas of selected chalcones (**Ib**, **Ic**) and cyclic chalcone analogs (**IIb**, **IIc**; **IIb**, **IIc**; and **IVb**, **IVc**) as a function of time (min) in the chalcone/NAC incubations, pH 6.3. Each data point represents the average of two independent measurements. **Figure S58**. Comparison of change of the HPLC-UV chromatographic peak areas of selected chalcones (**Ib**, **Ic**) and cyclic chalcone analogs (**IIb**, **IIc**; **IIb**, **IIc**; and **IVb**, **IVc**) as a function of time (min) in the chalcone/GSH incubations, pH 3.2. **Figure S59**. Comparison of change of the HPLC-UV chromatographic peak areas of selected chalcones (**Ib**, **Ic**) and cyclic chalcone analogs (**IIb**, **IIc**; **IIb**, **IIc**; and **IVb**, **IVc**) as a function of time (min) in the chalcone/NAC incubations, pH 3.2.

## References

[1] Rozmer Z., Perjési P. (2016). Naturally occurring chalcones and their biological activities. Phytochem. Rev..

[2] Constantinescu T., Mihis A.G. (2022). Two important anticancer mechanisms of natural and synthetic chalcones. Int. J. Mol. Sci..

[3] Rajendran G., Bhanu D., Aruchamy B., Ramani P., Pandurangan N., Bobba K.N., Oh E.J., Chung H.Y., Gangadaran P., Ahn B.-C. (2022). Chalcone: A promising bioactive scaffold in medicinal chemistry. Pharmaceuticals.

[4] Leite F.F., de Sousa N.F., de Oliveira B.H.M., Duarte G.D., Ferreira M.D.L., Scotti M.T., Filho J.M.B., Rodrigues L.C., de Moura R.O., Mendonça-Junior F.J.B. (2023). Anticancer activity of chalcones and its derivatives: Review and in silico studies. Molecules.

[5] Shalaby M.A., Rizk S.A., Fahim A.M. (2023). Synthesis, reactions and application of chalcones: A systematic review. Org. Biomol. Chem..

[6] Dimmock J.R., Kandepu N.M., Nazarali A.J., Kowalchuk T.P., Motaganahalli N., Quail J.W., Mykytiuk P.A., Audette G.F., Prasad L., Perjési P. (1999). Conformational and quantitative structure−activity relationship study of cytotoxic 2-arylidenebenzocycloalkanones. J. Med. Chem..

[7] Dimmock J.R., Zello G.A., Oloo E.O., Quail J.W., Kraatz H.-B., Perjési P., Aradi F., Takács-Novák K., Allen T.M., Santos C.L. (2002). Correlations between cytotoxicity and topography of some 2-arylidenebenzocycloalkanones determined by X-ray crystallography. J. Med. Chem..

[8] Perjési P., Das U., De Clercq E., Balzarini J., Kawase M., Sakagami H., Stables J.P., Loránd T., Rozmer Z., Dimmock J.R. (2008). Design, synthesis and antiproliferative activity of some 3-benzylidene-2,3-dihydro-1-benzopyran-4-ones which display selective toxicity for malignant cells. Eur. J. Med. Chem..

[9] Folmer F., Blasius R., Morceau F., Tabudravu J., Dicato M., Jaspars M., Diederich M. (2006). Inhibition of TNFα-induced activation of nuclear factor ΚB by Kava (*Piper methysticum*) derivatives. Biochem. Pharmacol..

[10] Laphanuwat P., Kongpetch S., Senggunprai L., Prawan A., Kukongviriyapan V. (2022). Licochalcone A induces cholangiocarcinoma cell death via suppression of Nrf2 and NF-ΚB signaling pathways. Asian Pac. J. Cancer Prev..

[11] de Freitas Silva M., Pruccoli L., Morroni F., Sita G., Seghetti F., Viegas C., Tarozzi A. (2018). The Keap1/Nrf2-ARE pathway as a pharmacological target for chalcones. Molecules.

[12] Egbujor M.C., Saha S., Buttari B., Profumo E., Saso L. (2021). Activation of Nrf2 signaling pathway by natural and synthetic chalcones: A therapeutic road map for oxidative stress. Expert Rev. Clin. Pharmacol..

[13] Zhuang C., Zhang W., Sheng C., Zhang W., Xing C., Miao Z. (2017). Chalcone: A privileged structure in medicinal chemistry. Chem. Rev..

[14] Gomes M.N., Muratov E.N., Pereira M., Peixoto J.C., Rosseto L.P., Cravo P.V.L., Andrade C.H., Neves B.J. (2017). Chalcone derivatives: Promising starting points for drug design. Molecules.

[15] Issaenko O.A., Amerik A.Y. (2012). Chalcone-based small-molecule inhibitors attenuate malignant phenotype via targeting deubiquitinating enzymes. Cell. Cycle.

[16] Mahapatra D.K., Bharti S.K., Asati V. (2015). Anti-cancer chalcones: Structural and molecular target perspectives. Eur. J. Med. Chem..

[17] Ramalho S.D., Bernades A., Demetrius G., Noda-Perez C., Vieira P.C., Yu dos Santos C., da Silva J.A., de Moraes M.O., Mousinho K.C. (2013). Synthetic chalcone derivatives as inhibitors of cathepsins K and B, and their cytotoxic evaluation. Chem. Biodivers..

[18] Jackson P.A., Widen J.C., Harki D.A., Brummond K.M. (2017). Covalent modifiers: A chemical perspective on the reactivity of α,β-unsaturated carbonyls with thiols via hetero-Michael addition reactions. J. Med. Chem..

[19] Rücker H., Al-Rifai N., Rascle A., Gottfried E., Brodziak-Jarosz L., Gerhäuser C., Dick T.P., Amslinger S. (2015). Enhancing the anti-inflammatory activity of chalcones by tuning the Michael acceptor site. Org. Biomol. Chem..

[20] Kozurkova M., Tomeckova V. (2020). Interaction of Chalcone Derivatives with Important Biomacromolecules. Chalcones and Their Synthetic Analogs.

[21] Kenari F., Pintér Z., Molnár S., Borges I.D., Camargo A.J., Napolitano H.B., Perjési P. (2024). (*E*)-2-Benzylidenecyclanones: Part XIX. Reaction of (*E*)-2-(4’-X-benzylidene)-1-tetralones with cellular thiols. Comparison of thiol reactivities of open-chain chalcones and their six- and seven-membered cyclic analogs. Int. J. Mol. Sci..

[22] Kenari F., Molnár S., Perjési P. (2021). Reaction of chalcones with cellular thiols. The effect of the 4-substitution of chalcones and protonation state of the thiols on the addition process. Diastereoselective thiol addition. Molecules.

[23] Costa A.M., Bosch L., Petit E., Vilarrasa J. (2021). Computational study of the addition of methanethiol to 40+ Michael acceptors as a model for the bioconjugation of cysteines. J. Org. Chem..

[24] Roseli R.B., Keto A.B., Krenske E.H. (2023). Mechanistic aspects of thiol additions to Michael acceptors: Insights from computations. WIREs Comput. Mol. Sci..

[25] Arafet K., Serrano-Aparicio N., Lodola A., Mulholland A.J., Gonzalez F.V., Swiderek K., Moliner V. (2021). Mechanism of inhibition of SARS-CoV-2 Mpro by N3 peptidyl Michael acceptor explained by QM/MM simulations and design of new derivatives with tunable chemical reactivity. Chem. Sci..

[26] Aldini G., Altomare A., Baron G., Vistoli G., Carini M., Borsani L., Sergio F. (2018). N-acetylcysteine as an antioxidant and disulphide breaking agent: The reasons why. Free Radic. Res..

[27] Armstrong R.N. (1997). Structure, catalytic mechanism, and evolution of the glutathione transferases. Chem. Res. Toxicol..

[28] Mehta N.V., Degani M.S. (2023). The expanding repertoire of covalent warheads for drug discovery. Drug Discov. Today.

[29] Roos G., Foloppe N., Messens J. (2013). Understanding the p*K*a of redox cysteines: The key role of hydrogen bonding. Antioxid. Redox Signal..

[30] Pinitglang S., Watts A.B., Patel M., Reid J.D., Noble M.A., Gul S., Bokth A., Naeem A., Patel H., Thomas E.W. (1997). A classical enzyme active center motif lacks catalytic competence until modulated electrostatically. Biochemistry.

[31] Zhang Z.Y., Dixon J.E. (1993). Active site labeling of the Yersinia protein tyrosine phosphatase: The determination of the p*K*a of the active site cysteine and the function of the conserved histidine 402. Biochemistry.

[32] Bulaj G., Kortemme T., Goldenberg D.P. (1998). Ionization-reactivity relationships for cysteine thiols in polypeptides. Biochemistry.

[33] Kupcewicz B., Balcerowska-Czerniak G., Małecka M., Paneth P., Krajewska U., Rozalski M. (2013). Structure–cytotoxic activity relationship of 3-arylideneflavanone and chromanone (*E*,*Z* isomers) and 3-arylflavones. Bioorg. Med. Chem. Lett..

[34] Rohani N., Hao L., Alexis M.S., Joughin B.A., Krismer K., Moufarrej M., Soltis A., Lauffenburger D., Yaffe M.B., Burge C.B. (2019). Acidification of tumor at stromal boundaries drives transcriptome alterations associated with aggressive phenotypes. Cancer Res..

[35] Kenari F., Molnár S., Borges I.D., Napolitano H.B., Perjési P. (2023). (*E*)-2-Benzylidenecyclanones: Part XVIII. Study the possible link between glutathione reactivity and cancer cell cytotoxic effects of some cyclic chalcone analogs a comparison of the reactivity of the open-chain and the seven-membered homologs. Int. J. Mol. Sci..

[36] LoPachin R.M., Gavin T., DeCaprio A., Barber D.S. (2012). Application of the hard and soft, acids and bases (HSAB) theory to toxicant–target interactions. Chem. Res. Toxicol..

[37] Perjési P., Linnanto J., Kolehmainen E., Ősz E., Virtanen E. (2005). *E*-2-Benzylidenebenzocycloalkanones. IV. Studies on transmission of substituent effects on ^13^C NMR chemical shifts of *E*-2-(X-benzylidene)-1-tetralones, and -benzosuberones. Comparison with the ^13^C NMR data of chalcones and *E*-2-(X-benzylidene)-1-indanones. J. Mol. Struct..

[38] Cheng X.-M., Huang Z.-T., Zheng Q.-Y. (2011). Topochemical photodimerization of (*E*)-3-benzylidene-4-chromanone derivatives from β-type structures directed by halogen groups. Tetrahedron.

[39] Tóth G., Simon A., Lévai A., Kahlig H., Kalchhauser H. (2001). ^17^O NMR studies on (*E*)-3-arylidenechromanone and -flavanones derivatives. Magn. Reson. Chem..

[40] Valkonen A., Laihia K., Kolehmainen E., Kauppinen R., Perjési P. (2012). Structural studies of seven homoisoflavonoids, six thiohomoisoflavonoids, and four structurally related compounds. Struct. Chem..

[41] de Almeida L.R., Carvalho P.S., Napolitano H.B., Oliveira S.S., Camargo A.J., Figueredo A.S., de Aquino G.L.B., Carvalho-Silva V.H. (2017). Contribution of directional dihydrogen interactions in the supramolecular assembly of single crystals: Quantum chemical and structural investigation of C_17_H_17_N_3_O_2_ azine. Cryst. Growth Des..

[42] Chen J., Jiang X., Carroll S.L., Huang J., Wang J. (2015). Theoretical and experimental investigation of thermodynamics and kinetics of thiol-Michael addition reactions: A case study of reversible fluorescent probes for glutathione imaging in single cells. Org. Lett..

[43] Siddaiah V., Rao C.V., Venkateswarlu S., Krishnaraju A.V., Subbarajub G.V. (2006). Synthesis, stereochemical assignments, and biological activities of homoisoflavonoids. Bioorg. Med. Chem..

[44] Frisch M., Trucks G., Schlegel H., Scuseria G., Robb M., Cheeseman J., Scalmani G., Barone V., Petersson G., Nakatsuji H. (2016). Gaussian 16 Revision C. 01. 2016.

[45] Zhao Y., Truhlar D.G. (2008). The M06 suite of density functionals for main group thermochemistry, thermochemical kinetics, non-covalent interactions, excited states, and transition elements: Two new functionals and systematic testing of four M06-class functionals and 12 other functionals. Theor. Chem. Acc..

[46] Zhang G., Musgrave C.B. (2007). Comparison of DFT methods for molecular orbital eigenvalue calculations. J. Phys. Chem. A.

[47] Weiner P.K., Langridge R., Blaney J.M., Schaefer R., Kollman P.A. (1982). Electrostatic potential molecular surfaces. Proc. Natl. Acad. Sci. USA.

[48] Náray-Szabó G., Ferenczy G.G. (1995). Molecular electrostatics. Chem. Rev..

[49] Fukui K. (1982). The role of frontier orbitals in chemical reactions (Nobel Lecture). Angew. Chem. Int. Ed. Engl..

[50] Fukui K. (1982). Role of frontier orbitals in chemical reactions. Science.

[51] Sanches-Neto F.O., Coutinho N.D., Aquilanti V., Silva W.A., Carvalho-Silva V.H. (2023). Mechanism and kinetics of the degradation of nitazoxanide and hydroxychloroquine drugs by hydroxyl radicals: Theoretical approach to ecotoxicity. J. Braz. Chem. Soc..

[52] Sanches-Neto F.O., Coutinho N.D., Palazzetti F., Carvalho-Silva V.H. (2020). Temperature dependence of rate constants for the H(D) + CH_4_ reaction in gas and aqueous phase: Deformed transition-state theory study including quantum tunneling and diffusion effects. Struct. Chem..

[53] Sanches-Neto F.O., Ramos B., Lastre-Acosta A.M., Teixeira A.C.S.C., Carvalho-Silva V.H. (2021). Aqueous picloram degradation by hydroxyl radicals: Unveiling mechanism, kinetics, and ecotoxicity through experimental and theoretical approaches. Chemosphere.

[54] Bader R.F.W. (1985). Atoms in molecules. Acc. Chem. Res..

[55] Matta C.F., Boyd R.J., Matta C.F., Boyd R.J. (2007). The Quantum Theory of Atoms in Molecules: From Solid State to DNA and Drug Design.

[56] Lu T., Chen F. (2012). Multiwfn: A multifunctional wavefunction analyzer. J. Comput. Chem..

[57] Laidler K.J., Eyring H., Glasstone S., Laidler K.J., Eyring H., Inc. McGraw-Hill Book Company (1941). The Theory of Rate Processes: The Kinetics of Chemical Reactions, Viscosity, Diffusion and Electrochemical Phenomena.

[58] Allen C.F.H., Humphlett W.J. (1966). The thermal reversibility of the Michael reaction V. The effect of the structure of certain thiol adducts on cleavage. Can. J. Chem..

