# Supplementary material for: (E)-2-Benzylidenecyclanones: Part XX—Reaction of Cyclic Chalcone Analogs with Cellular Thiols: Unexpected Increased Reactivity of 4-Chromanone- Compared to 1-Tetralone Analogs in Thia-Michael Reactions
Source: Molecules. 2024 Nov 21;29(23):5493. doi: 10.3390/molecules29235493 (PMC11643487; doi:10.3390/molecules29235493)
Supplement: Supplementary file 1 [file molecules-29-05493-s001.zip › Supplementary_Chromanones_PP_Final.pdf]

Article

(*E*)-2-Benzylidenecyclanones: Part XX. Reaction of cyclic chalcone analogs with cellular thiols. Unexpected increased reactivity of 4-chromanone- compared to 1-tetralone-analogs in thia-Michael reactions

Gábor Bognár[1], Fatemeh Kenari[1], Zoltán Pintér[1], Igor D. Borges[2], Ademir J. Camargo[2], Heibbe C. B. Oliveira[3], Flávio Olimpio Sanches-Neto[3,5], Valter H. Carvalho-Silva[4], Hamilton B. Napolitano[2] and Pál Perjési[1,2]*

[1]Institute of Pharmaceutical Chemistry, University of Pécs, H-7624, Pécs, Hungary
[2]Grupo de Química Teórica e Estrutural de Anápolis, Universidade Estadual de Goiás, Anápolis, GO, Brazil
[3]Laboratório de Estrutura Eletrônica e Dinâmica Molecular, Universidade Federal de Goiás, Goiânia, GO, Brazil
[4]Laboratory for Modeling of Physical and Chemical Transformations, Research and Graduate Center, Goiás State University, 75132-903, Anápolis, Brazil
[5]Instituto Federal de Educação, Ciência e Tecnologia de Goiás, 72876-601, Valparaíso de Goiás, GO, Brasil;
Instituto de Química, Universidade de Brasília, Caixa Postal 4478, 70904-970, Brasília, Brasil

*Correspondence: pal.perjesi@gytk.pte.hu; Tel.: +36-72-503-650

Abstract: *In vitro* relative cytotoxicity (IC_{50}(**IIb**)/IC_{50}(**IIIb**) of (*E*)-3-(4'-methylbenzylidene)-4-chromanone (**IIIb**) towards human Molt 4/C8 and CEM T-lymphocytes showed >50 increase in comparison to those of the respective tetralone derivative (**IIb**). On the other hand, such an increase was not observed in the analogous 4-OCH_3 (**IIc** and **IIIc**) derivatives. In order to study whether thiol reactivity – as a possible basis of the mechanism of action – correlates with the observed cytotoxicities, kinetics of the non-enzyme catalyzed reactions with reduced glutathione (GSH) and N-acetylcysteine (NAC) of **IIIb** and **IIIc** were investigated. Reactivity of the compounds and stereochemical outcome of the reactions were evaluated using high-pressure liquid chromatography-mass spectrometry (HPLC-MS). Molecular modeling calculations were performed to rationalize the unexpectedly higher thiol reactivity of the chromanones (**III**) compared to the carbocyclic analog tetralones (**II**). The results indicated the possible role of spontaneous thiol-reactivity of compounds **III** in their recorded biological effects.

Keywords: chalcone; homoisoflavones; benzylidenechromanones; anticancer activity; glutathione; N-acetylcysteine; thia-Michael addition; Molecular electrostatic; DFT calculations

Synthesis of 3-(4-methoxybenzylidene)-2,3-dihydro-1-benzopyran-4-one (**IIIc**)

The mixture of 1.5 mmol 4-chromanone, 1.5 mmol of 4-methoxybenzaldehyde and 7 of drops piperidine was kept at 105 ^{o}C for 1h. The reaction product was crystallized from methanol. M.p. 131–132 °C. Yield: 72%. R_f: 0.75. IR: 1665 (C=O) cm^{-1}. ^{1}H NMR (500 MHz): δ: 3.88 (s, 3H, OCH_3), 5.40 (s, 2H, OCH_2), 6.99 (q, 3H, Ar-H), 7.08 (t, 1H, Ar-H), 7.30(d, 2H, Ar-H), 7.50 (t, 1H, Ar-H), 7.86 (s, 1H, =CH), 8.02 (dd, 1H, Ar-H).

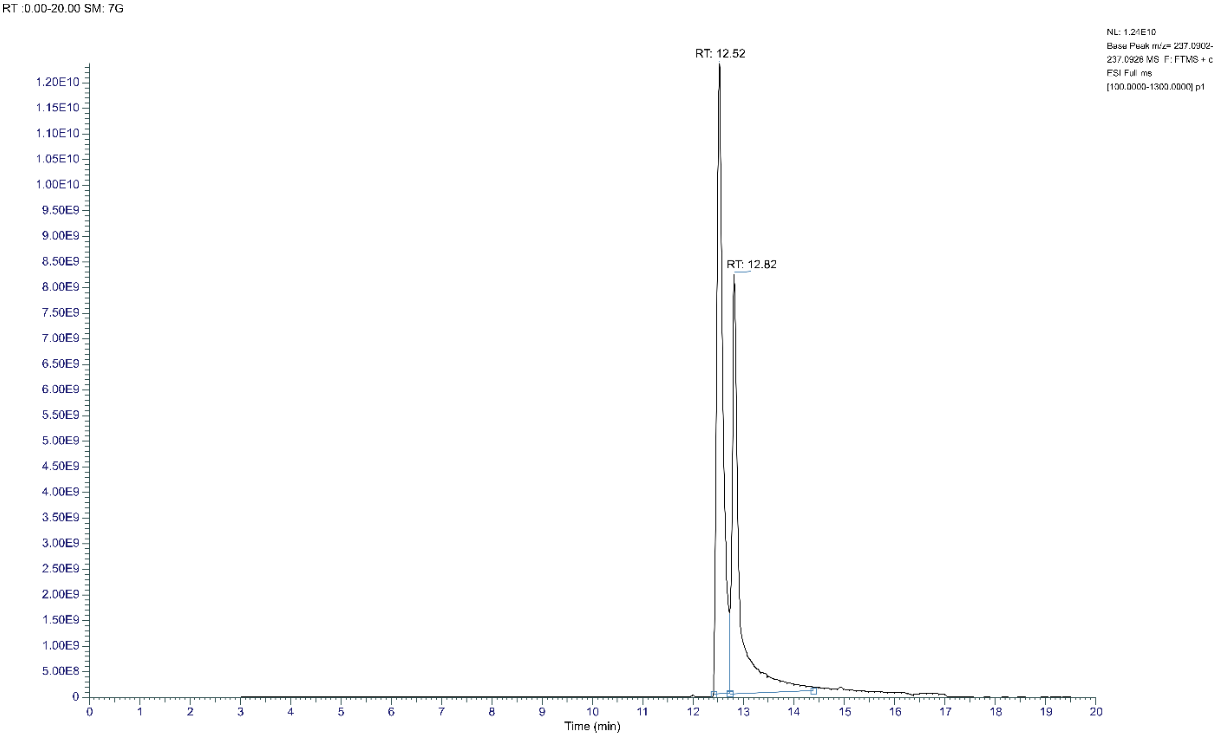

Figure S1. High resolution, positive mode HPLC-MS chromatogram of **IIIa** illuminated by scattered laboratory light. (t_r 12.52 min: (*Z*)-**IIIa**; t_r 12.82 min: **IIIa** ((*E*)-**IIIa**)). (Extracted ion chromatogram of m/z 237.0914 [(**IIIa**)+H]$^+$).

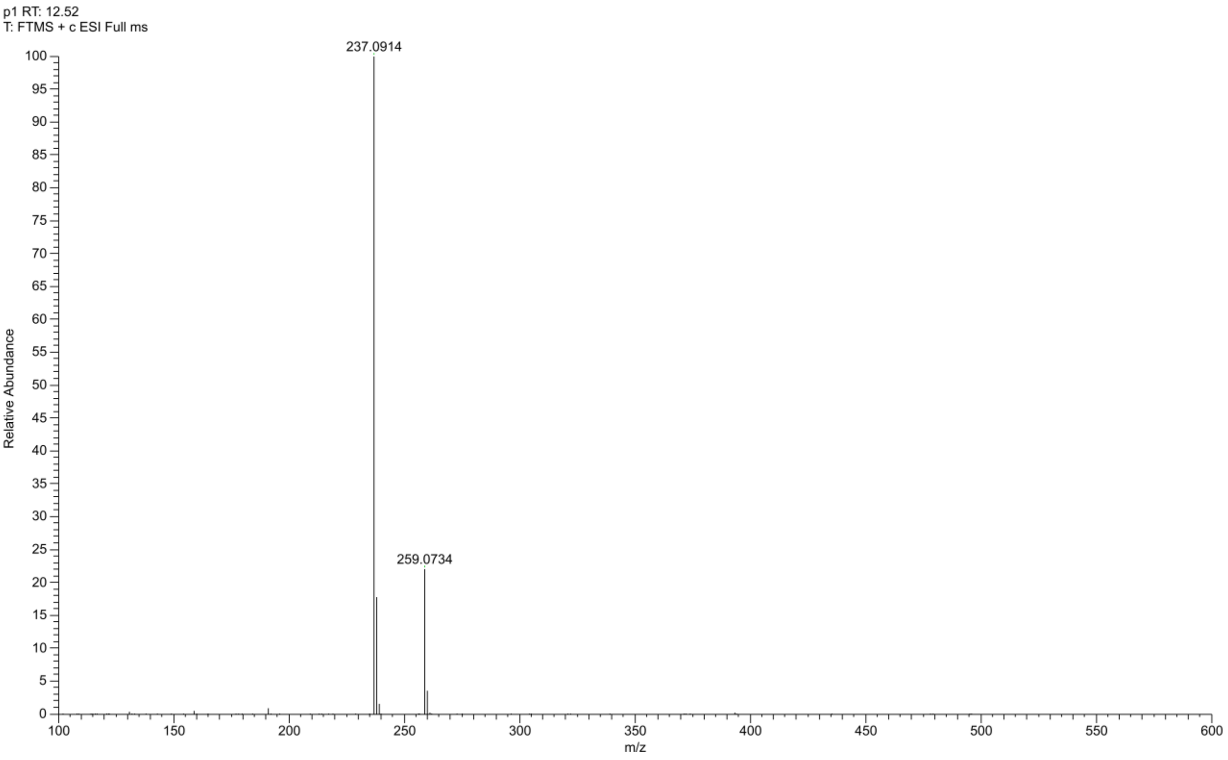

Figure S2. High resolution, positive mode HESI MS spectrum of (*Z*)-**IIIa**. (m/z 237.0914 [(**IIIa**)+H]$^+$ and m/z 259.0733 [(**IIIa**)+Na]$^+$ t_r 12.52 min).

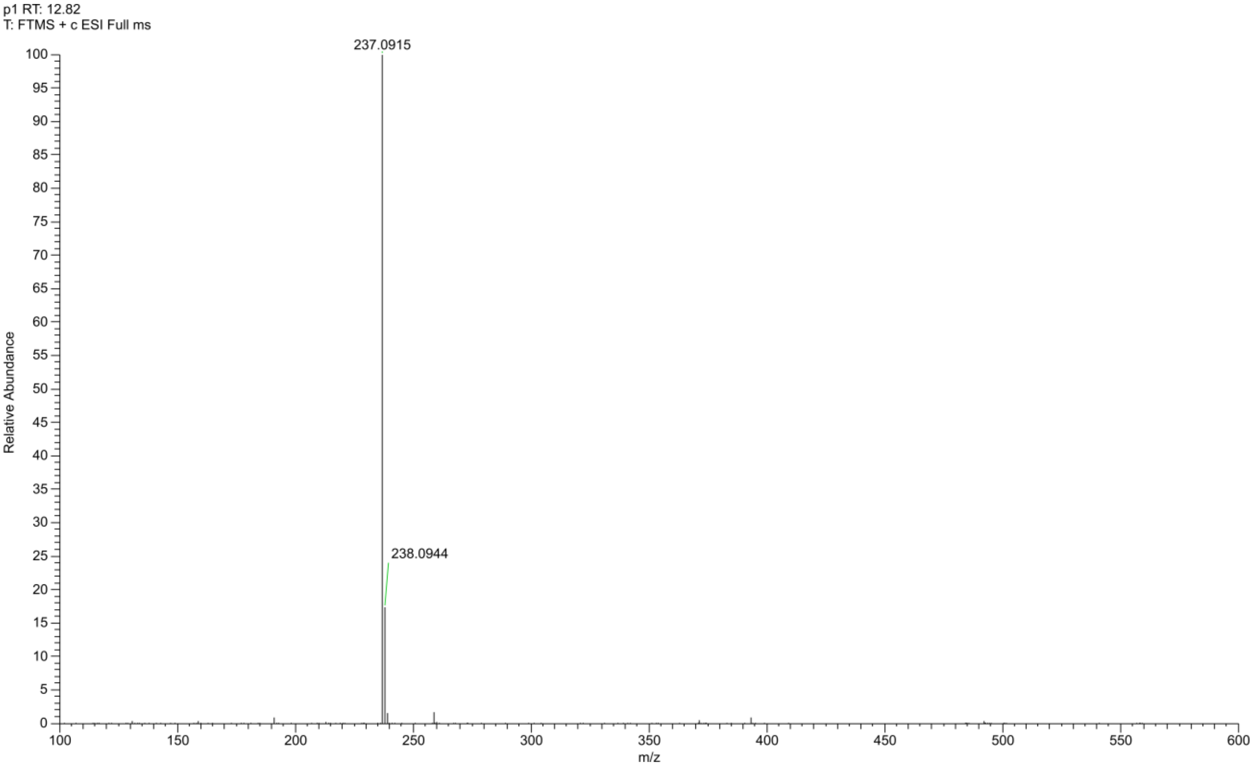

Figure S3. High resolution, positive mode HESI MS spectrum of (*E*)-**IIIa**. (m/z 237.0915 [(**IIIa**)+H]$^+$ t_r 12.82 min).

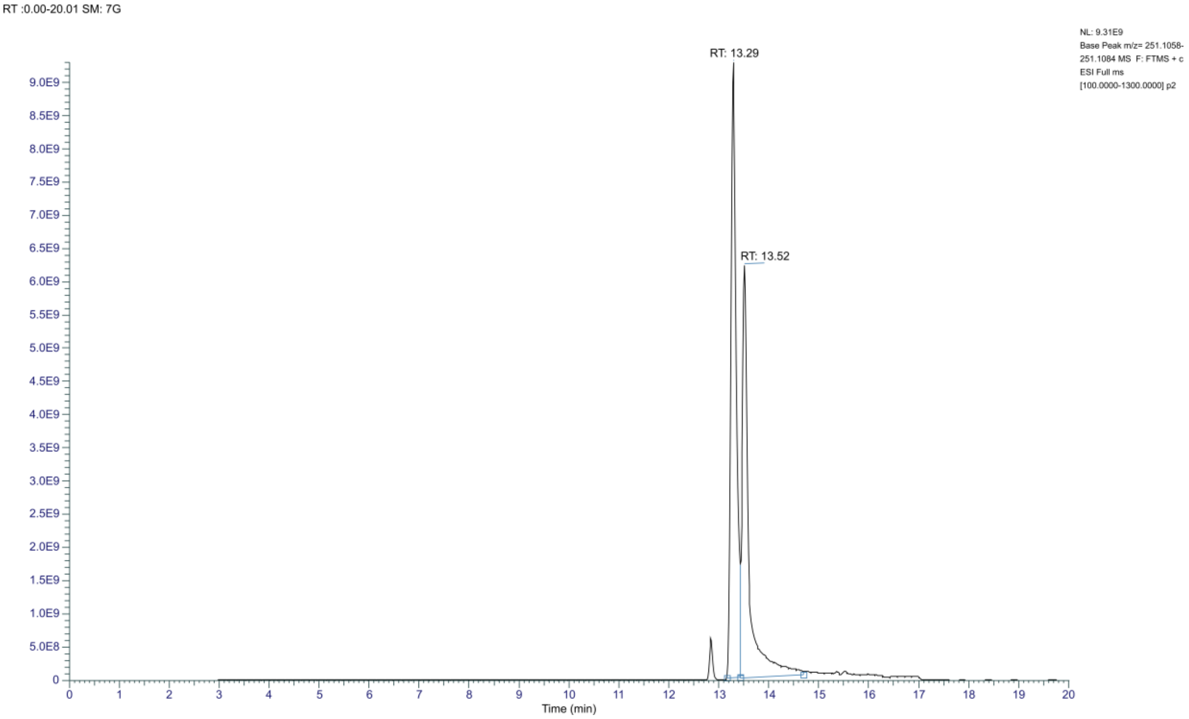

Figure S4. High resolution, positive mode HPLC-MS chromatogram of **IIIb** illuminated by scattered laboratory light. (t_r 13.29 min: (*Z*)-**IIIb**; t_r 13.52 min: **IIIb** ((*E*)-**IIIb**)). (Extracted ion chromatogram of m/z 251.1072 [(**IIIb**)+H]$^+$).

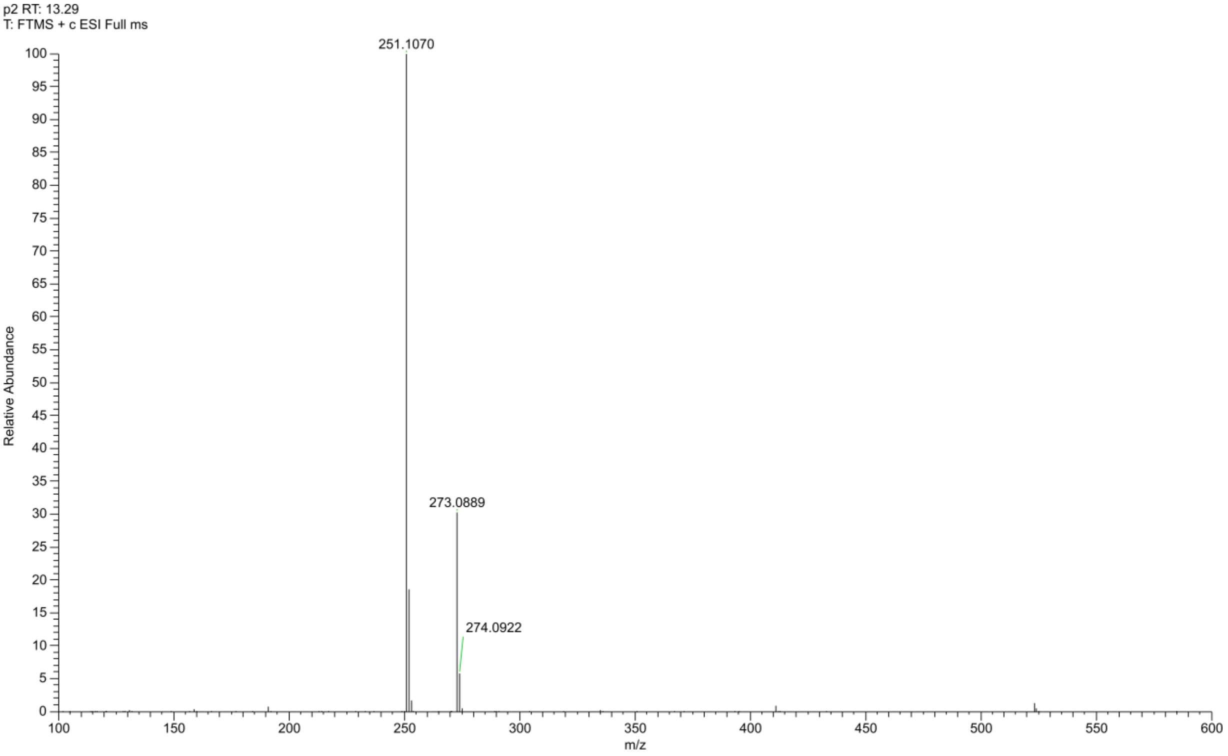

Figure S5. High resolution, positive mode HESI MS spectrum of (*Z*)-**IIIb**. (m/z 251.1070 [(**IIIb**)+H]$^+$ and m/z 273.0889 [(**IIIb**)+Na]$^+$ t_r 13.28 min).

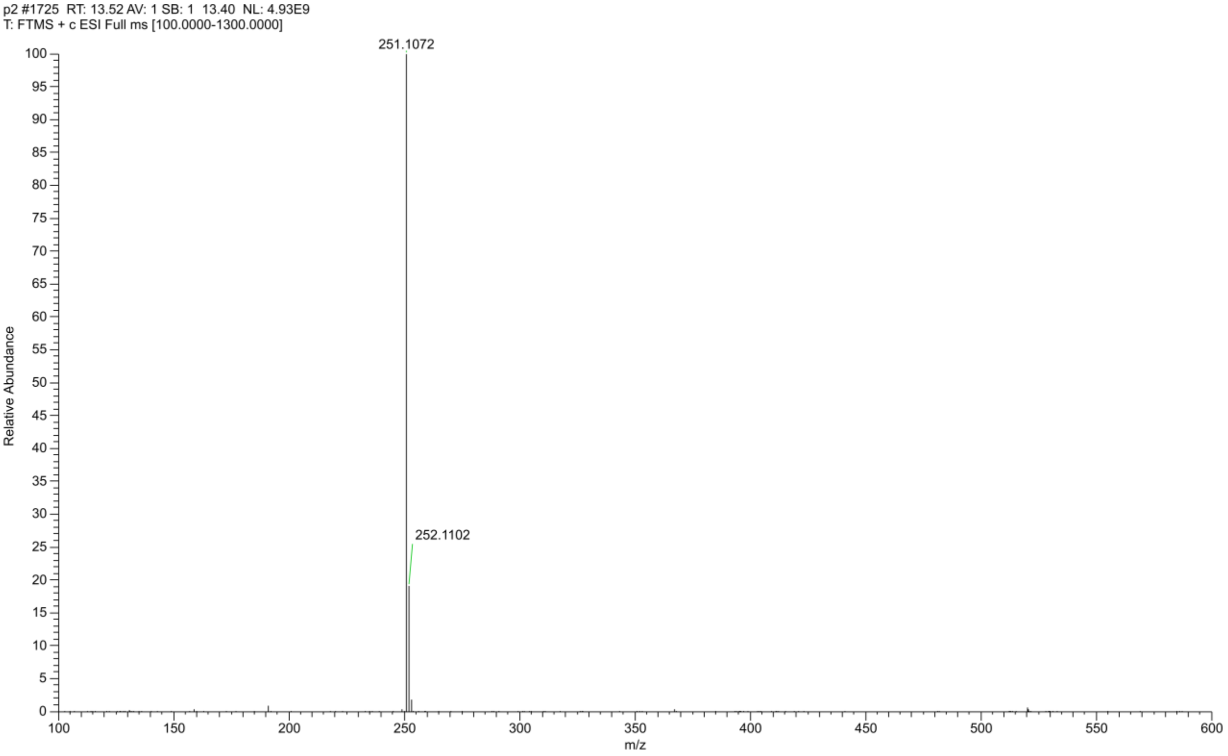

Figure S6. High resolution, positive mode HESI MS spectrum of (*E*)-**IIIb**. (m/z 251.1072 [(**IIIb**)+H]$^+$ t_r 13.52 min).

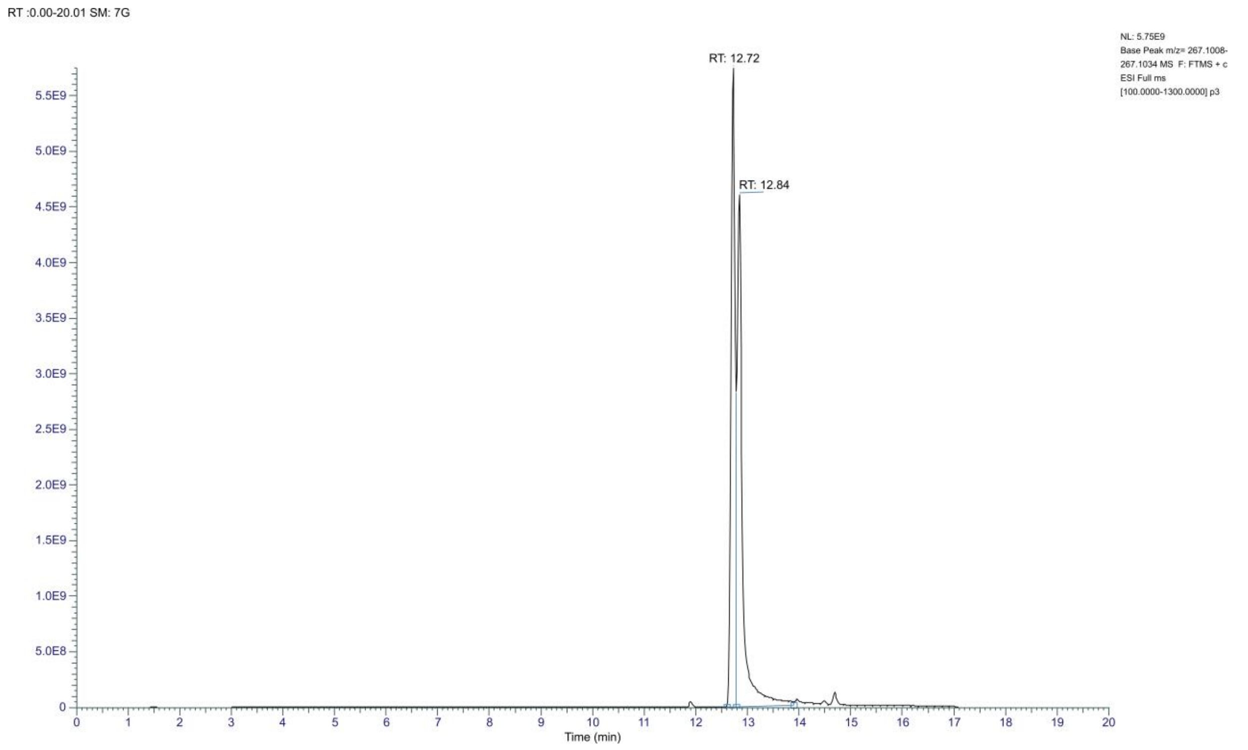

Figure S7. High resolution, positive mode HPLC-MS chromatogram of **IIIc** illuminated by scattered laboratory light. (t_r 12.72 min: (*Z*)-**IIIc**; t_r 12.84 min: **IIIc** ((*E*)-**IIIc**)). (Extracted ion chromatogram of m/z 267.1021 [(***IIIc***)+H]$^+$).

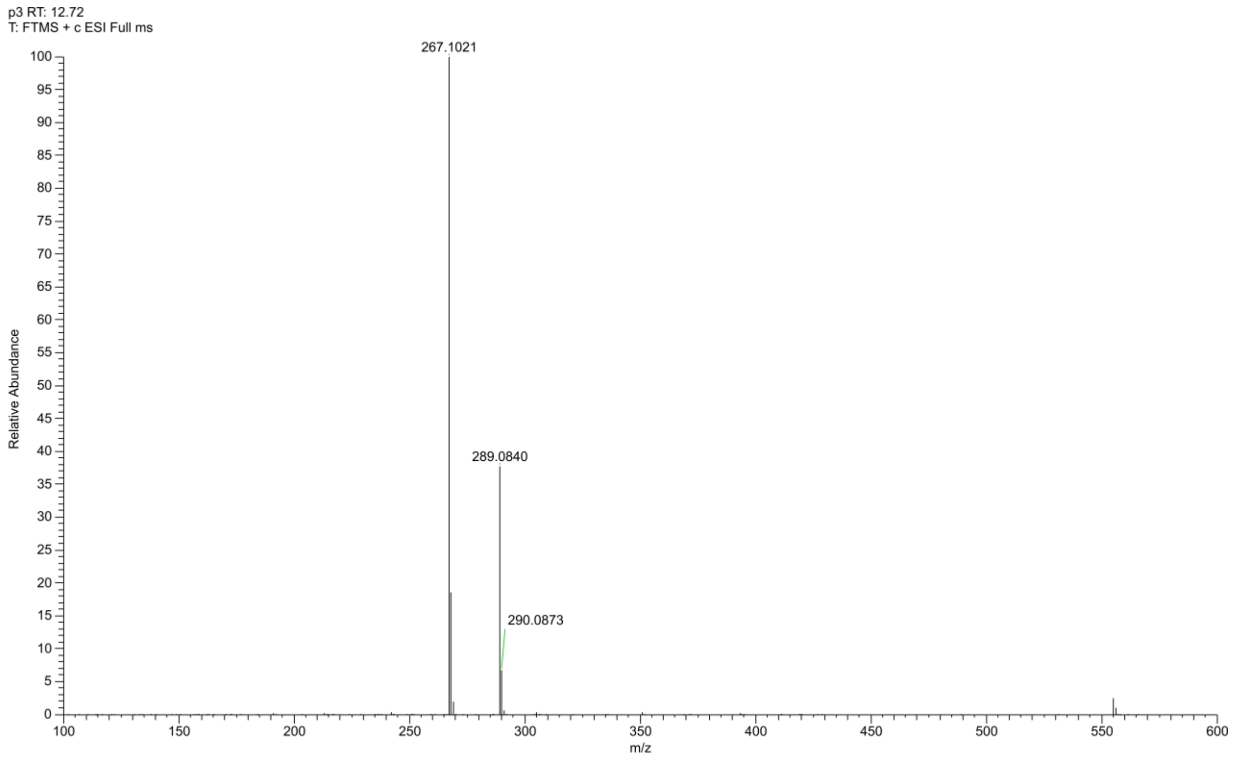

Figure S8. High resolution, positive mode HESI MS spectrum of (*Z*)-**IIIc**. (m/z 267.1021 [(**IIIc**)+H]$^+$ and m/z 289.0840 [(**IIIc**)+Na]$^+$ t_r 12.71 min).

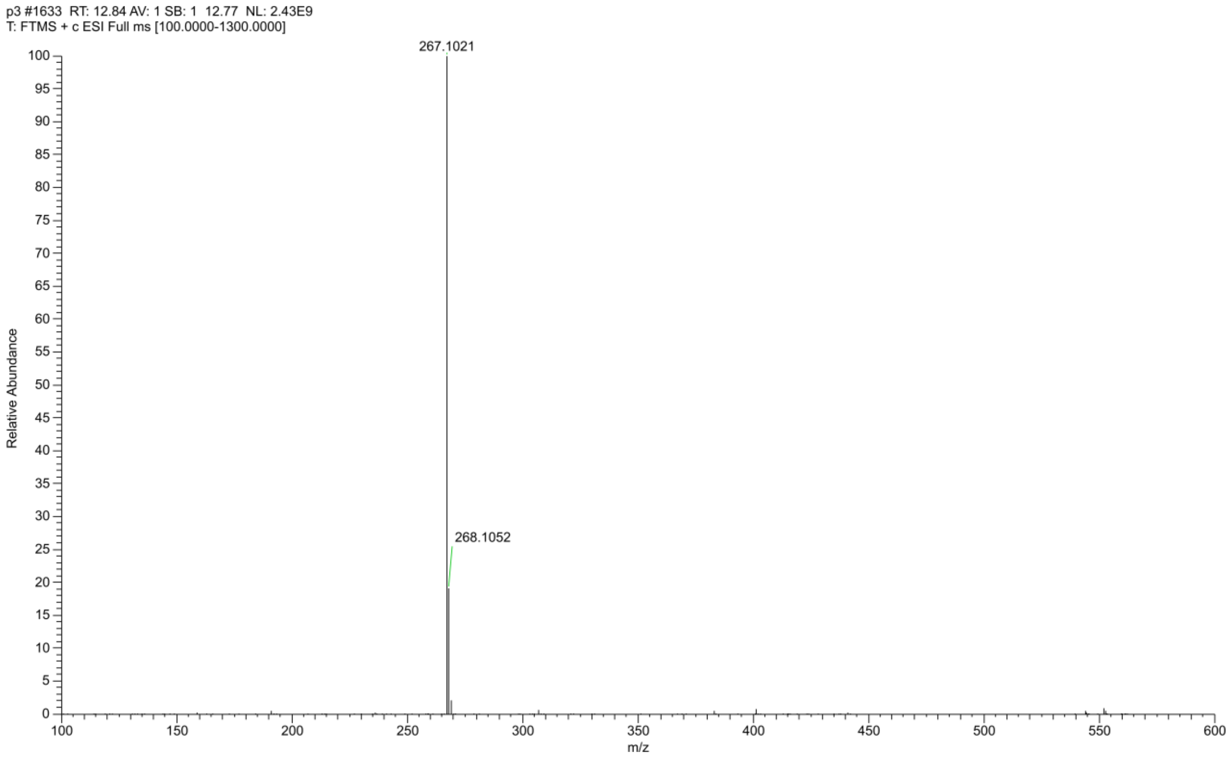

Figure S9. High resolution, positive mode HESI MS spectrum of (*E*)-**IIIc**. (m/z 267.1021 [(**IIIc**)+H]$^+$ t_r 12.84 min).

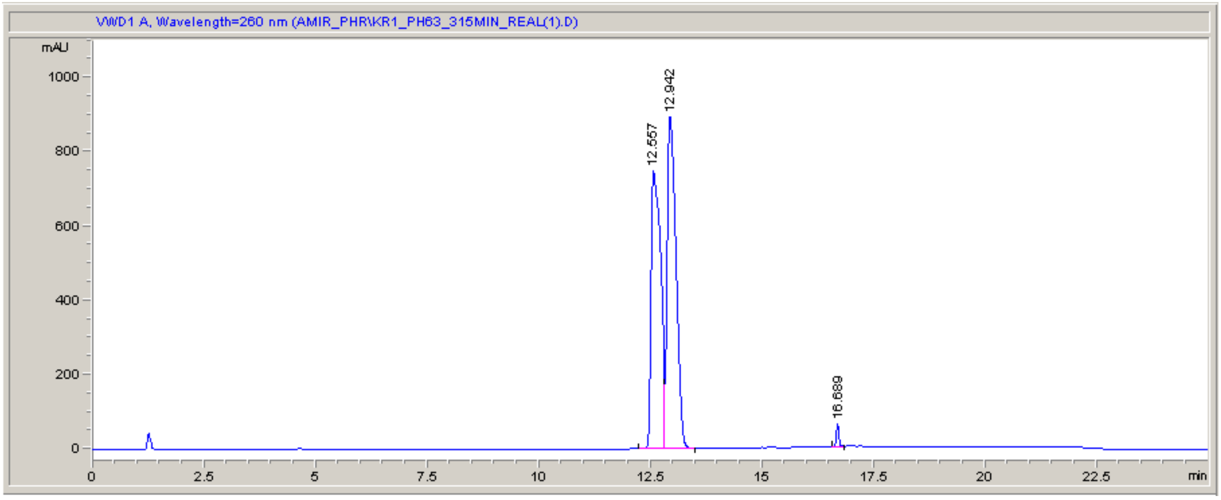

Figure S10. HPLC-UV chromatogram of the **IIIa**/GSH incubate (pH 6.3; 315 min sample). (**IIIa**: t_r16.69 min, **IIIa-GSH-1** conjugtate: t_r 12.56 min, **IIIa-GSH-2** conjugtate: t_r 12.94 min.)

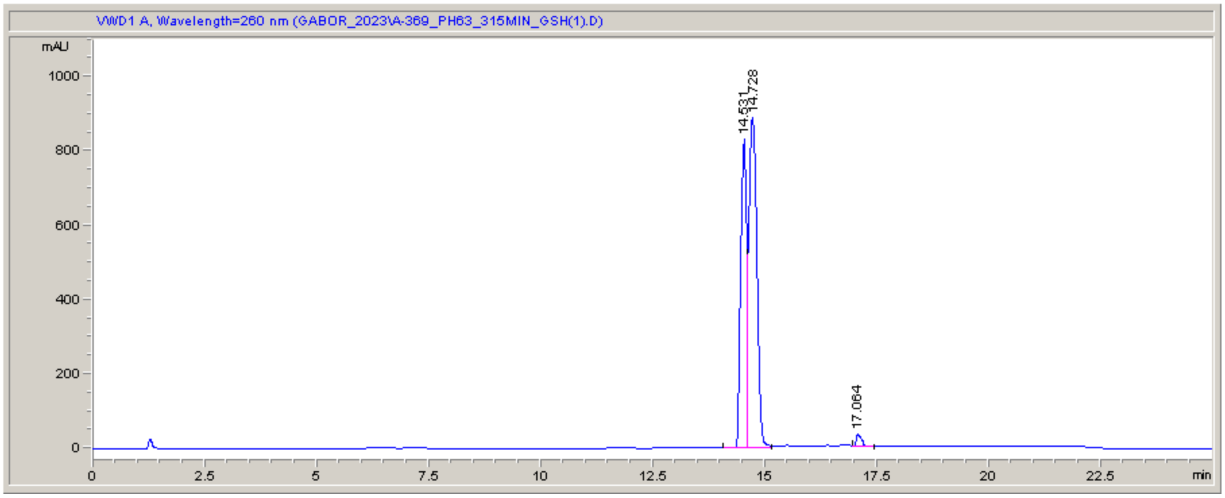

Figure S11. HPLC-UV chromatogram of the **IIIb**/GSH incubate (pH 6.3; 315 min sample). (**IIIb**: t_r17.06 min, **IIIb-GSH-1** conjugtate: t_r 14.53 min, **IIIb-GSH-2** conjugtate: t_r 14.73 min.)

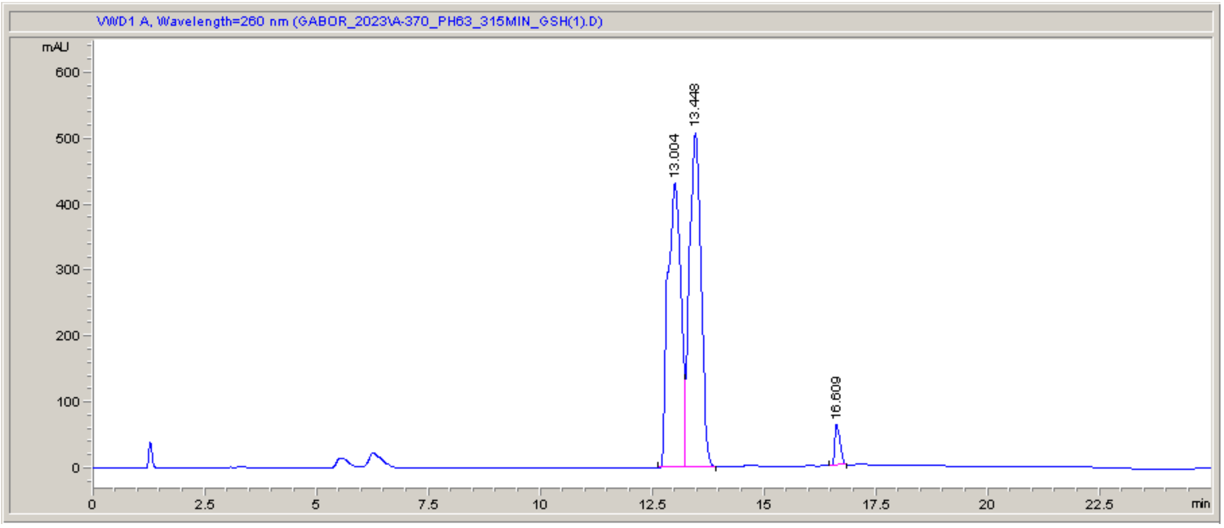

Figure S12. HPLC-UV chromatogram of the **IIIc**/GSH incubate (pH 6.3; 315 min sample). (**IIIc**: t_r16.61 min, **IIIc-GSH-1** conjugtate: t_r 13.00 min, **IIIc-GSH-2** conjugtate: t_r 13.45 min.)

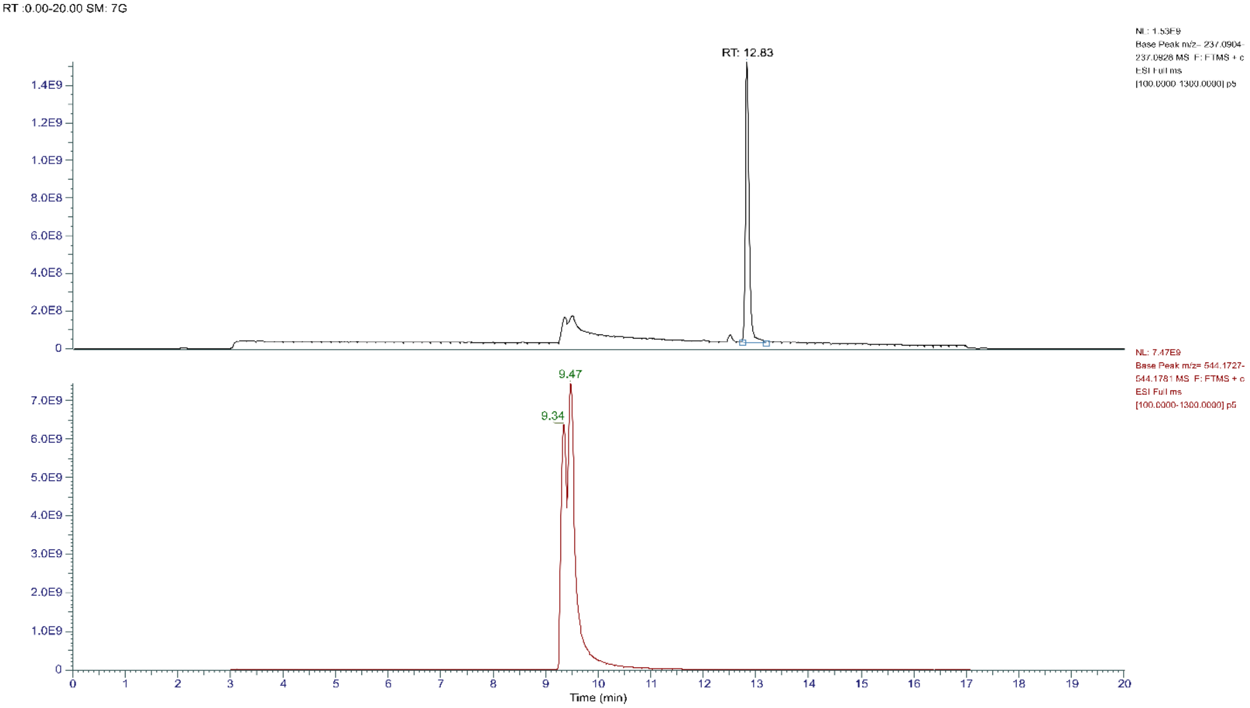

Figure S13. High resolution, positive mode HPLC-MS chromatograms of the **IIIa/GSH** incubate (pH 8.0 sample).
Upper panel: positive mode, extracted ion chromatogram of m/z 237.0916 [(**IIIa**)+H]$^+$ t_r 12.83 min.
Lower panel: positive mode, extracted ion chromatogram of m/z 544.1762 [(**IIIa-GSH**)+H]$^+$ t_r 9.34 and 9.47 min.

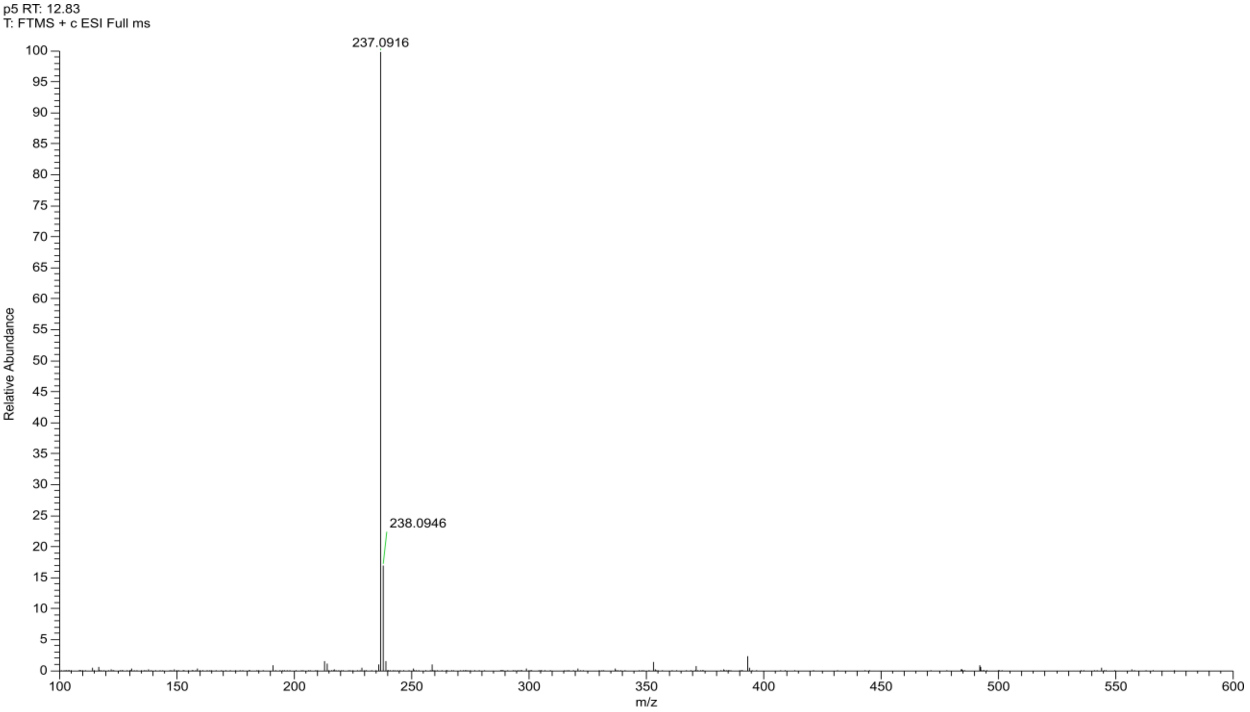

Figure S14. High resolution, positive mode HESI MS spectrum of the **IIIa** (t_r 12.83 min) in the sample of pH 8.0 incubate, (237.0916 [(**IIIa**)+H]$^+$).

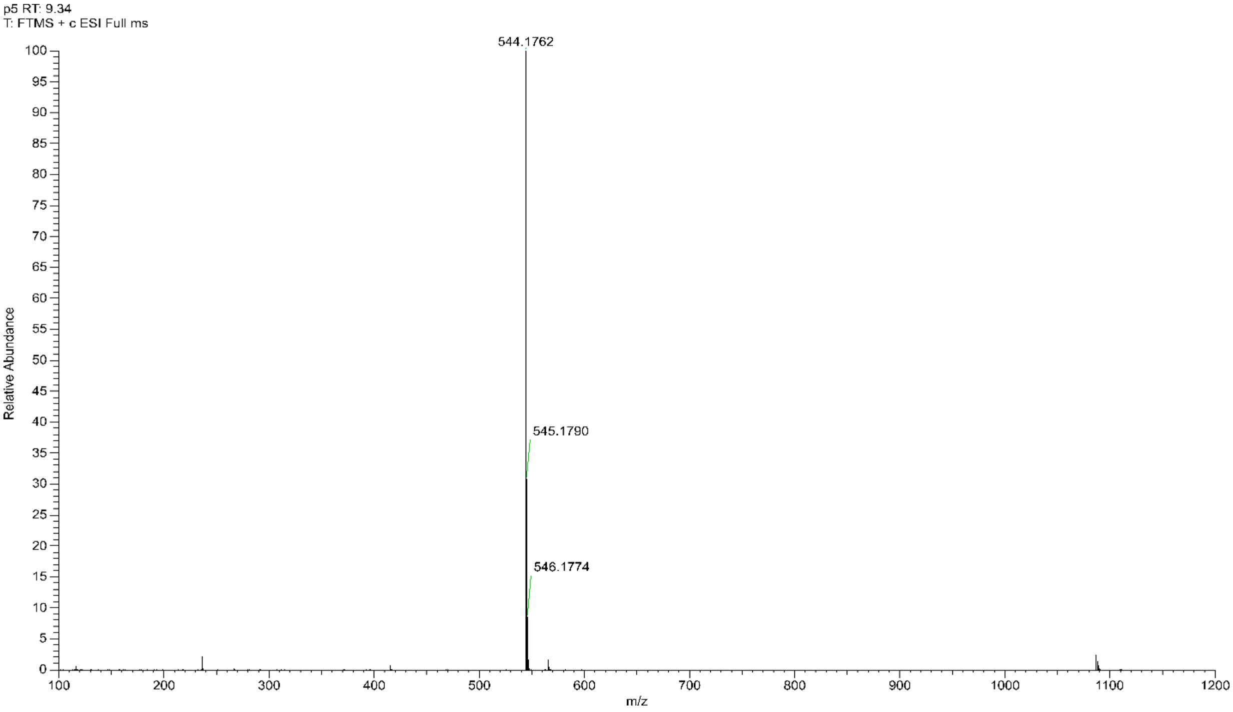

Figure S15. High resolution, positive mode HESI MS spectrum of the **IIIa-GSH-1** conjugate (t_r 9.34 min) formed in the sample of the pH 8.0 incubate, (m/z 544.1762 [(**IIIa-GSH**)+H]$^+$).

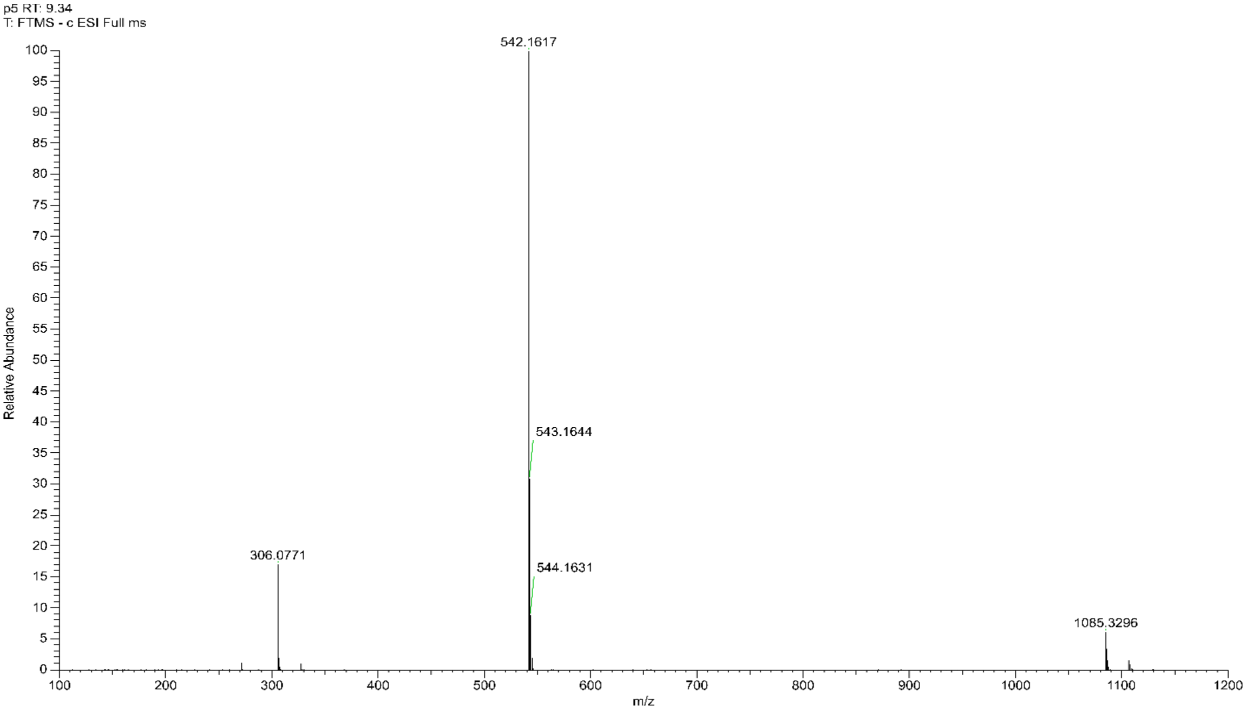

Figure S16. High resolution, negative mode HESI MS spectrum of the **IIIa-GSH-1** conjugate (t_r 9.34 min) formed in the sample of the pH 8.0 incubate, (m/z 542.1617 [(**IIIa-GSH**)-H]$^-$), m/z 306.0771 [**GSH**-H]$^-$) and m/z 1085.3296 [(**IIIa-GSH**)$_2$-H]$^-$).

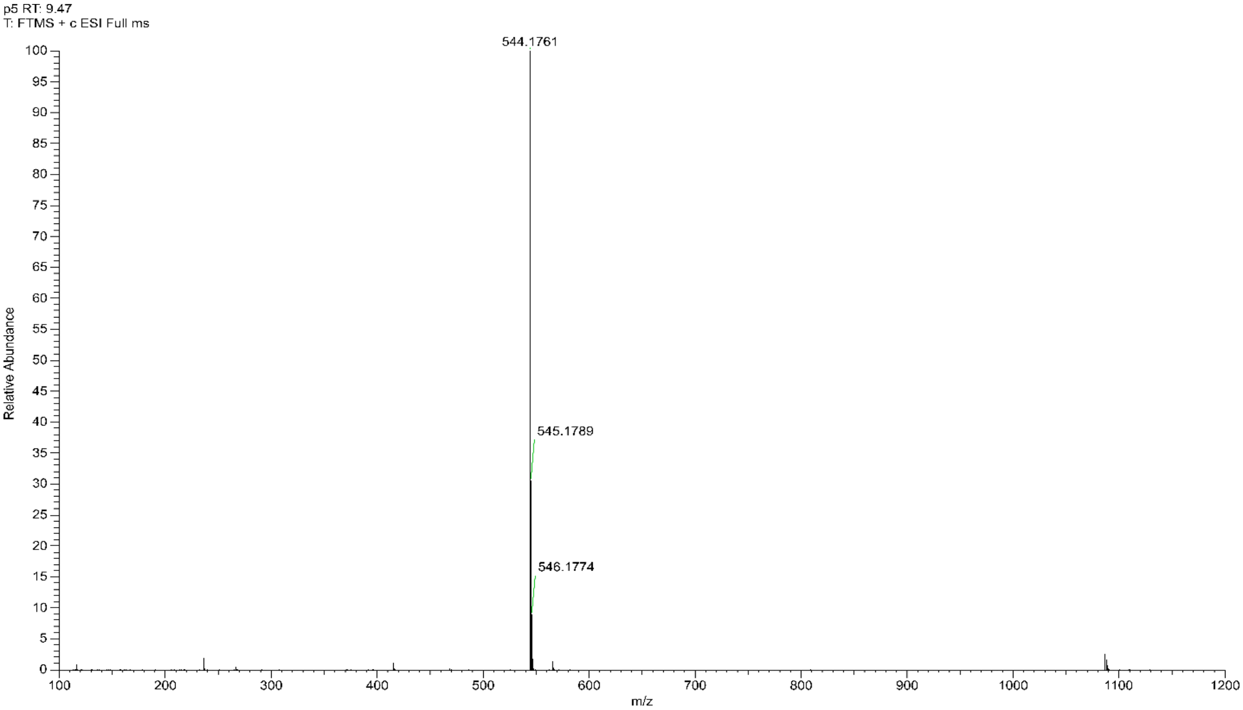

Figure S17. High resolution, positive mode HESI MS spectrum of the **IIIa-GSH-2** conjugate (t_r 9.47 min) formed in the sample of the pH 8.0 incubate, (m/z 544.1761 [(**IIIa-GSH**)+H]$^+$).

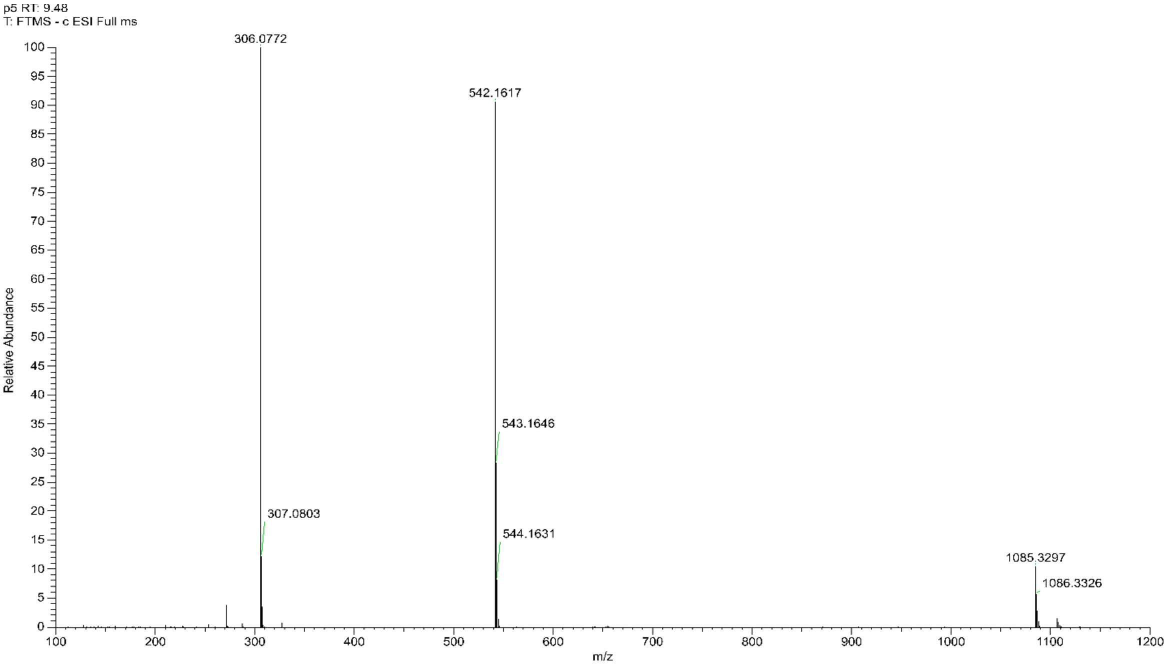

Figure S18. High resolution, negative mode HESI MS spectrum of the **IIIa-GSH-2** conjugate (t_r 9.48 min) formed in the sample of the pH 8.0 incubate, (m/z 542.1617 [(**IIIa-GSH**)-H]$^-$, m/z 306.0772 [**GSH**-H]$^-$ and m/z 1085.3297 [(**IIIa-GSH**)$_2$-H]$^-$).

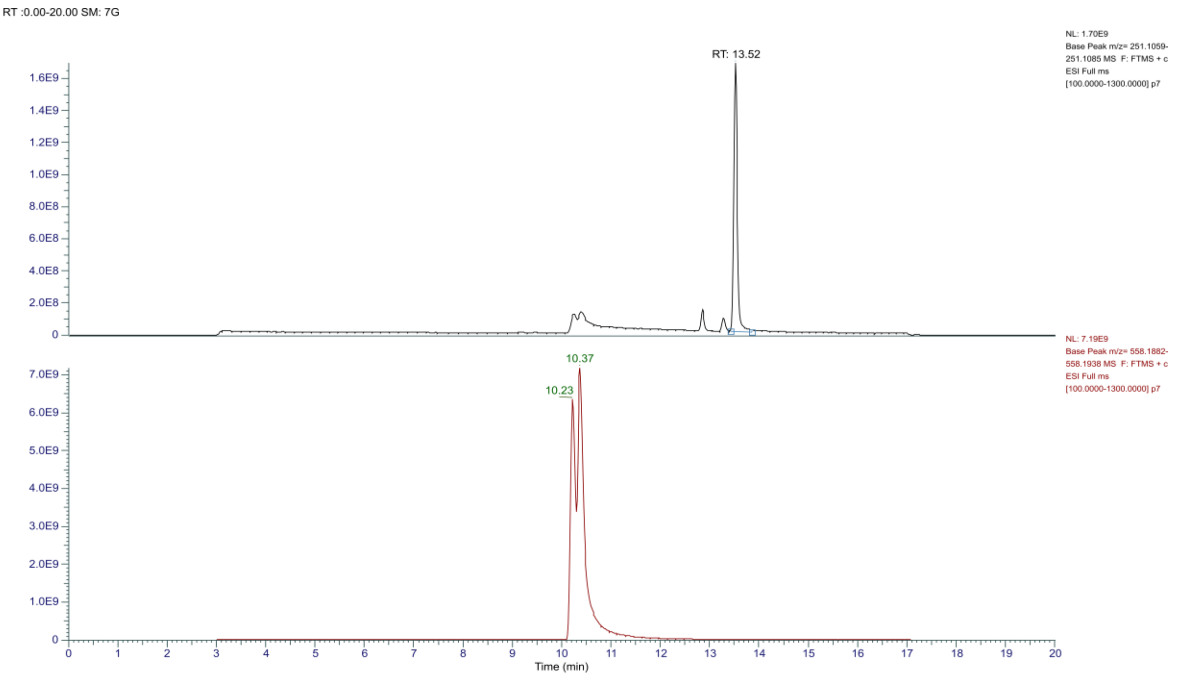

Figure S19. High resolution, positive mode HPLC-MS chromatograms of the **IIIb/GSH** incubate (pH 8.0 sample).
Upper panel: positive mode, extracted ion chromatogram of m/z 251.1072 [(**IIIb**)+H]$^+$ t_r 13.52 min.
Lower panel: positive mode, extracted ion chromatogram of m/z 558.1918 [(**IIIb-GSH**)+H]$^+$ t_r 10.23 and 10.37 min.

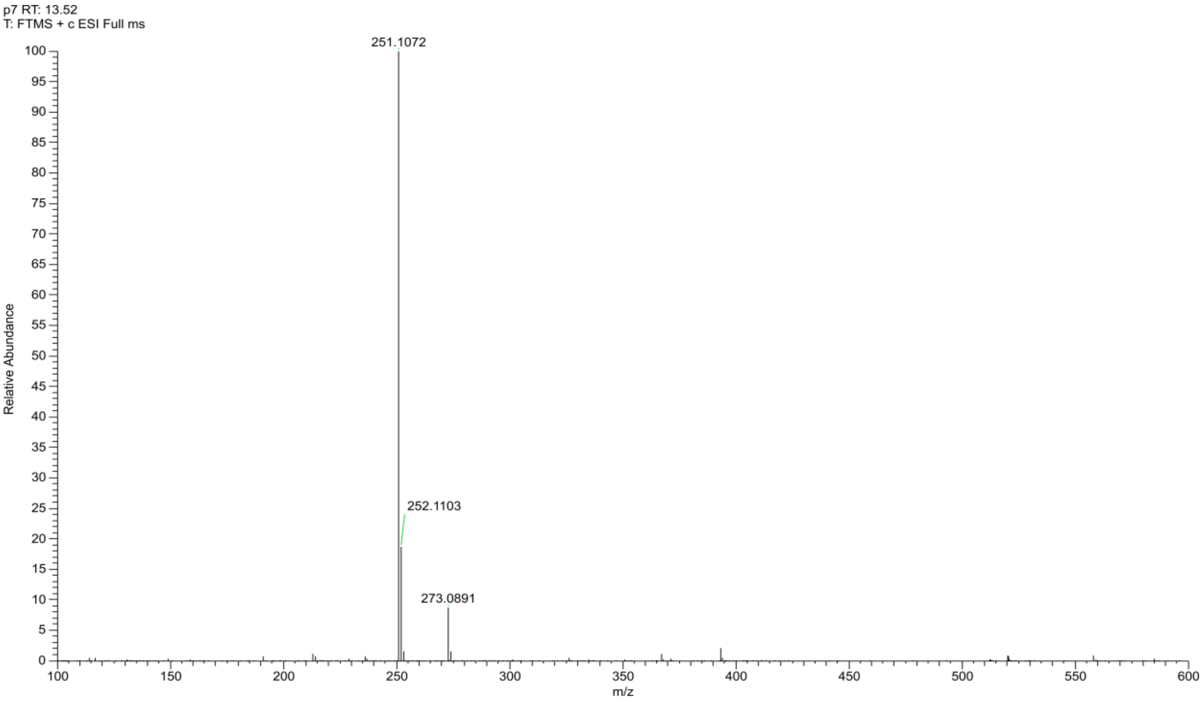

Figure S20. High resolution, positive mode HESI MS spectrum of the **IIIb** (t_r 13.52 min) in the sample of the pH 8.0 incubate, (m/z 251.1072 [(**IIIb**)+H]$^+$ and m/z 273.0891 [(**IIIb**)+Na]$^+$).

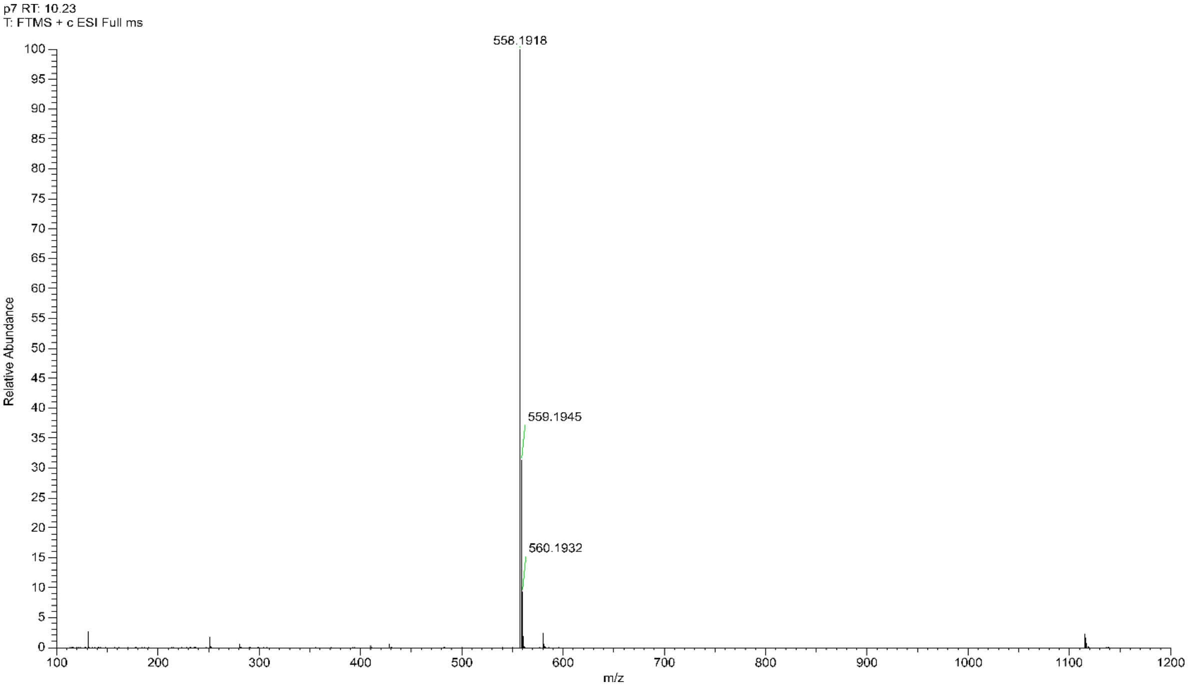

Figure S21. High resolution, positive mode HESI MS spectrum of the **IIIb-GSH-1** conjugate (t_r 10.23 min) formed in the sample of the pH 8.0 incubate, (m/z 558.1918 [(**IIIb-GSH**)+H]$^+$).

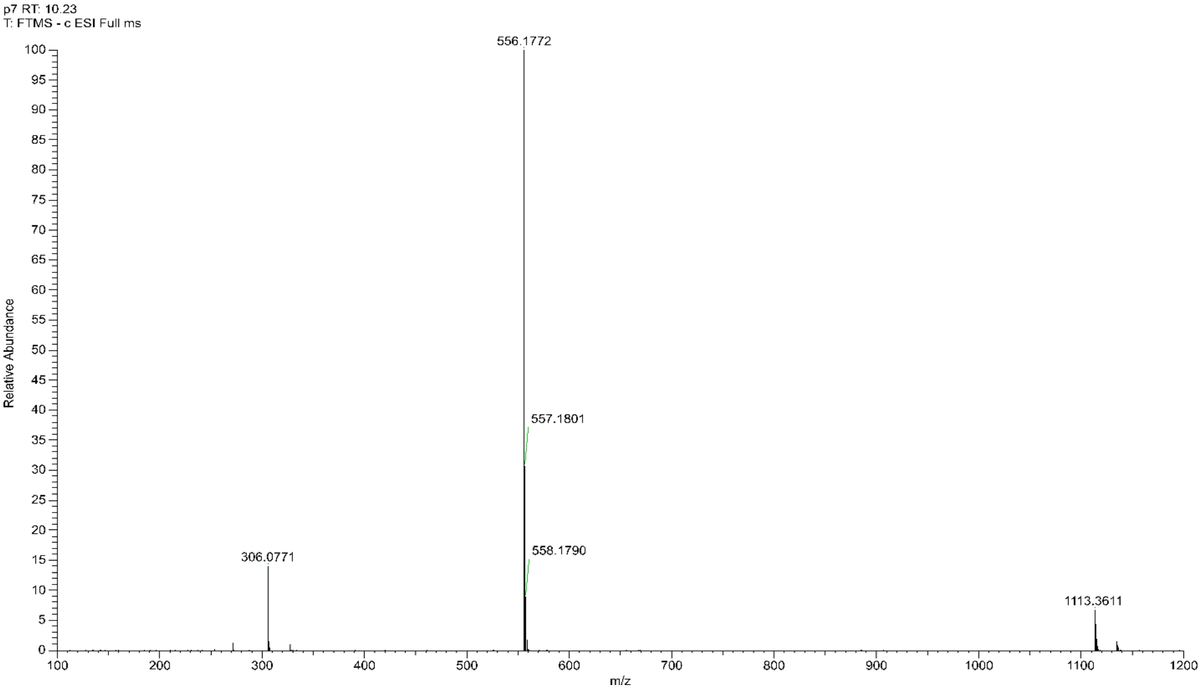

Figure S22. High resolution, negative mode HESI MS spectrum of the **IIIb-GSH-1** conjugate (t_r 10.23 min) formed in the sample of the pH 8.0 incubate, (m/z 556.1772 [(**IIIb-GSH**)-H]$^-$, m/z 306.0771 [**GSH**-H]$^-$ and m/z 1113.3611 [(**IIIb-GSH**)$_2$-H]$^-$).

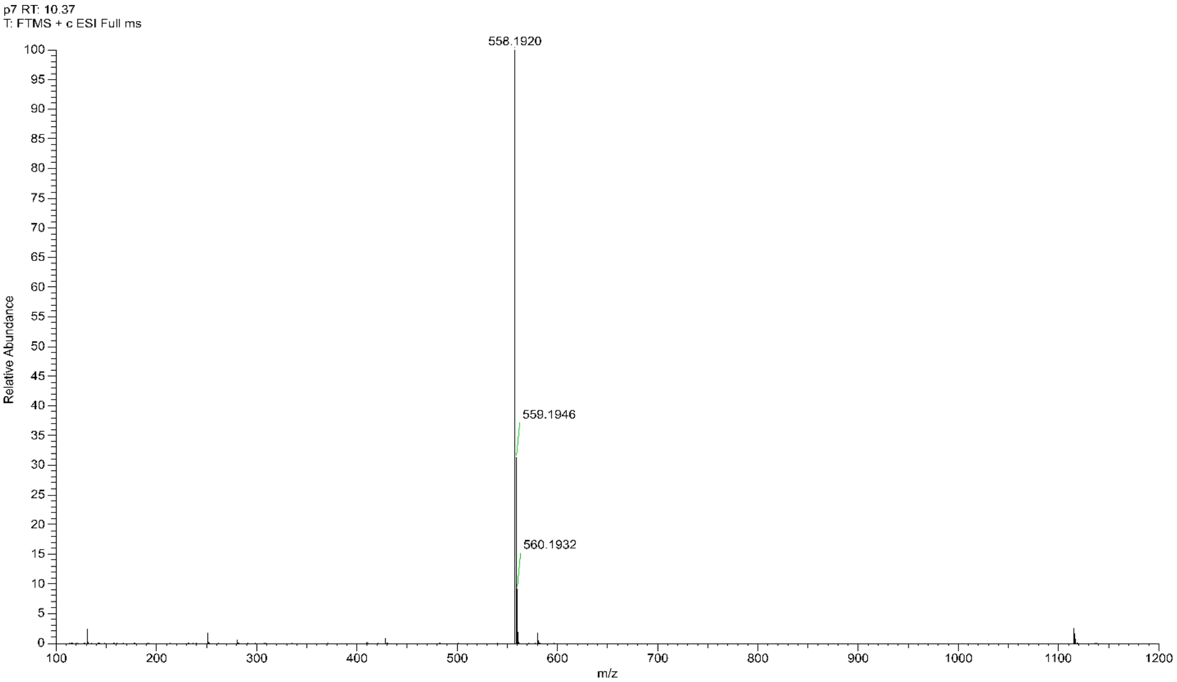

Figure S23. High resolution, positive mode HESI MS spectrum of the **IIIb-GSH-2** conjugate (t_r 10.37 min) formed in the sample of the pH 8.0 incubate, (m/z 558.1920 [(**IIIb-GSH**)+H]$^+$).

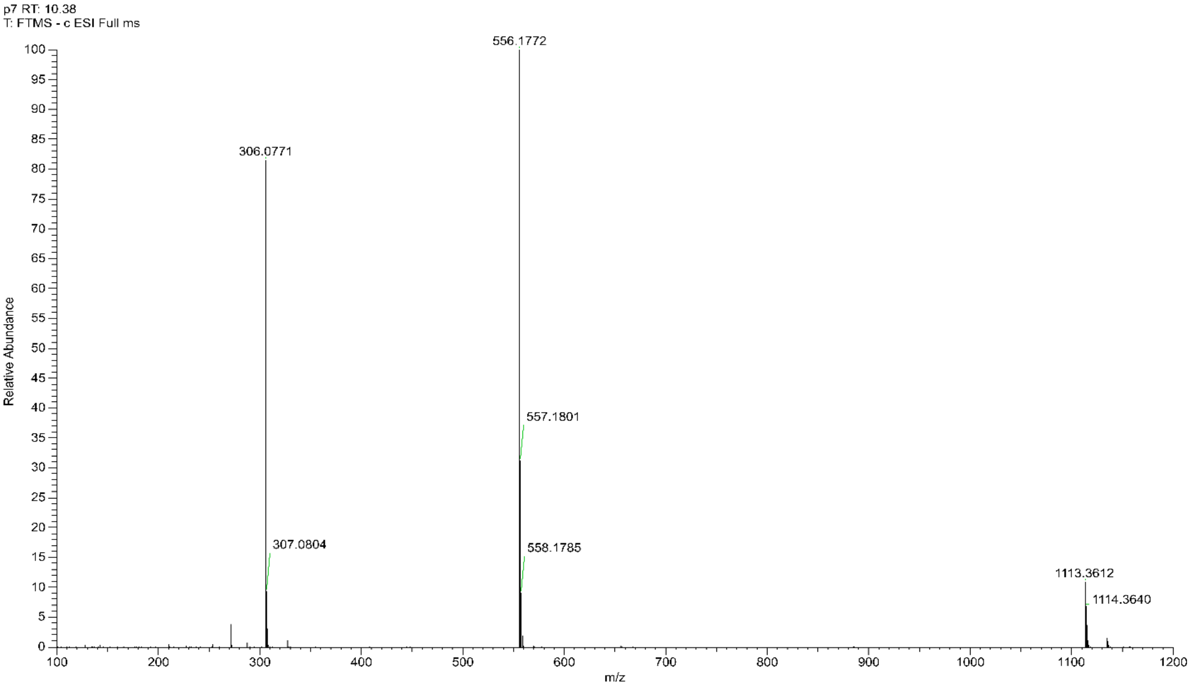

Figure S24. High resolution, negative mode HESI MS spectrum of the **IIIb-GSH-2** conjugate (t_r 10.38 min) formed in the sample of the pH 8.0 incubate, (m/z 556.1772 [(**IIIb-GSH**)-H]$^-$, m/z 306.0771 [**GSH**-H]$^-$ and m/z 1113.3612 [(**IIIb-GSH**)$_2$-H]$^-$).

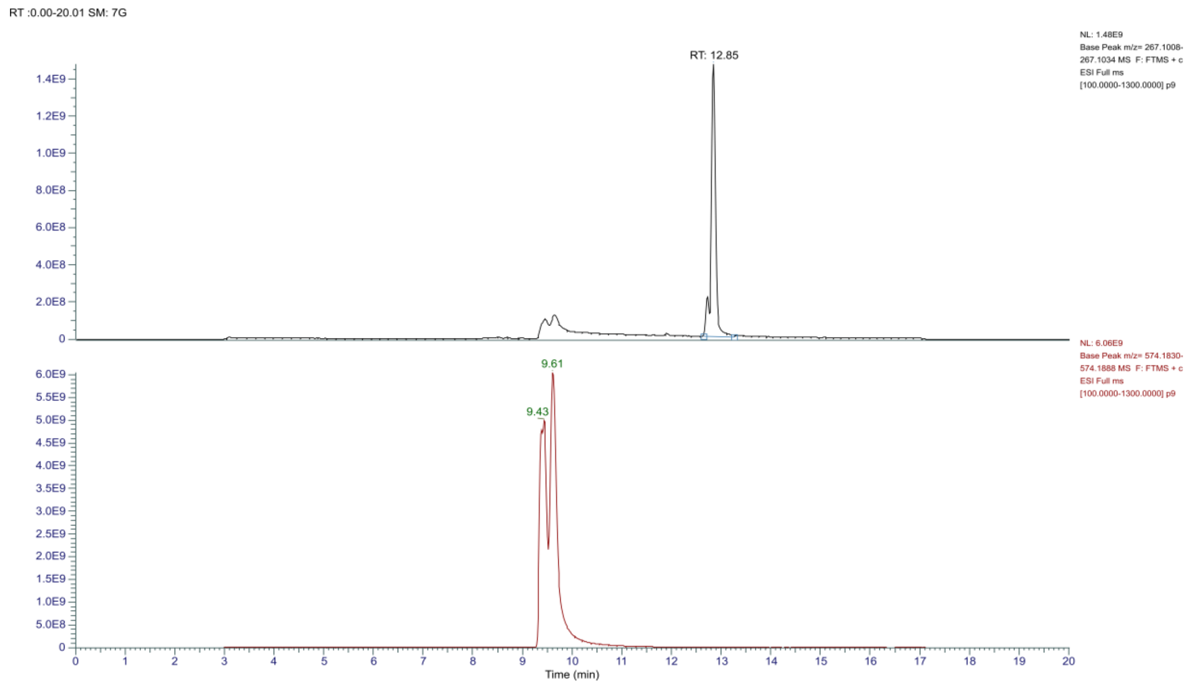

Figure S25. High resolution, positive mode HPLC-MS chromatograms of the **IIIc-GSH** incubate (pH 8.0 sample).
Upper panel: positive mode, extracted ion chromatogram of m/z 267.1021[(**IIIc**)+H]$^+$ t_r 12.85 min.
Lower panel: positive mode, extracted ion chromatogram of m/z 574.1871 [(**IIIc-GSH**)+H]$^+$ t_r 9.43 and 9.61 min.

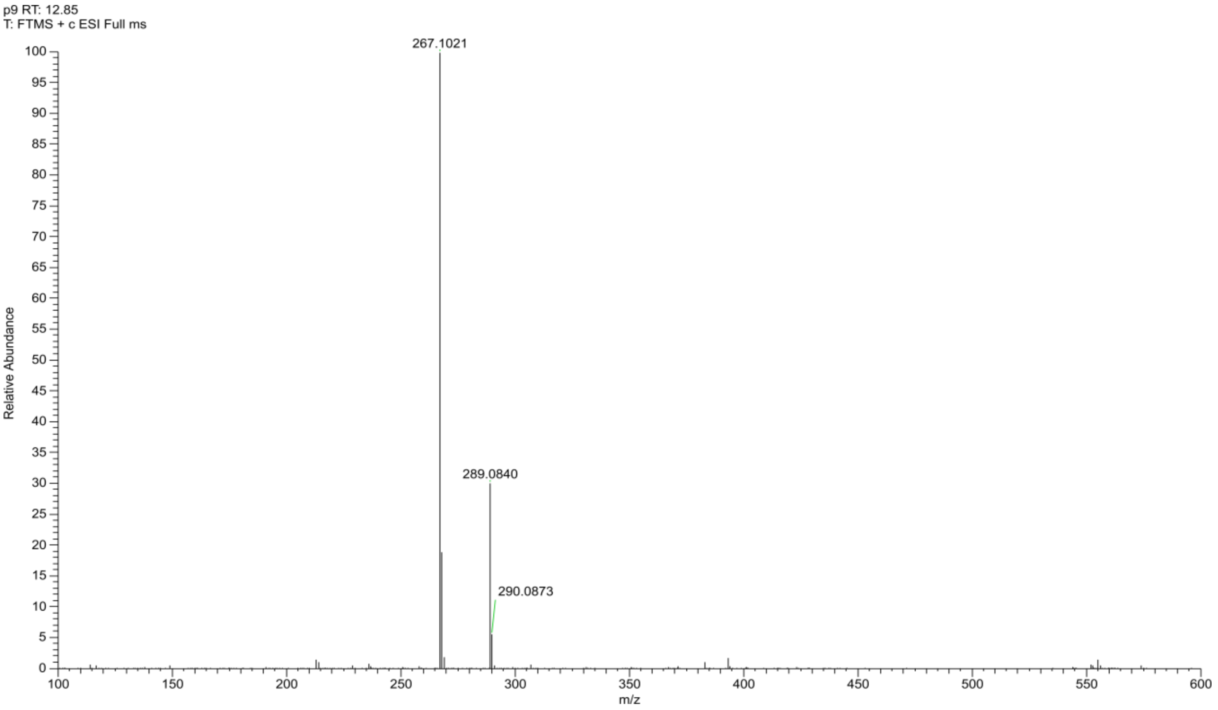

Figure S26. High resolution, positive mode HESI MS spectrum of the **IIIc** (t_r 12.85 min) in the sample of the pH 8.0 incubate, (m/z 267.1021 [(**IIIc**)+H]$^+$ and m/z 289.0840 [(**IIIc**)+Na]$^+$).

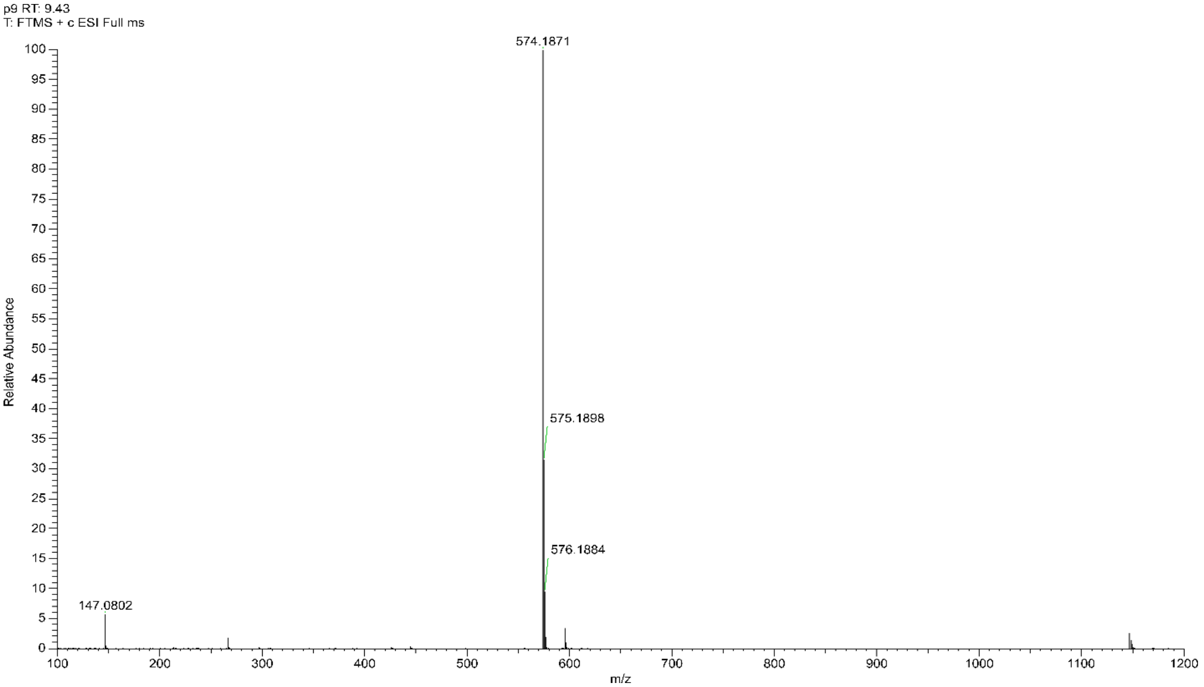

Figure S27. High resolution, positive mode HESI MS spectrum of the **IIIc-GSH-1** conjugate (t_r 9.43 min) formed in the sample of the pH 8.0 incubate, (m/z 574.1871 [(**IIIc-GSH**)+H]$^+$).

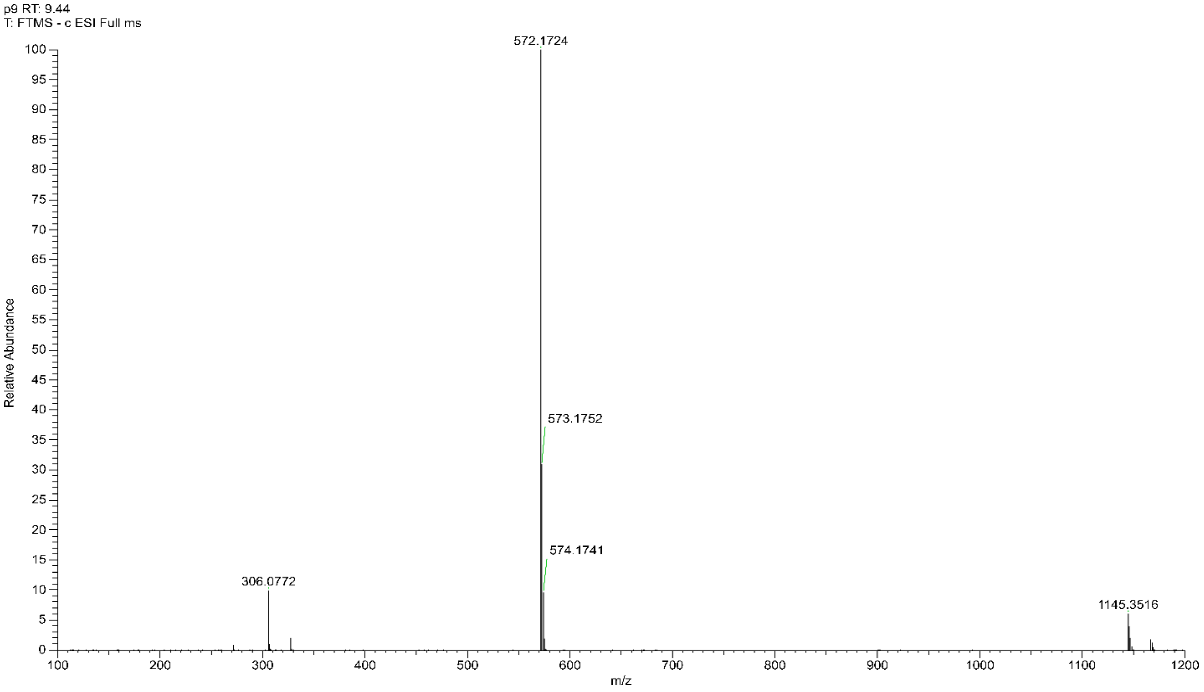

Figure S28. High resolution, negative mode HESI MS spectrum of the **IIIc-GSH 1** conjugate (t_r 9.44 min) formed in the sample of the pH 8.0 incubate, (m/z 572.1724 [(**IIIc-GSH**)-H]$^-$, m/z 306.0772 [**GSH**-H]$^-$ and m/z 1145.3516 [(**IIIc-GSH**)$_2$-H]$^-$).

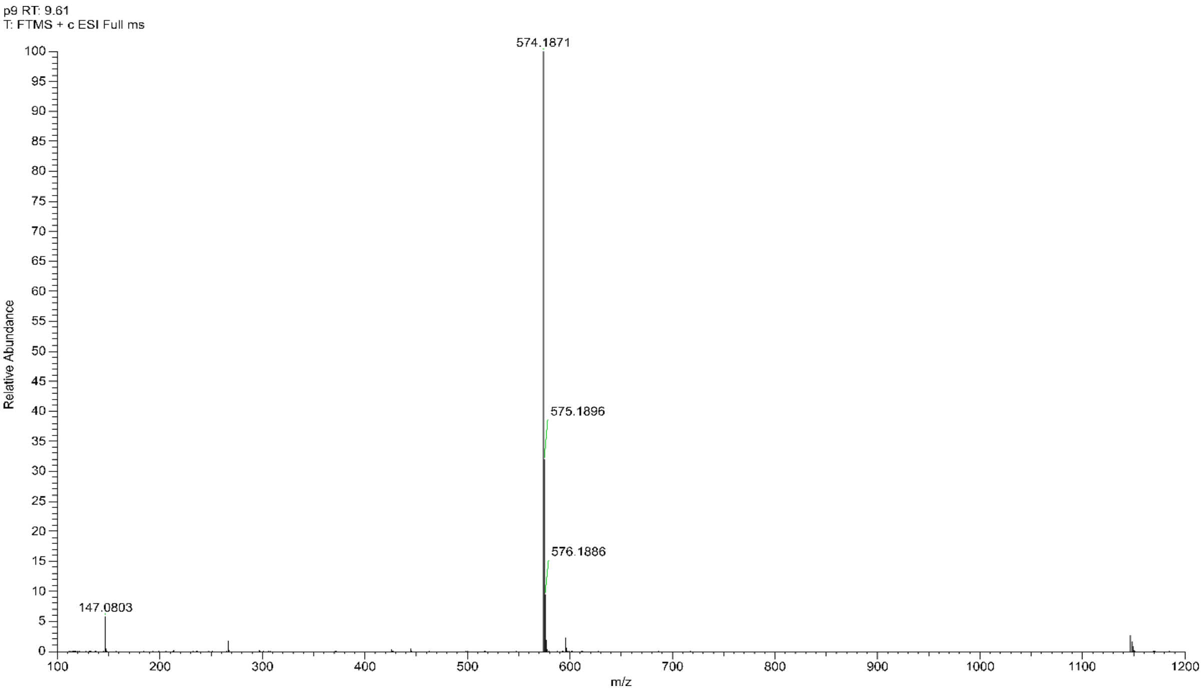

Figure S29. High resolution, positive mode HESI MS spectrum of the **IIIc-GSH-2** conjugate (t_r 9.61 min) formed in the sample of the pH 8.0 incubate, (m/z 574.1871 [(**IIIc-GSH**)+H]$^+$).

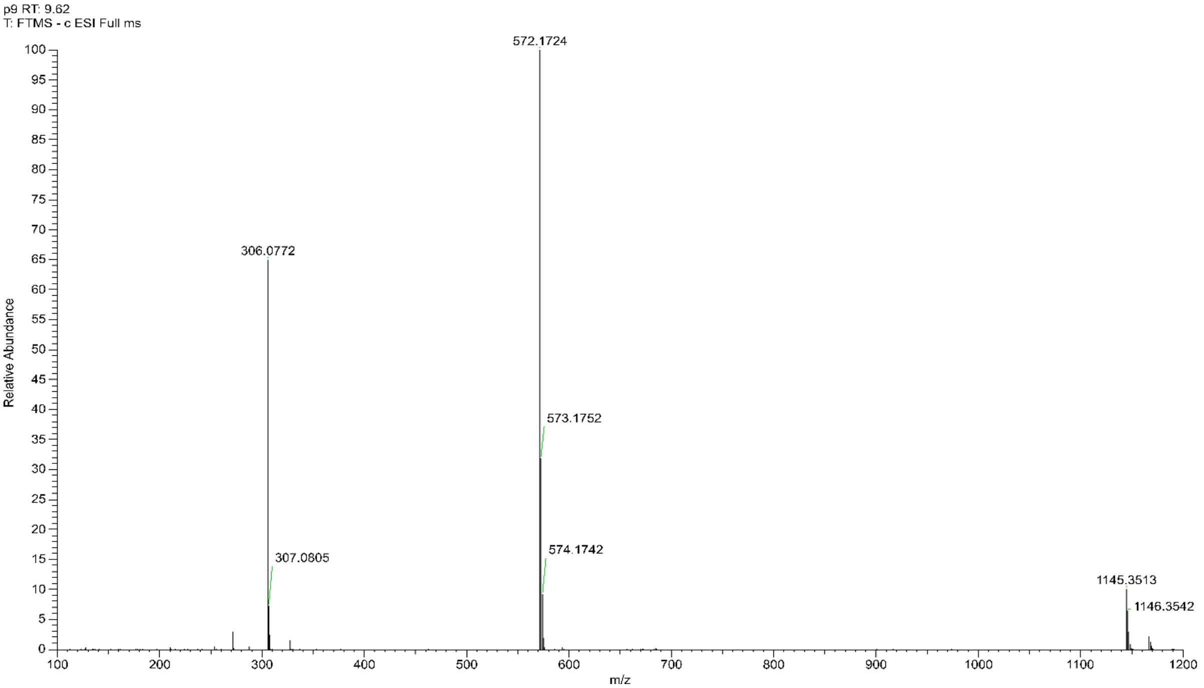

Figure S30. High resolution, negative mode HESI MS spectrum of the **IIIc-GSH-2** conjugate (t_r 9.62 min) formed in the sample of the pH 8.0 incubate, (m/z 572.1724 [(**IIIc-GSH**)-H]$^-$, m/z 306.0772 [**GSH**-H]$^-$ and m/z 1145.3513 [(**IIIc-GSH**)$_2$-H]$^-$).

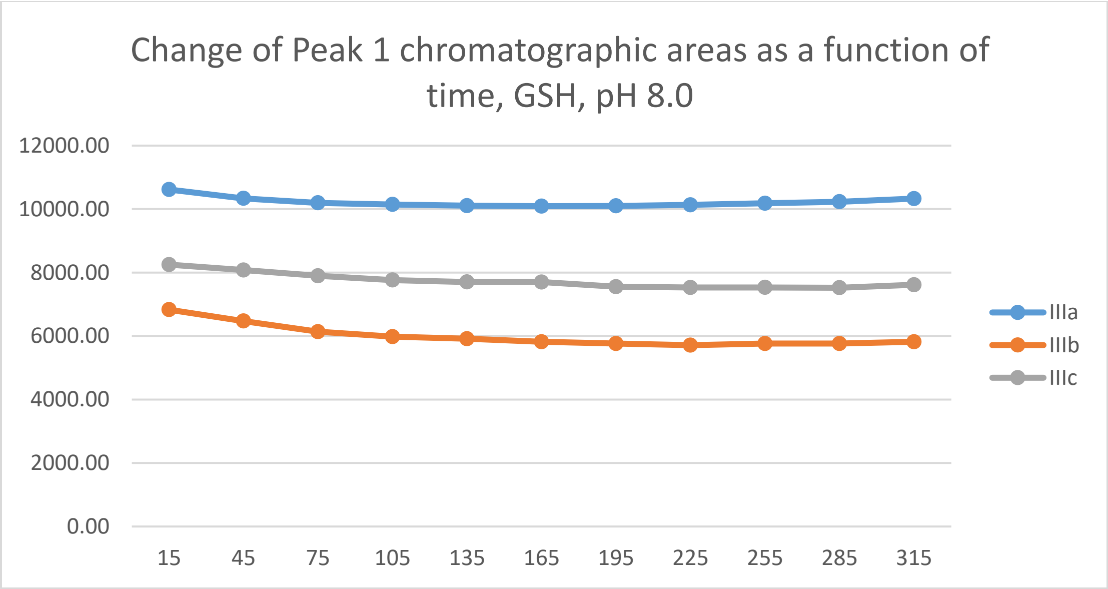

Figure S31. Change in the HPLC-UV chromatographic peak area of adduct-1 of **IIIa, IIIb**, and **IIIc** as a function of time (min) in the chalcone/GSH incubations at pH 8.0. Each data point represents the average of two independent measurements.

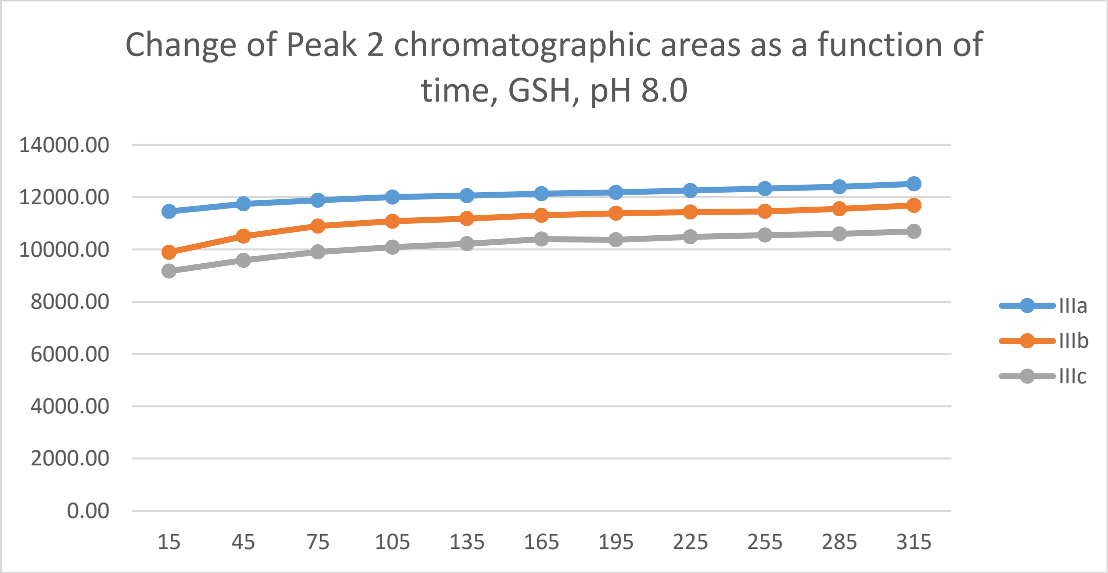

Figure S32. Change in the HPLC-UV chromatographic peak area of adduct-2 of **IIIa, IIIb,** and **IIIc** as a function of time (min) in the chalcone/GSH incubations at pH 8.0. Each data point represents the average of two independent measurements.

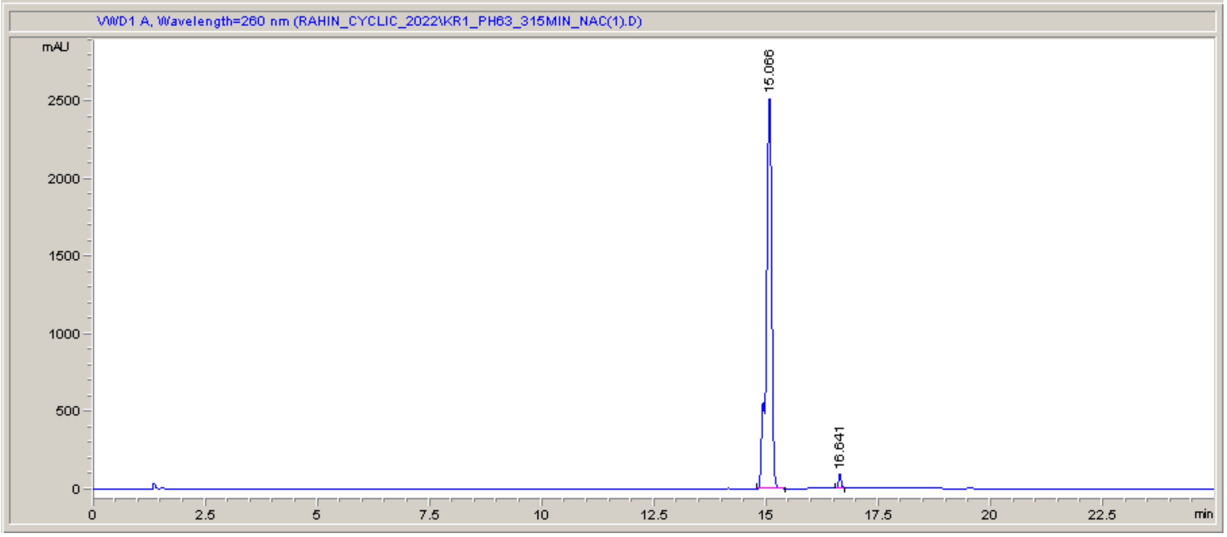

Figure S33. HPLC-UV chromatogram of the **IIIa**/NAC incubate (pH 6.3; 315 min sample). (**IIIa**: t_r16.64 min, **IIIa-NAC-1** conjugtate: t_r 15.07 min.)

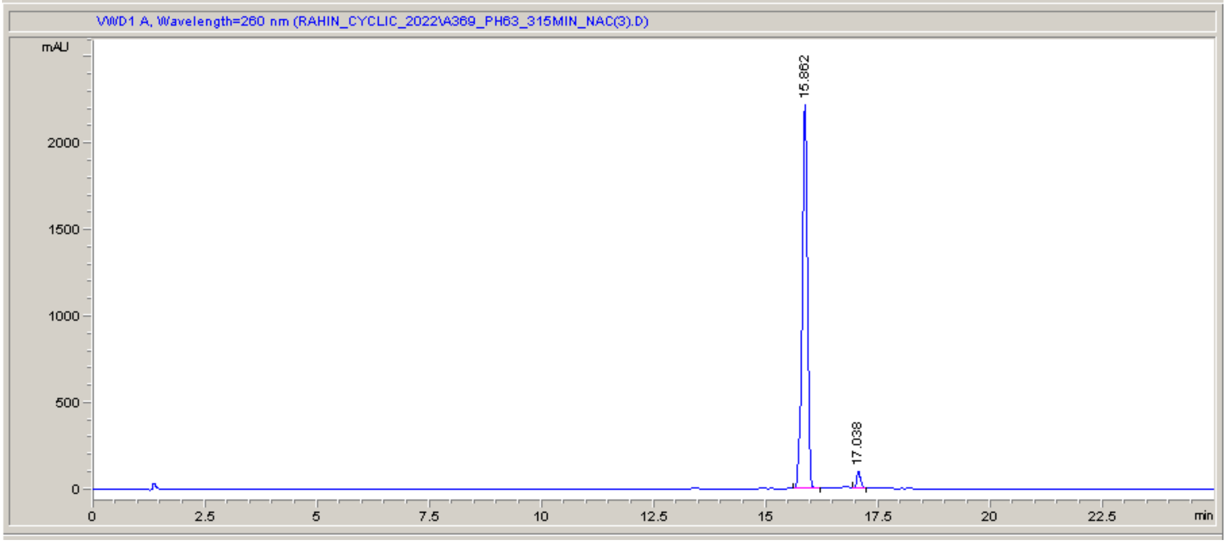

Figure S34. HPLC-UV chromatogram of the **IIIb**/NAC incubate (pH 6.3; 315 min sample). (**IIIb**: t_r17.04 min, **IIIb-NAC-1** conjugtate: t_r 15.86 min.)

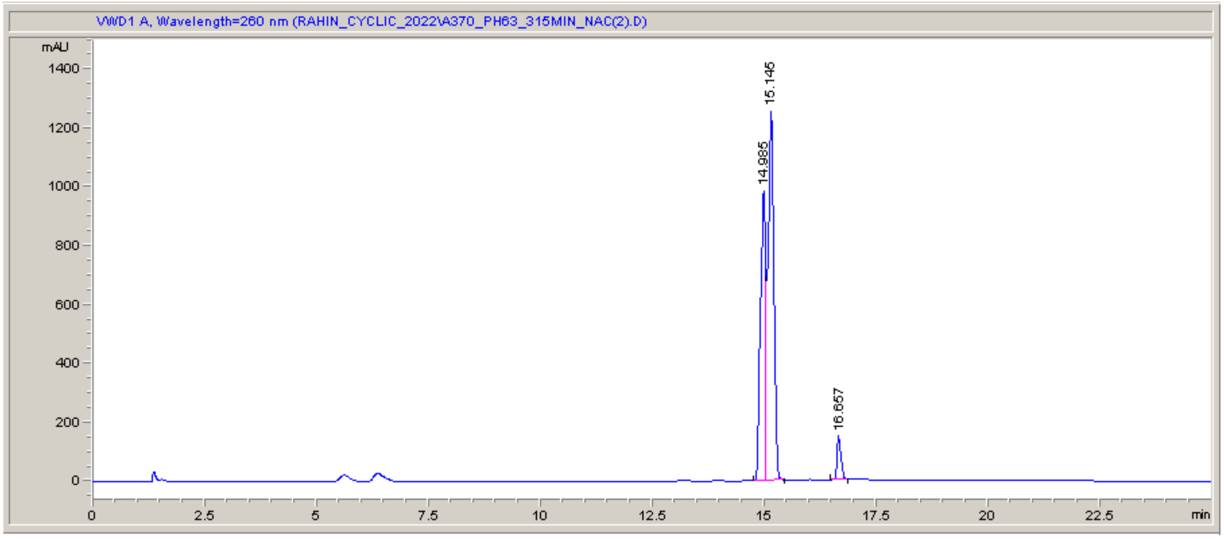

Figure S35. HPLC-UV chromatogram of the **IIIc**/NAC incubate (pH 6.3; 315 min sample). (**IIIc**: t_r16.66 min, **IIIc-NAC-1** conjugtate: t_r 14.99 min, **IIIc-NAC-2** conjugtate: t_r 15.15 min.)

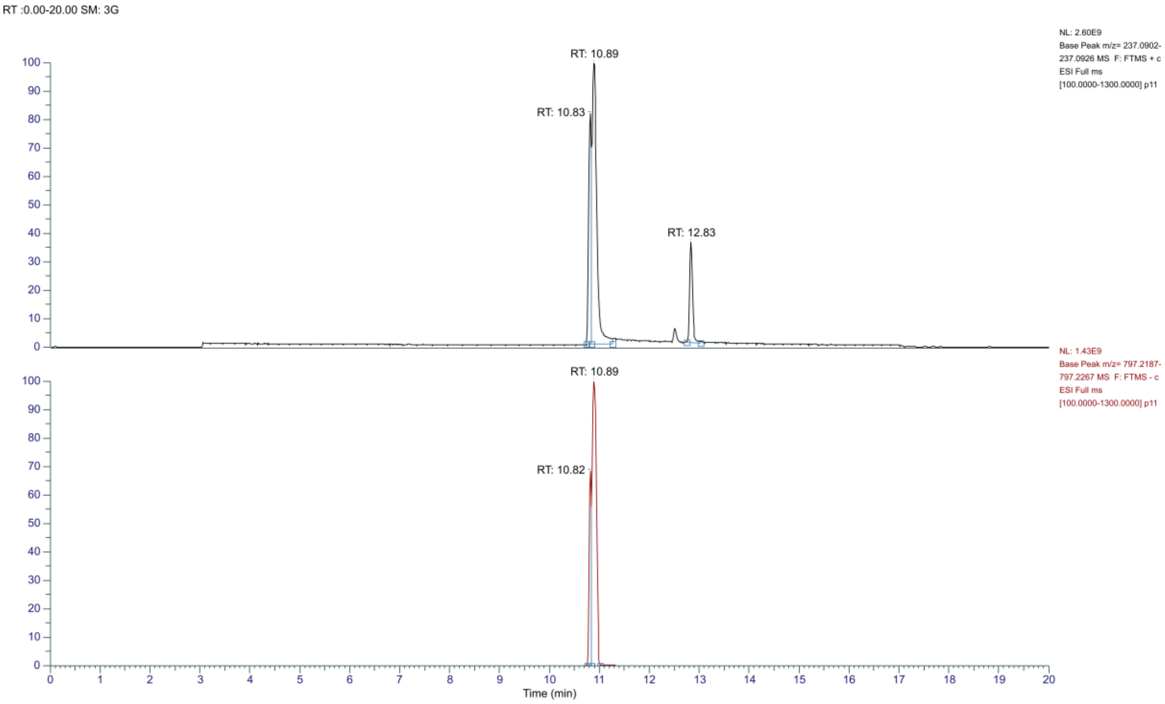

Figure S36. High resolution, HPLC-MS chromatograms of **IIIa/NAC** incubate (pH 8.0 sample).
Upper panel: positive mode m/z 237.0914 [**IIIa**+H]$^+$ t_r 12.83 min: **IIIa**
Lower panel: negative mode m/z 797.2227 [(**IIIa-NAC**)$_2$-H]$^-$ t_r 10.82 min: **IIIa-NAC-1**, t_r 10.89 min: **IIIa-NAC-2**.

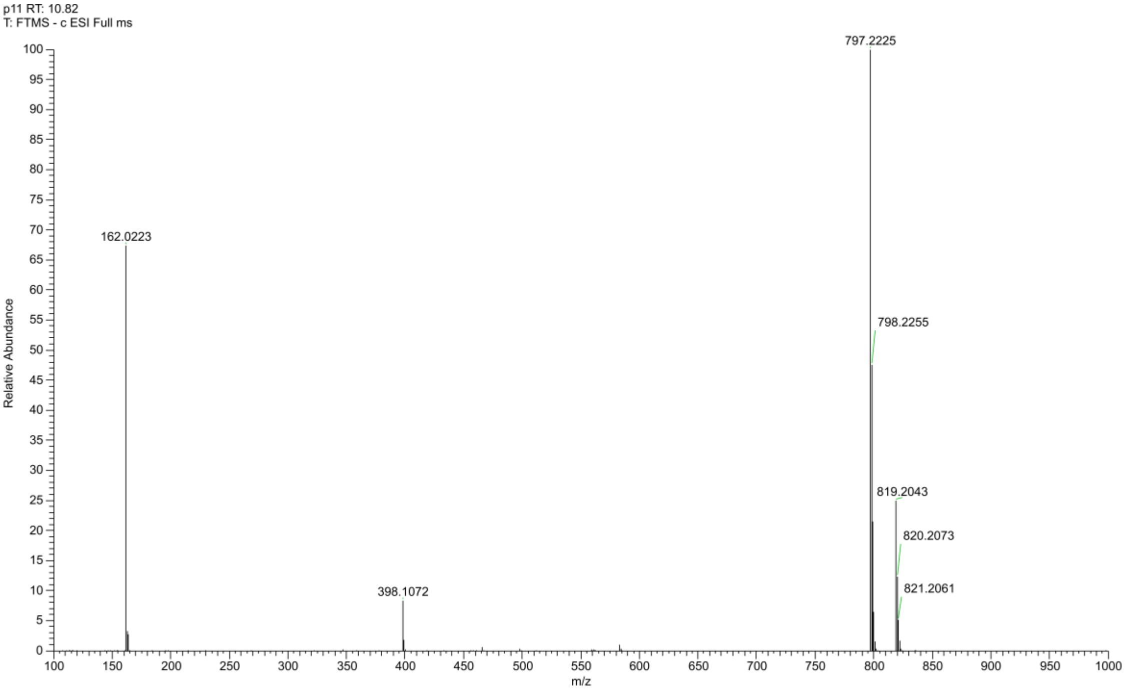

Figure S37. High resolution, negative mode HESI MS spectrum of the **IIIa-NAC-1** conjugate (t_r 10.82 min) formed in the sample of the pH 8.0 incubate. (m/z 398.1072 [(**IIIa-NAC**)-H]$^-$, m/z 797.2227 [(**IIIa-NAC**)$_2$-H]$^-$, m/z 819.2043 [(**IIIa-NAC**)$_2$+Na-2H]$^-$).

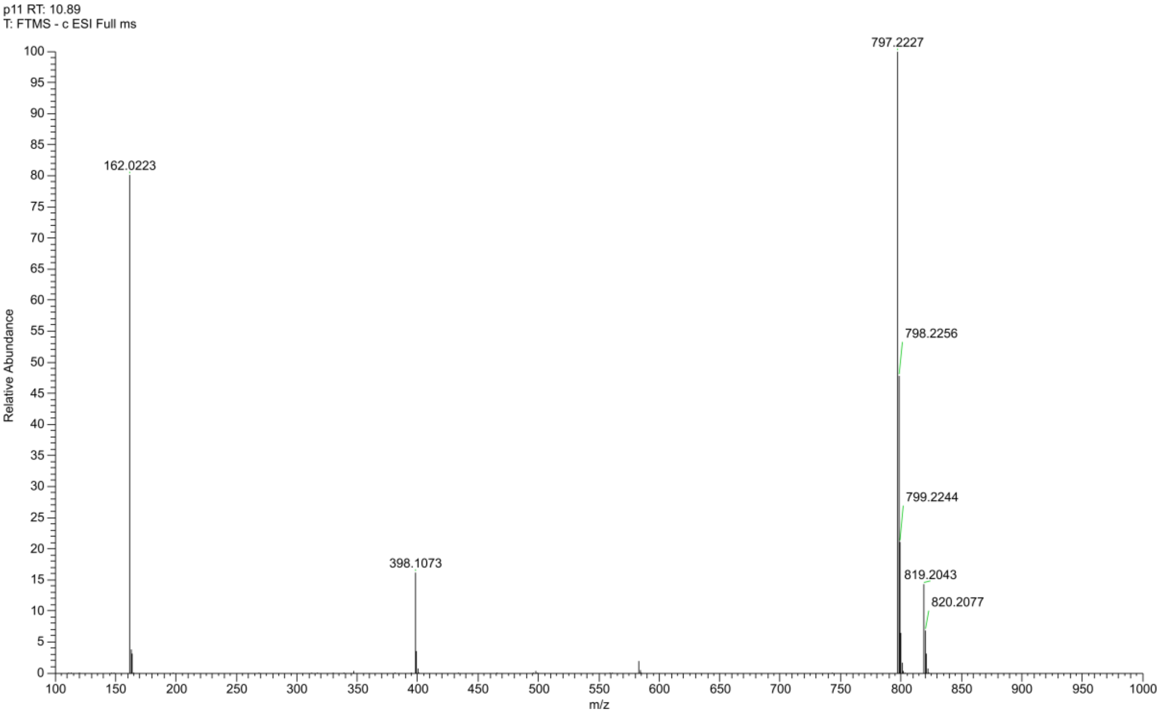

Figure S38. High resolution, negative mode HESI MS spectrum of the **IIIa-NAC-2** conjugate (t_r 10.89 min) formed in the sample of the pH 8.0 incubate. (m/z 398.1073 [(**IIIa-NAC**)-H]$^-$, m/z 797.2227 [(**IIIa-NAC**)$_2$-H]$^-$, m/z 819.2043 [(**IIIa-NAC**)$_2$+Na-2H]$^-$).

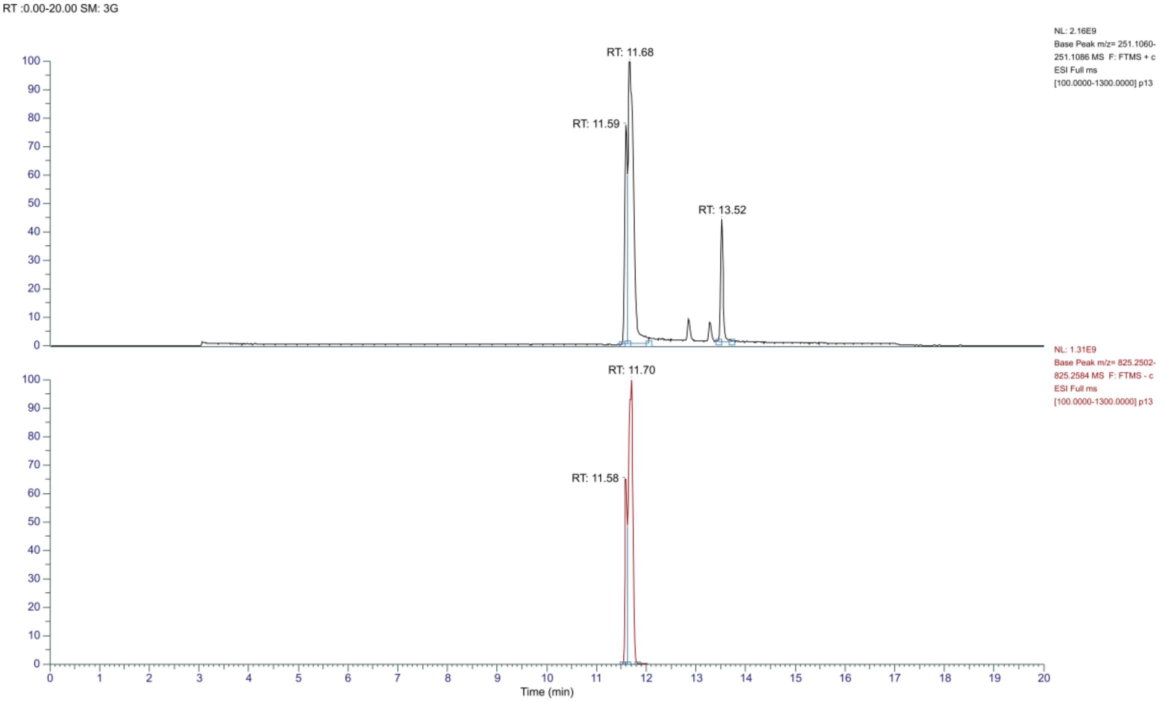

Figure S39. High resolution, HPLC-MS chromatograms of **IIIb**/**NAC** incubate (pH 8.0 sample).
Upper panel: positive mode m/z 251.1073 [**IIIb**+H]$^+$ t_r 13.52 min: **IIIb**.
Lower panel: negative mode m/z 825.2541 [(**IIIb-NAC**)$_2$-H]$^-$ t_r 11.58 min: **IIIb-NAC-1**, t_r 11.70 min: **IIIb-NAC-2**.

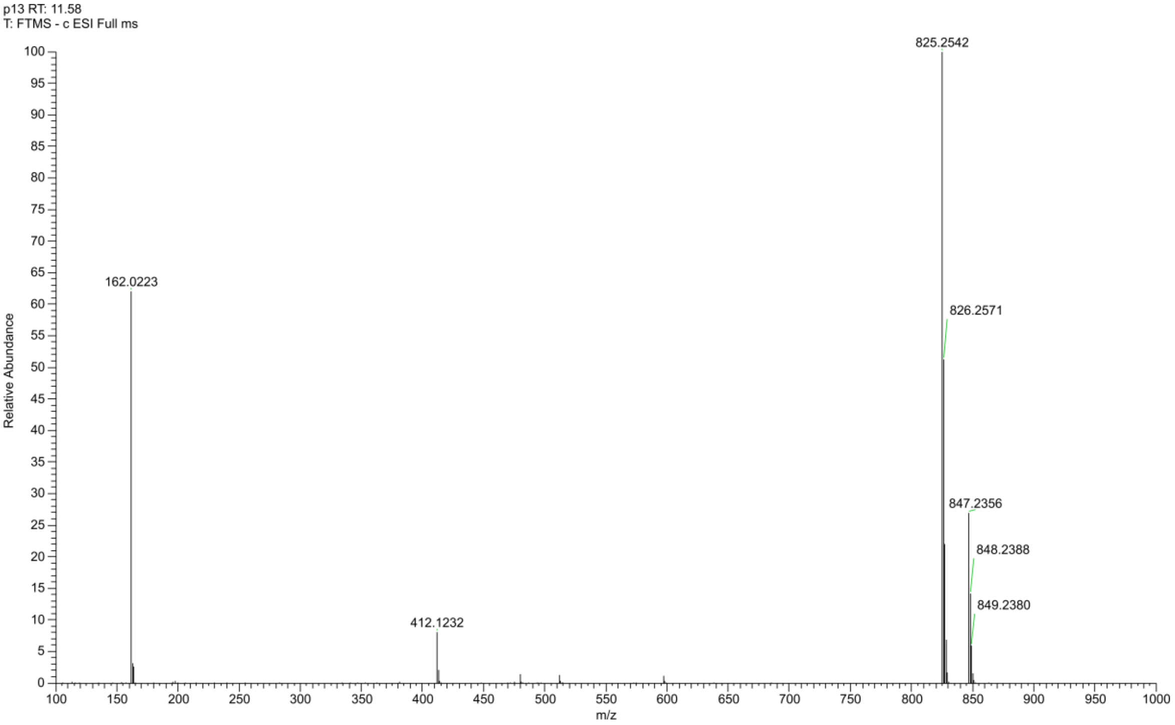

Figure S40. High resolution, negative mode HESI MS spectrum of the **IIIb-NAC-1** conjugate (t_r 11.58 min) formed in the sample of the pH 8.0 incubate. (m/z 412.1232 [(**IIIb-NAC**)-H]$^-$, m/z 825.2541 [(**IIIb-NAC**)$_2$-H]$^-$, m/z 847.2354 [(**IIIb-NAC**)$_2$+Na-2H]$^-$).

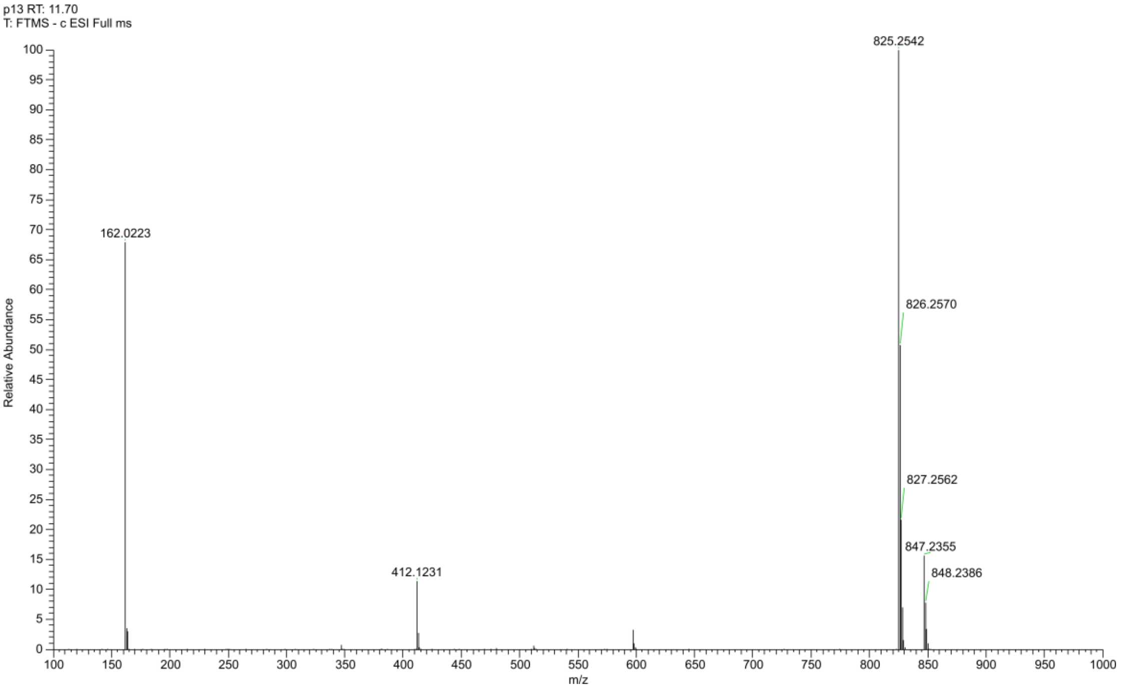

Figure S41. High resolution, negative mode HESI MS spectrum of the **IIIb-NAC-2** conjugate (t_r 11.70 min) formed in the sample of the pH 8.0 incubate. (m/z 412.1232 [(**IIIb-NAC**)-H]$^-$, m/z 825.2541 [(**IIIb-NAC**)$_2$-H]$^-$, m/z 847.2354 [(**IIIb-NAC**)$_2$+Na-2H]$^-$).

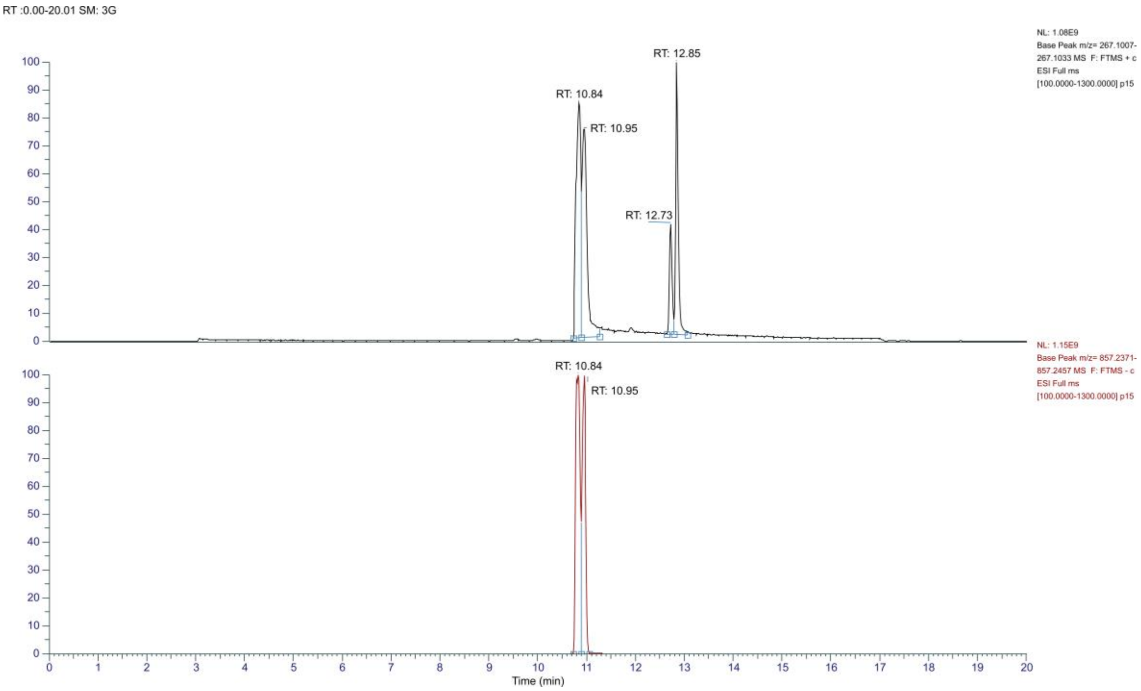

Figure S42. High resolution, HPLC-MS chromatograms of **IIIc**/**NAC** incubate (pH 8.0 sample).
Upper panel: positive mode m/z 267.1022 [**IIIc**+H]$^+$ t_r 12.73 min: (*Z*)-**IIIc** and t_r 12.85 min: (*E*)-**IIIc**.
Lower panel: negative mode m/z 857.2437 [(**IIIc-NAC**)$_2$-H]$^-$ t_r 10.84 min: **IIIc-NAC-1**, t_r 10.95 min: **IIIc-NAC-2**.

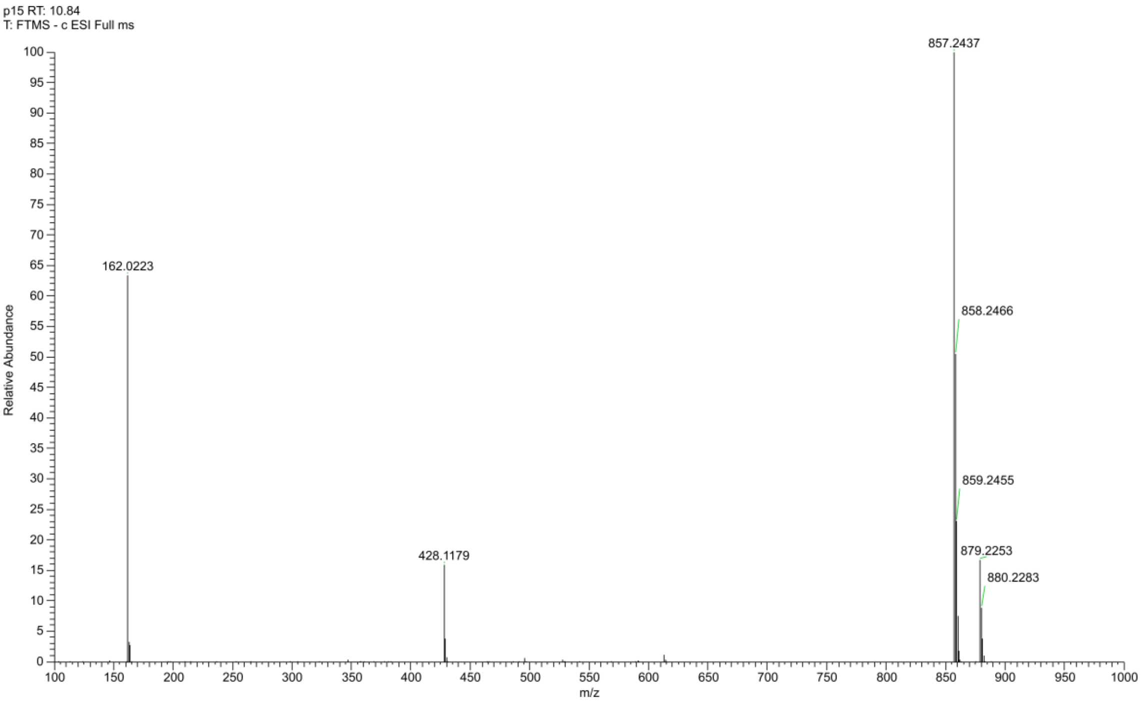

Figure S43. High resolution, negative mode HESI MS spectrum of the **IIIc-NAC-1** conjugate (t_r 10.84 min) formed in the sample of the pH 8.0 incubate. (m/z 428.1179 [(**IIIc-NAC**)-H]$^-$, m/z 857.2437 [(**IIIc-NAC**)$_2$-H]$^-$, m/z 879.2251 [(**IIIc-NAC**)$_2$+Na-2H]$^-$).

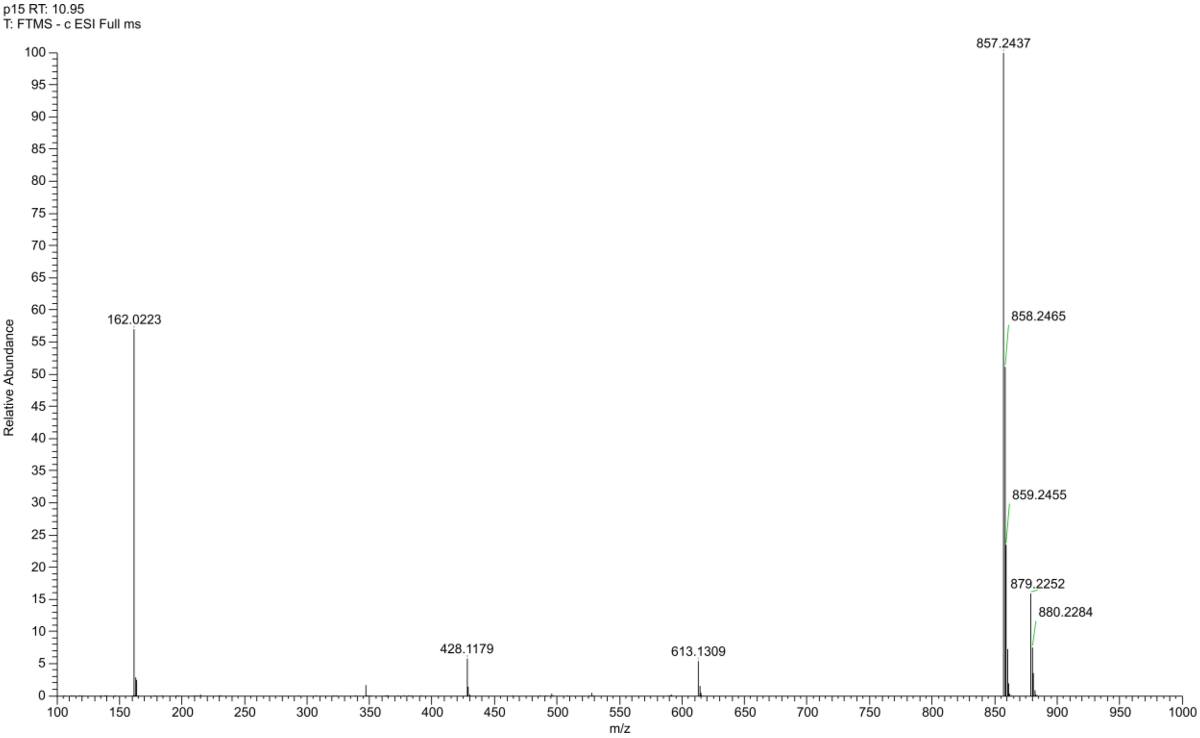

Figure S44. High resolution, negative mode HESI MS spectrum of the **IIIc-NAC-2** conjugate (t_r 10.95 min) formed in the sample of the pH 8.0 incubate. (m/z 428.1179 [(**IIIc-NAC**)-H]$^-$, m/z 857.2437 [(**IIIc-NAC**)$_2$-H]$^-$, m/z 879.2251 [(**IIIc-NAC**)$_2$+Na-2H]$^-$).

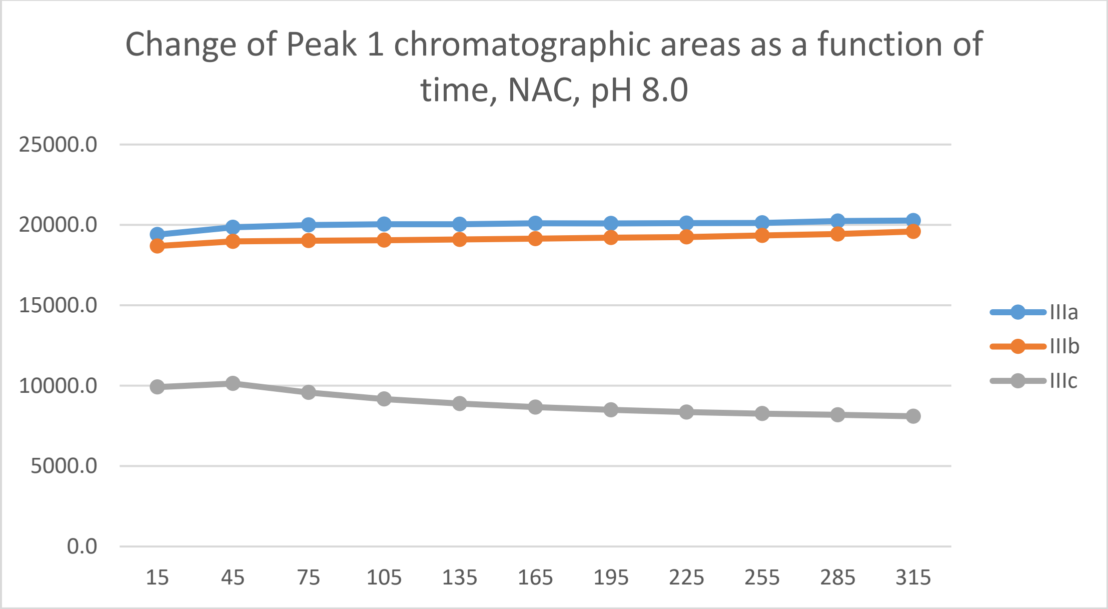

Figure S45. Change in the HPLC-UV chromatographic peak area of adduct-1 of **IIIa, IIIb,** and **IIIc** as a function of time (min) in the chalcone/NAC incubations at pH 8.0. Each data point represents the average of two independent measurements.

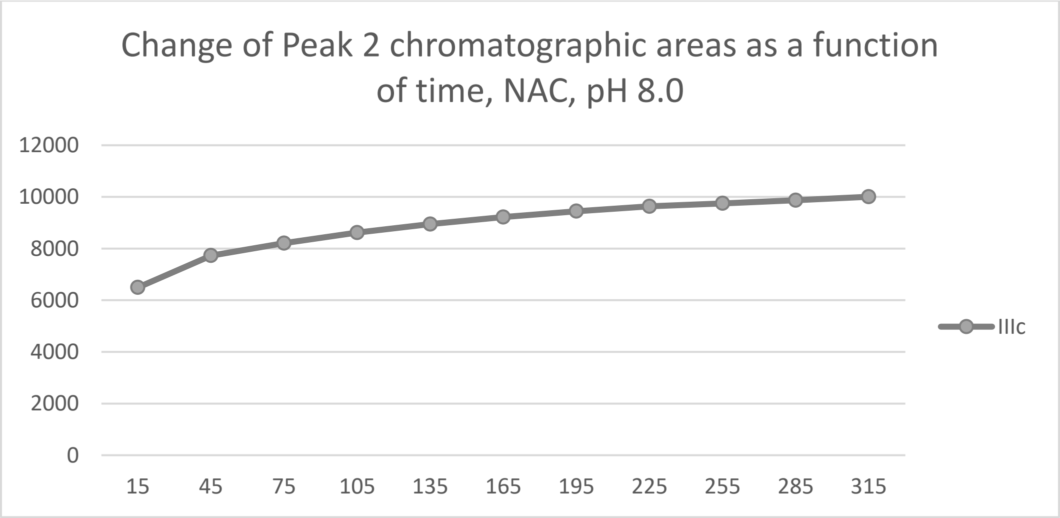

Figure S46. Change in the HPLC-UV chromatographic peak area of adduct-2 of **IIIc** as a function of time (min) in the chalcone/NAC incubations at pH 8.0. Each data point represents the average of two independent measurements.

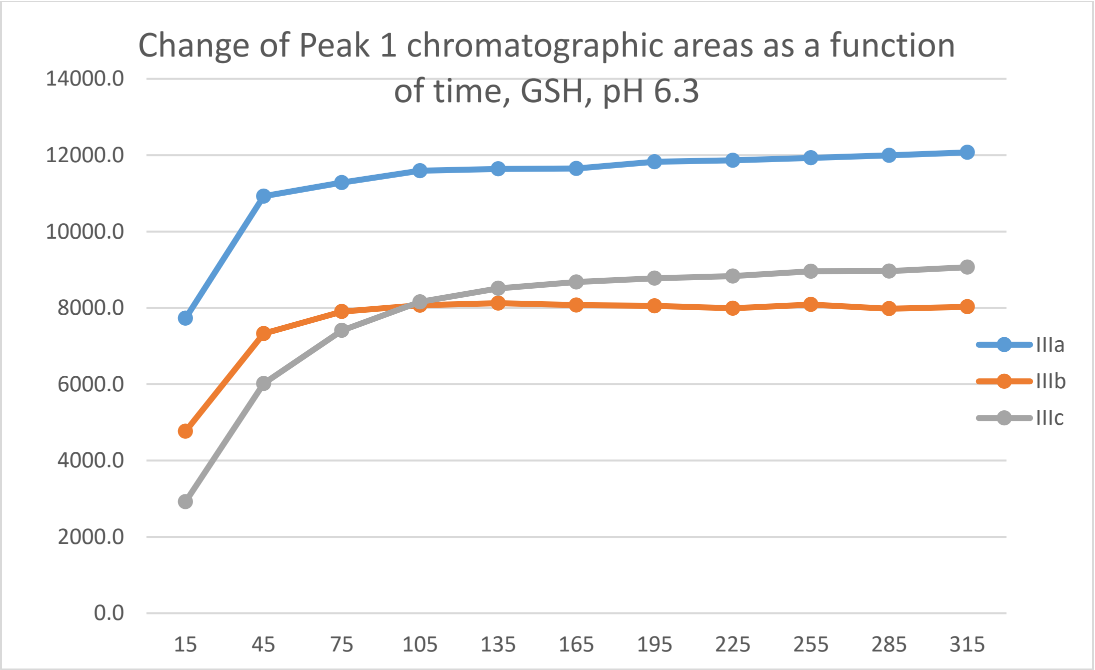

Figure S47. Change in the HPLC-UV chromatographic peak area of adduct-1 of **IIIa, IIIb,** and **IIIc** as a function of time (min) in the chalcone/GSH incubations at pH 6.3. Each data point represents the average of two independent measurements.

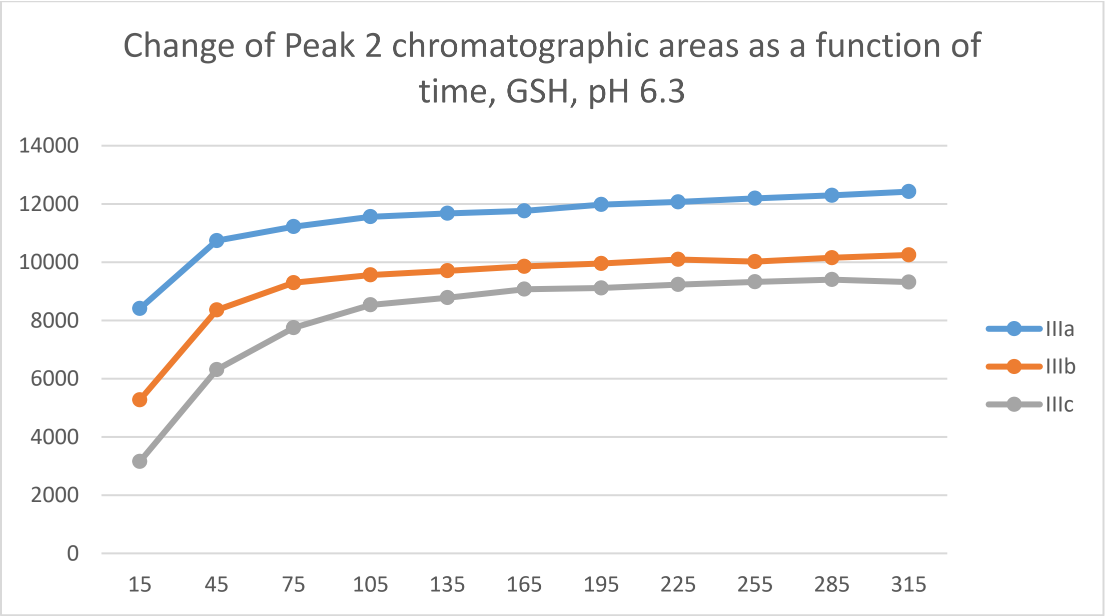

Figure S48. Change in the HPLC-UV chromatographic peak area of adduct-2 of **IIIa, IIIb,** and **IIIc** as a function of time (min) in the chalcone/GSH incubations at pH 6.3. Each data point represents the average of two independent measurements.

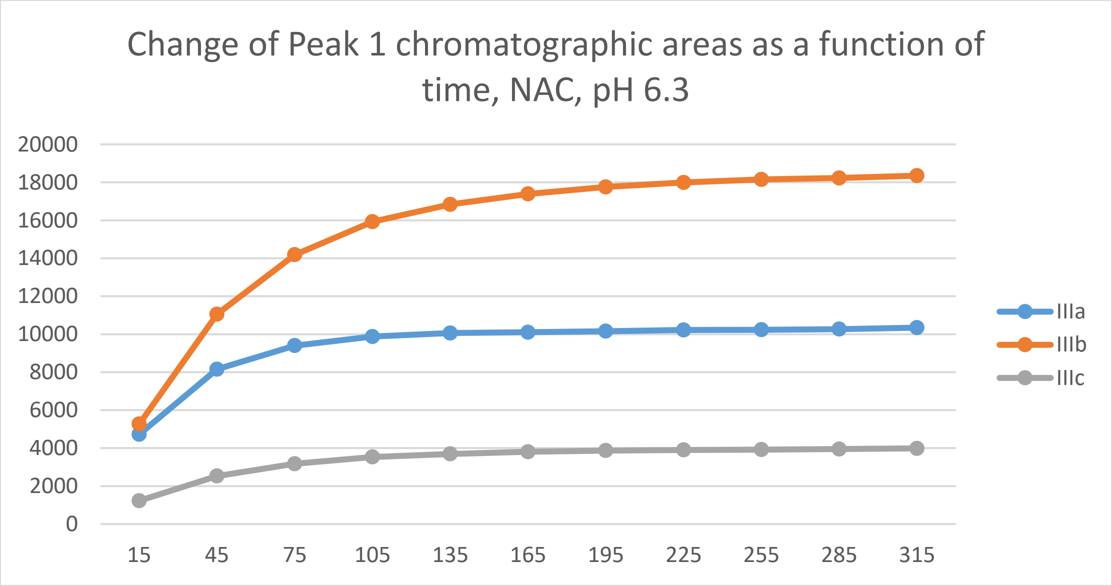

Figure S49. Change in the HPLC-UV chromatographic peak area of adduct-1 of **IIIa, IIIb,** and **IIIc** as a function of time (min) in the chalcone/NAC incubations at pH 6.3. Each data point represents the average of two independent measurements.

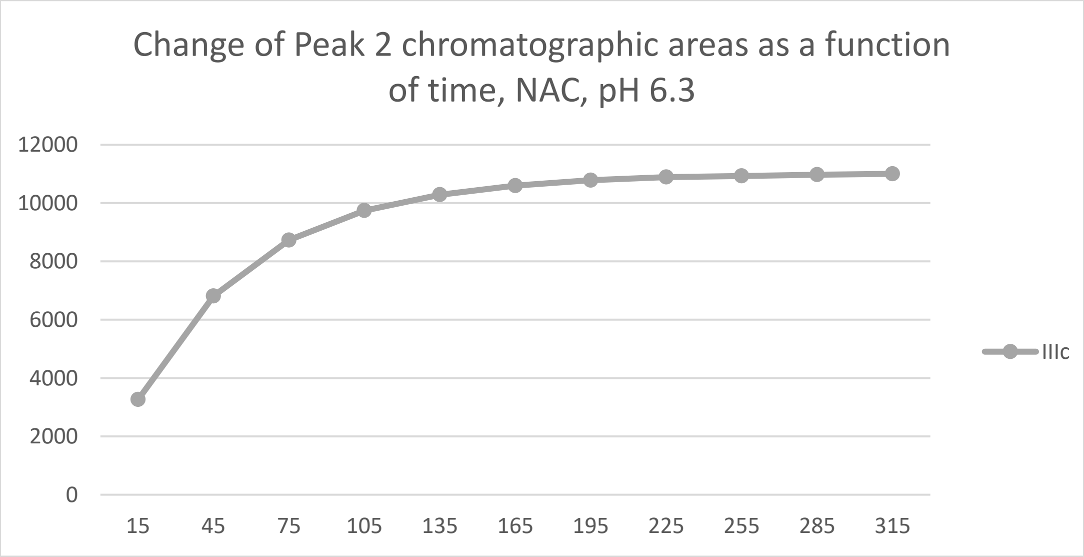

Figure S50. Change in the HPLC-UV chromatographic peak area of adduct 1 of **IIIa, IIIb,** and **IIIc** as a function of time (min) in the chalcone/NAC incubations at pH 6.3. Each data point represents the average of two independent measurements.

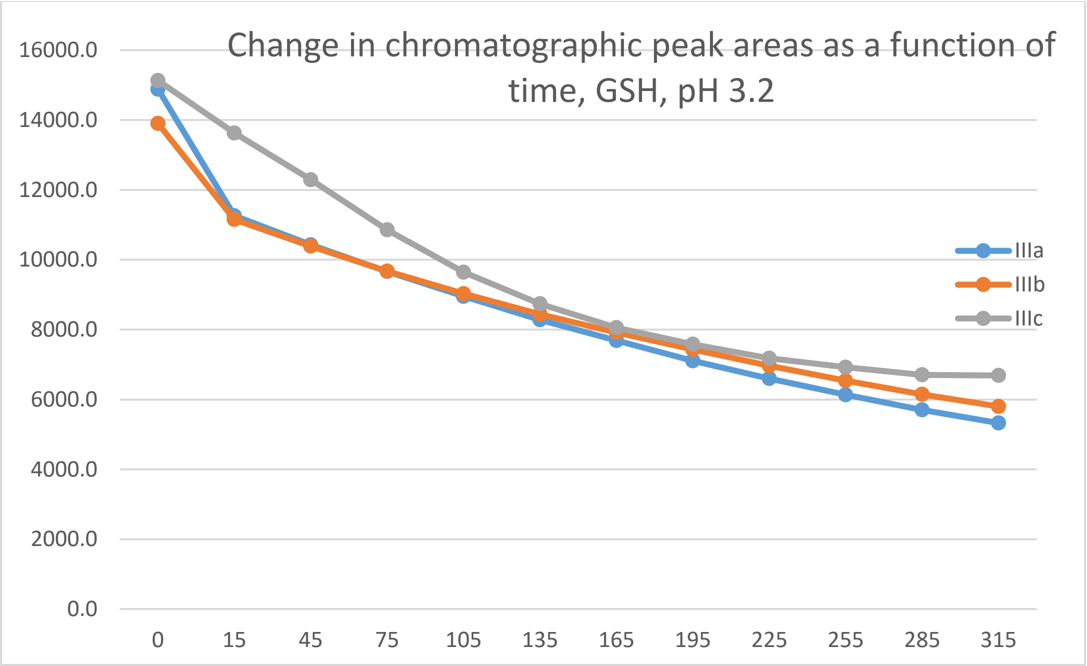

Figure S51. Change in the HPLC-UV chromatographic peak area of chalcones **IIIa-c** as a function of time (min) in the chalcone/GSH incubations at pH 3.2. Each data point represents the average of two independent measurements.

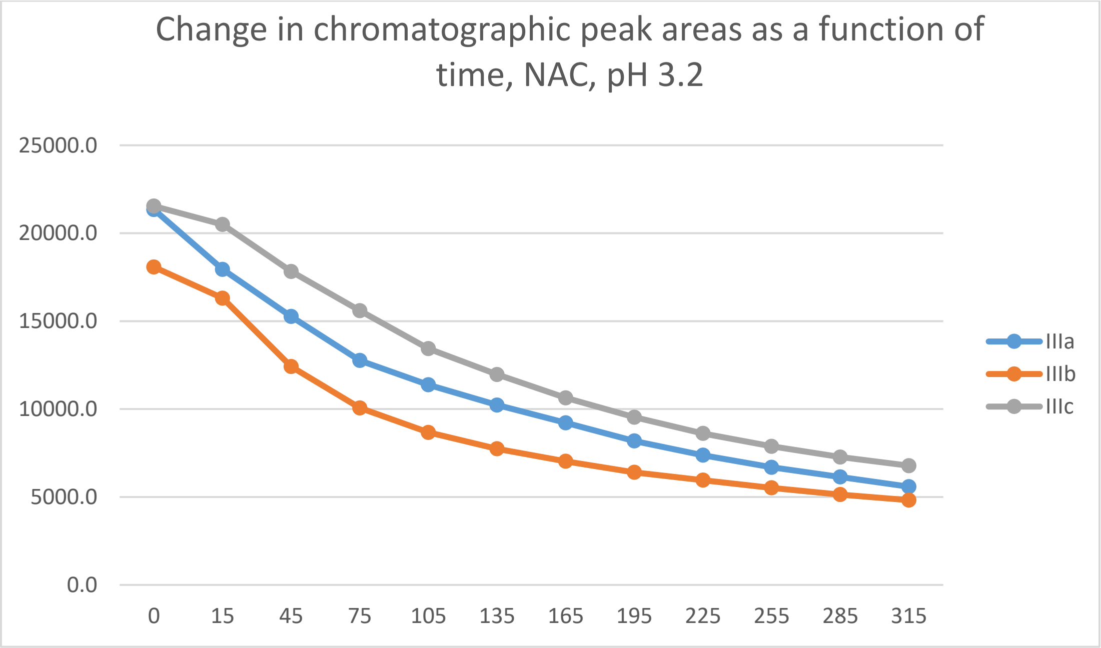

Figure S52. Change in the HPLC-UV chromatographic peak area of chalcones **IIIa-c** as a function of time (min) in the chalcone/NAC incubations at pH 3.2. Each data point represents the average of two independent measurements.

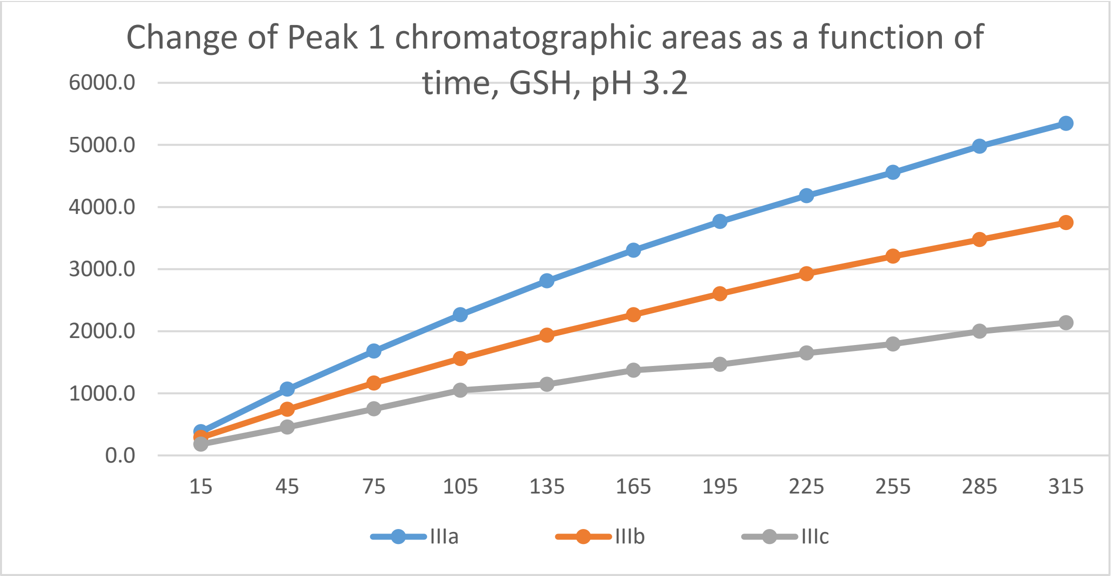

Figure S53. Change in the HPLC-UV chromatographic peak area of adduct-1 of **IIIa, IIIb,** and **IIIc** as a function of time (min) in the chalcone/GSH incubations at pH 3.2. Each data point represents the average of two independent measurements.

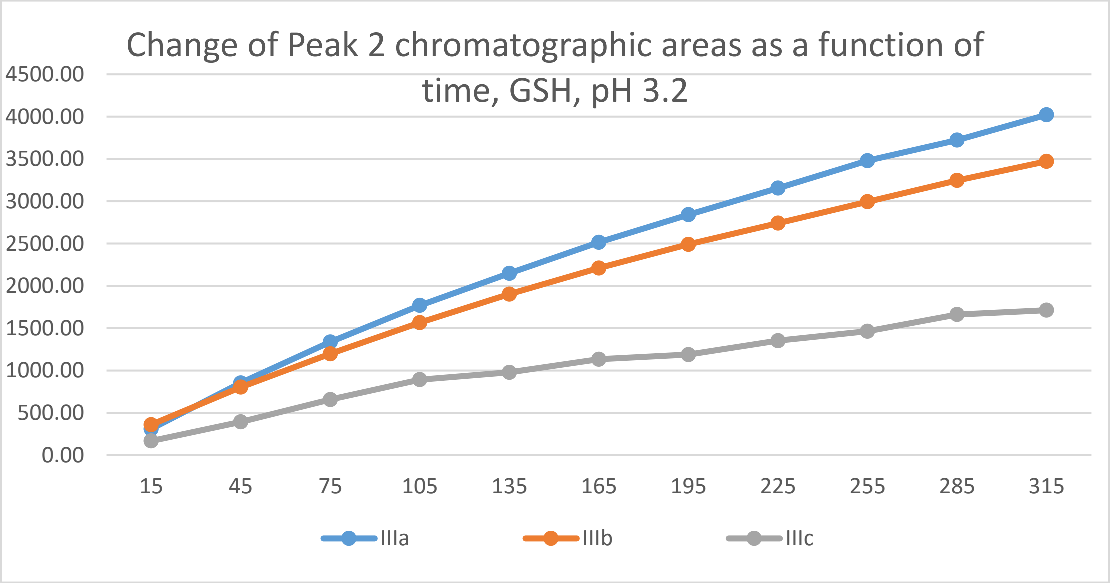

Figure S54. Change in the HPLC-UV chromatographic peak area of adduct-2 of **IIIa, IIIb,** and **IIIc** as a function of time (min) in the chalcone/GSH incubations at pH 3.2. Each data point represents the average of two independent measurements.

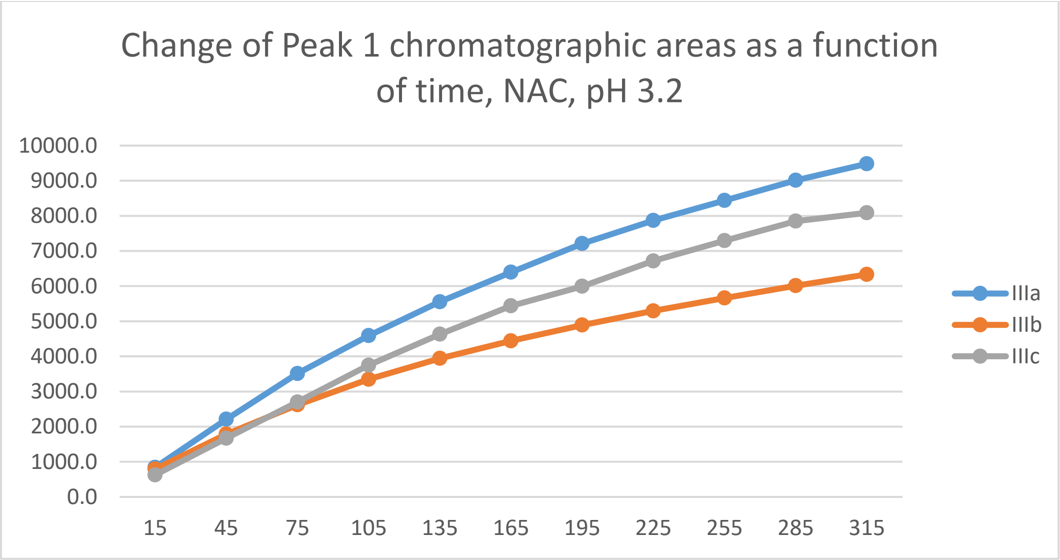

Figure S55. Change in the HPLC-UV chromatographic peak area of adduct-1 of **IIIa, IIIb,** and **IIIc** as a function of time (min) in the chalcone/NAC incubations at pH 3.2. Each data point represents the average of two independent measurements.

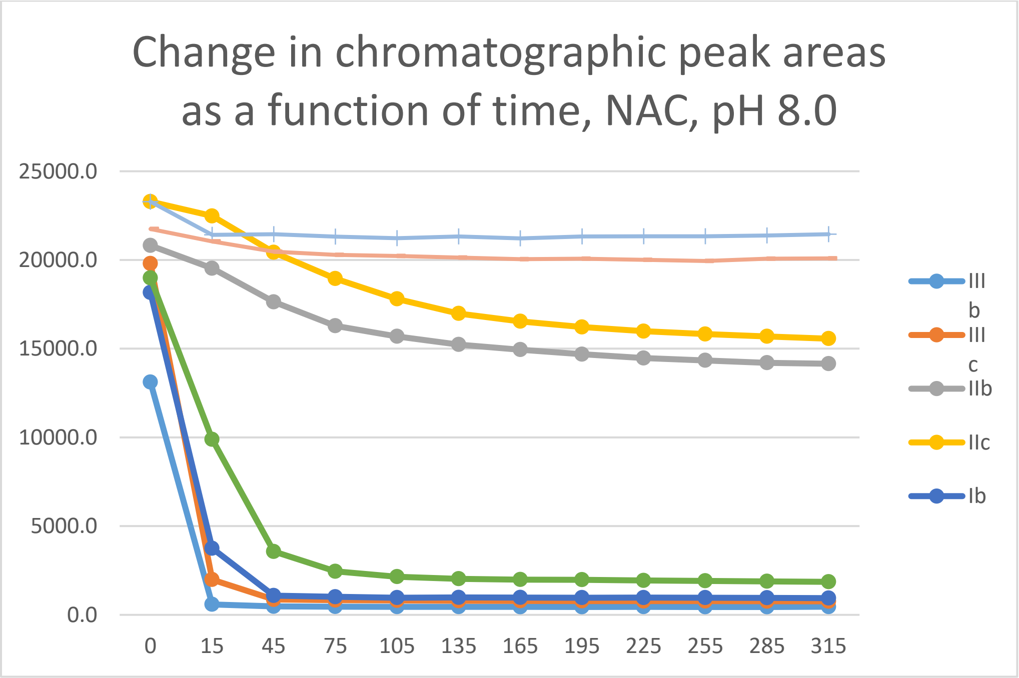

Figure S56. Comparison of change of the HPLC-UV chromatographic peak areas of selected chalcones (**Ib**, **Ic**) and cyclic chalcone analogs (**IIb**, **IIc**; **IIb**, **IIc**; and **IVb**, **IVc**) as a function of time (min) in the chalcone/NAC incubations, pH 8.0. Each data point represents the average of two independent measurements.

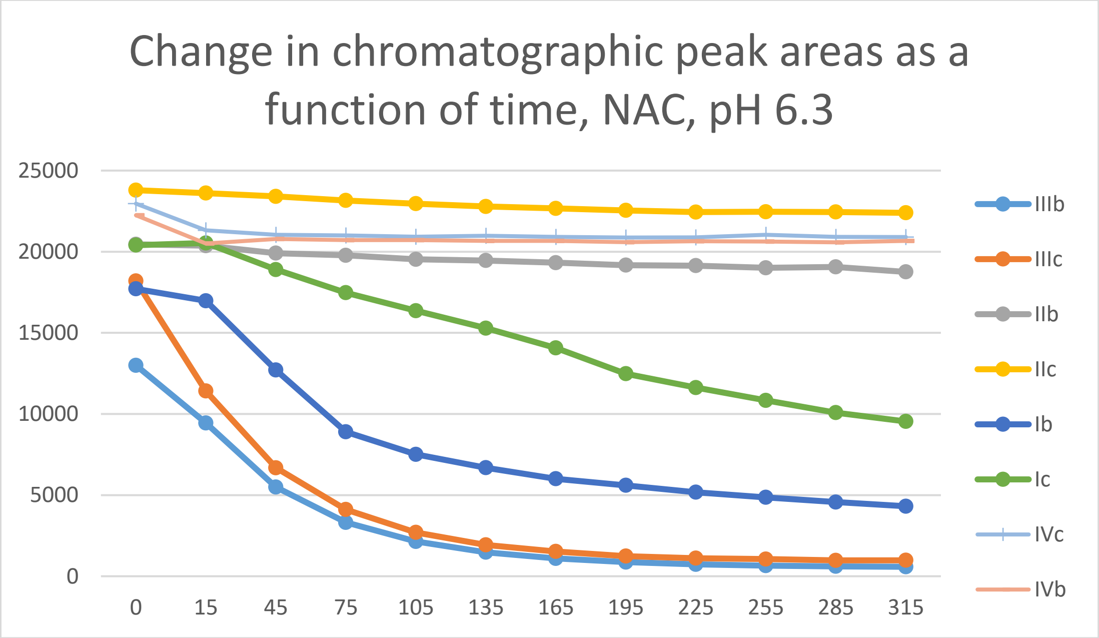

Figure S57. Comparison of change of the HPLC-UV chromatographic peak areas of selected chalcones (**Ib**, **Ic**) and cyclic chalcone analogs (**IIb**, **IIc**; **IIb**, **IIc**; and **IVb**, **IVc**) as a function of time (min) in the chalcone/NAC incubations, pH 6.3. Each data point represents the average of two independent measurements.

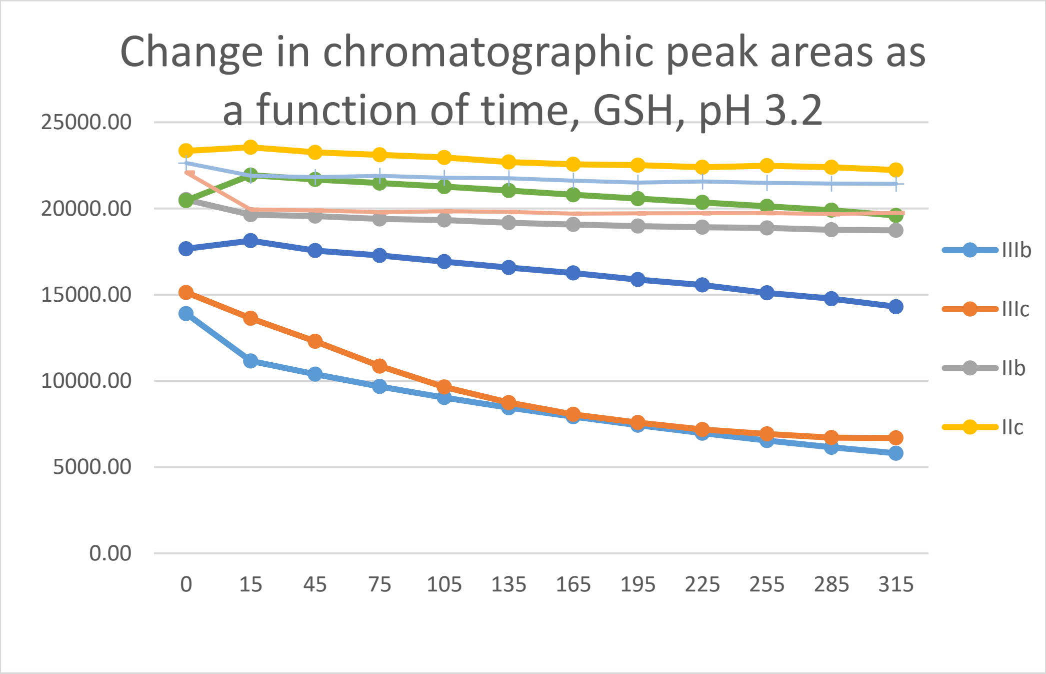

Figure S58. Comparison of change of the HPLC-UV chromatographic peak areas of selected chalcones (**Ib**, **Ic**) and cyclic chalcone analogs (**IIb**, **IIc**; **IIb**, **IIc**; and **IVb**, **IVc**) as a function of time in the chalcone-GSH incubations, pH 3.2. Each data point represents the average of two independent measurements.

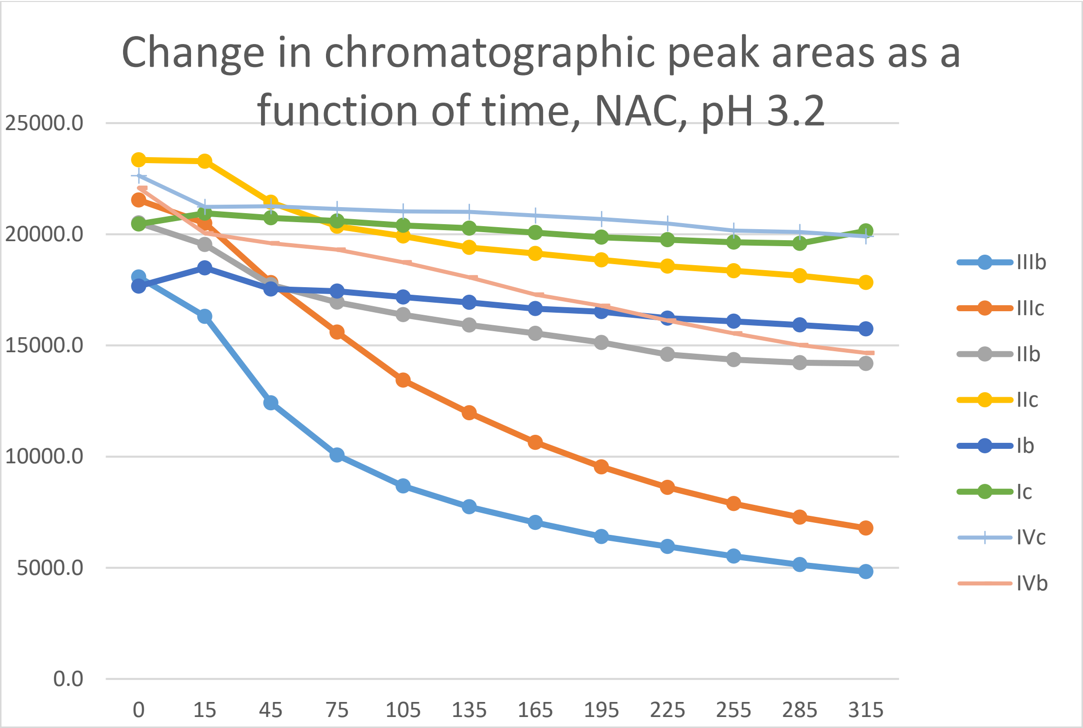

Figure S59. Comparison of change of the HPLC-UV chromatographic peak areas of selected chalcones (**Ib**, **Ic**) and cyclic chalcone analogs (**IIb**, **IIc**; **IIb**, **IIc**; and **IVb**, **IVc**) as a function of time in the chalcone-NAC incubations, pH 3.2. Each data point represents the average of two independent measurements.
